# Synthesis, X-ray Crystal Structure, Anticancer, Hirshfeld Surface Analysis, DFT, TD-DFT, ADMET, and Molecular Docking of 3-Phenyl-1,2,4-triazolo[3,4-h]-13,4-thiaza-11-crown-4

**DOI:** 10.3390/molecules28073166

**Published:** 2023-04-02

**Authors:** Fatima Lazrak, Sanae Lahmidi, El Hassane Anouar, Mohammed M. Alanazi, Ashwag S. Alanazi, El Mokhtar Essassi, Joel T. Mague

**Affiliations:** 1 Laboratory of Medicinal Chemistry, Drug Sciences Research Center, Faculty of Medicine and Pharmacy, Mohammed V University in Rabat, Rabat 10106, Morocco; 2 Laboratory of Heterocyclic Organic Chemistry, Department of Chemistry, Faculty of Sciences, Mohammed V University in Rabat, Rabat 10106, Morocco; lahmidi_sanae@yahoo.fr (S.L.); emessassi@yahoo.fr (E.M.E.); 3 Department of Chemistry, College of Science and Humanities in Al-Kharj, Prince Sattam bin Abdulaziz University, Al-Kharj 11942, Saudi Arabia; 4 Department of Pharmaceutical Chemistry, College of Pharmacy, King Saud University, Riyadh 11451, Saudi Arabia; mmalanazi@ksu.edu.sa; 5 Department of Pharmaceutical Sciences, College of Pharmacy, Princess Nourah bint Abdulrahman University, Riyadh 11671, Saudi Arabia; asalanzi@pnu.edu.sa; 6 Department of Chemistry, Tulane University, New Orleans, LA 70118, USA

**Keywords:** triazole, dihalogeno triethylene glycol, phase transfer catalysis, crown ether, X-ray, DFT, molecular docking

## Abstract

In this work, we describe the synthesis of new macrocycles derived from 3-phenyl-1,2,4-triazole-5-thione **1** in a heterogeneous medium using liquid–solid phase transfer catalysis (PTC) conditions. The structures of the two compounds (**3** and **4**) isolated were elucidated based on spectral data (^1^H-NMR, ^13^C-NMR) and confirmed in the case of 3-phenyl-1,2,4-triazolo [3,4-h]-13,4--thiaza-11-crown-4 (**3**) by a single-crystal X-ray diffraction analysis. Furthermore, the experimental spectral and the X-ray geometrical parameters were compared with their corresponding predicted ones obtained at the B3LYP/6-311++G(d,p) level of theory. The intercontacts between crystal units were investigated through Hirshfeld surface analysis. The drug-like macrocycles were predicted using ADMET and drug-likeness properties, which showed that **3** may act as an inhibitor of DNA-dependent protein kinase (DNA-PK). This assumption was confirmed by the well-binding fitting of **3** into the binding site of DNA-PK and the formation of a stable **3**-DNA-PK complex with a binding energy of −7 kcal-mol^−1^. Finally, the anticancer activity of **3** was assessed by an MTT assay against A549 cells, which showed that **3** has moderate anticancer activity compared to that of the doxorubicin reference drug.

## 1. Introduction

Derivatives of 1,2,4-triazole-3(5)-thione have received considerable attention in recent years due to their useful applications in different areas, such as pharmacology [1,2], biology [3], and industry [4]. Some compounds are known as antibacterial [5], anti-inflammatory [6], antimicrobial [7], anticonvulsant [8], antifungal [9,10], anticancer [11,12], antioxidant [13], and analgesic agents [14]; others are found in pesticide residues [15]. Moreover, compounds containing 1,2,4-triazole-thione moiety possess corrosion-inhibition activities for certain metals and alloys in various corrosive media [16,17]. Furthermore, crown ethers and their nitrogenous or sulfur derivatives constitute effective and versatile heterocyclic compounds characterized by their ability to form selective and stable complexes with various organic and inorganic cations such as metal ions—in particular the s block, the transition metals, and lanthanide ions [18,19]. They present efficient applications in various chemical and biochemical fields, such as recognition [20]. In transformation, they can be used as supramolecular catalysts [21]. They also exhibit potential biological and pharmacological properties [22], such as determining the concentration of sodium and potassium in blood [23] and transporting ions across a cell membrane [24].

**Figure 1:**
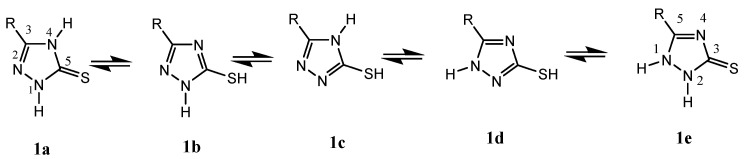
Tautomerism of 1,2,4-triazole thione.

The concept of tautomerization is called tautomerism, which results in the formal migration of a hydrogen atom or proton, accompanied by a switch of a single bond and its adjacent double bond. Therefore, 1,2,4-triazole thione presents five tautomeric forms—wo tautomers of type thione **1a**,**1e** and **3** tautomers of type thiol **1b**–**d** (Figure 1). Thus, 1,2,4-triazole thione derivatives are characterized by nucleophilic and electrophilic reactions and regarded as versatile synthetic intermediates for the preparation of several biologically active N-bridged and S-bridged heterocyclic compounds [25].

**Scheme 1:**
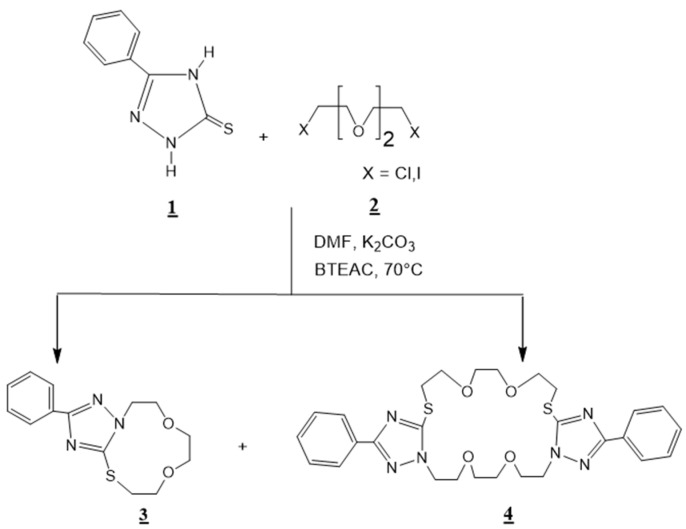
Synthesis of 3-phenyl-1,2,4-triazolo [3,4-h]-13,4-thiaza-11-crown-4: **3** and 3,14-diphenyl-bis-1,2,4-triazolo [3,4-s][4,3-g]-17,26,16,4-dithiadiaza-22-crown-8 **4**.

In continuation of our research works on the synthesis and spectral characterization of new crown ethers condensed to several heterocyclic compounds, including 1,2,4-triazole [26]; 1,5-benzodiazepine-2,4-dione [19]; quinoxaline [27,28]; pyrazolyl-benzimidazole [29,30], in this current work we describe the synthesis of 3-phenyl-1,2,4-triazolo [3,4-h]-13,4-thiaza-11-crown-4 **3**, and 3,14-diphenyl-bis-1,2,4-triazolo [3,4-s][4,3-g]-17,26,16,4-dithiadiaza-22-crown-8 **4** (Scheme 1).

## 2. Results and Discussion

### 2.1. Synthesis of 3-Phenyl-1,2,4-triazolo [3,4-h]-13,4-thiaza-11-crown-4: 3, and 3,14-diphenyl-bis-1,2,4-triazolo [3,4-s][4,3-g]-17,26,16,4-dithiadiaza-22-crown-8 4

To a solution of 1,2,4–triazole-5-thione **1** (1.01 g, 0.01 mol) and dichlorotriethylene glycol **2** (1.87 g, 0.01 mol) in 60 ml of N,N-dimethylformamide (DMF), we added potassium carbonate ( 4.15 g, 0.03 mol) and (0.182 g, 0.0008 moles) of benzyltriethyl ammonium chloride BTEAC (catalyst). The mixture was stirred for 24 h at 70 °C, then filtered and dried. The residue was chromatographed over silica (hexane/ethyl acetate: 3/1) as eluant to yield the macrocyclic compounds **3** and **4** (Scheme 1).

These results confirmed that the tautomeric form involved in the reaction studied was mainly**1b** of type thiol and showed that the N-1 triazolic and the sulfur atom of the sulfanyl group were the most reactive sites (Figure 1). The physical characteristics and the spectral data (^1^H NHR, ^13^C-NMR, and Mass Spectra) of compounds **3** and **4** are listed below.

#### 2.1.1. 3-Phenyl-1,2,4-triazolo [3,4-h]-13,4-thiaza-11-crown-4 (**3**)

Yield 65% (solid); M.p(°C)=. 115–117; ^1^H—NMR (250 MHz, CDCl_3_, δ(ppm)): δ = 4.43 (2H, N-CH2), 3.22 (2H, S-CH2), 3.54 (2H,CH2-O) 3,97 (2H CH2-O), 3.60 (4H, O-CH2CH2-O), 7.37–8.10 (5H, Ar-CH); ^13^C-NMR(400 MHz, CDCl_3_, δ(ppm)): δ = 37.49 (S-CH2), 48.39 (N-CH2), 68.76, 69.12, 69.17, 72.19 (CH2-O), 126.33, 128.57, 129.35 (CH Ar), 130.89 (Cq Ar), 151.84 (C5 triazole), 162.91 (C3 triazole); MS: m/z = 291.

#### 2.1.2. 3,14-Diphenyl-bis-1,2,4-triazolo [3,4-s][4,3-g]-17,26,16,4-dithiadiaza-22-crown-8 (**4**)

Yield 15%(solid); M.p (°C)=.161–163; ^1^H—NMR (250 MHz, CDCl_3_, δ(ppm)): δ = 4.34 (4H, N-CH2), 3.40 (4H, S-CH2), 3.77–3.88 (8H, CH2-O), 3.57 (8H, O-CH2CH2-O), 7.37–8.04 (10H, Ar-CH); ^13^C-NMR(400 MHz, CDCl_3_, δ(ppm)): δ = 34.06 (S-CH2), 49.00 (N-CH2), 69.25, 69.95, 70.26, 71.10 (CH2-O), 126.22, 128.57, 129.20 (CH Ar), 131.02 (Cq Ar) 153.40 (C5 triazole), 161.82 (C3 triazole); MS: m/z = 582.

**Figure 2:**
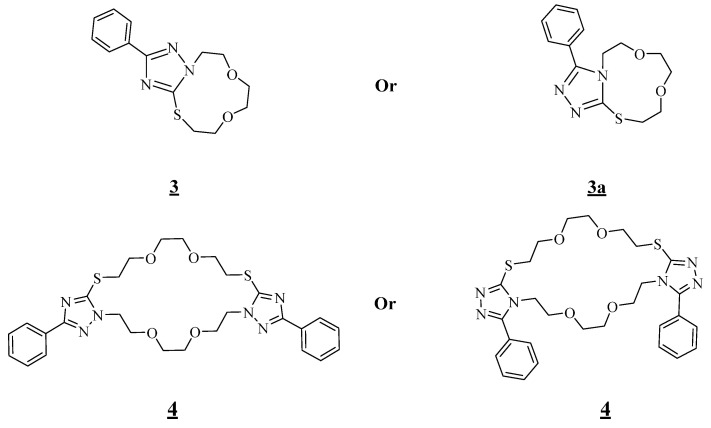
Possible isomeric forms of **3** and **4**.

It is worth mentioning that the isolated compounds **3** and **4 **can have other possible isomeric structures **3a** and **4a** involving the sulfanyl group as well as the nitrogen atom in the 4-position of the triazole moiety of the tautomeric form **1c** (Figure 2). The spectral data (^1^H NMR, ^13^C NMR, mass) for **3** and **4 **did not allow us to establish their structures with certainty. The NMR experimental spectra of **3** and **4** are provided in Figures S1–S4.

Thus, according to the literature it has been shown that the sulfur atom is responsible for the first nucleophilic attack [31] on dichloro-triethylene glycol, and the reactivity of the triazolic N1-H or N4-H nitrogen atoms depends on several factors such as the nature of the substituent in the 3-position of the triazole ring, the effect of the solvent, the basicity and the temperature of the reaction medium [32,33,34]. To confirm the structure of compound **3**, we undertook a single-crystal X-ray diffraction analysis, which showed that the macrocyclization of 1,2,4-triazole thione undergoes the tautomeric form **1b**, highlighting that the N-1 atom is more reactive than the N-4-nitrogen atom of the triazole moiety.

### 2.2. X-ray Crystallography

**Figure 3:**
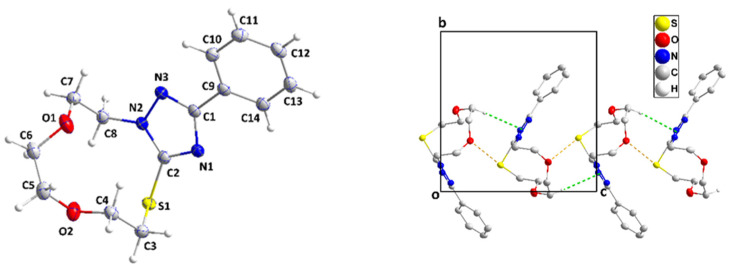
Perspective view of **3** with the labeling scheme and 50% probability ellipsoids (**left**). A portion of one chain of molecules viewed along the *a*-axis direction with C–H⋅⋅⋅π(ring) and O⋅⋅⋅S interactions depicted, respectively, by green and yellow dashed lines. Non-interacting hydrogen atoms are omitted for clarity (**right**).

**Figure 4:**
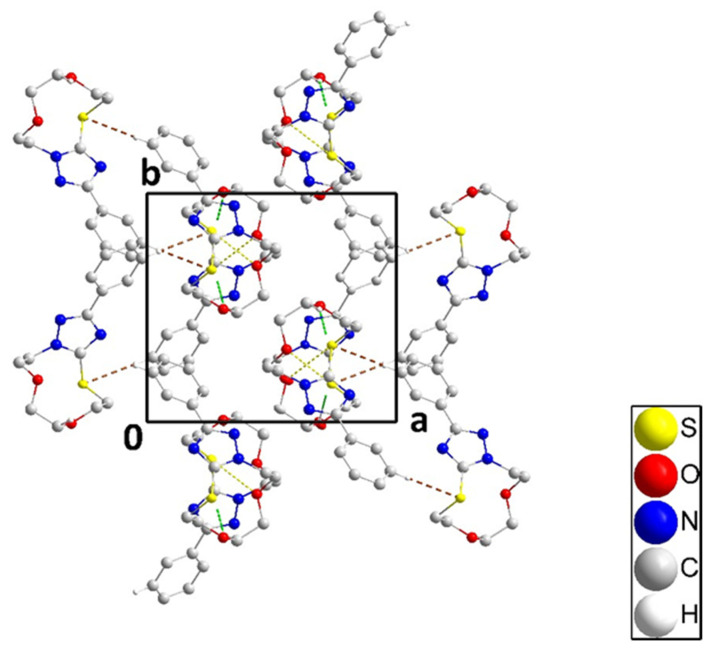
Packing of **3** viewed along the c-axis direction with C–H⋅⋅⋅S hydrogen bonds and C–H⋅⋅⋅π(ring) and O⋅⋅⋅S interactions depicted, respectively, by brown, green, and yellow dashed lines. Non-interacting hydrogen atoms are omitted for clarity.

The single-crystal X-ray diffraction analysis showed that the fused ring system of **3** adopts a zig-zag conformation (Figure 3 and cif file in supplementary section). A puckering analysis [35] of the 11-membered portion yielded the parameters Q(2), Q(3), Q(4), and Q(5) having values, respectively, of 0.1379(13), 1.2141(12), 0.9021(12) and 0.4116(13) Å, and the parameters φ(2), φ(3), φ(4), and φ(5) having values, respectively, of 275.7(5), 25.85(6), 349.44(8), and 99.44(16)°. The total puckering amplitude was 1.5736(11) Å. The dihedral angle between the mean planes of the C9⋅⋅⋅C14 and C1/N1/C2/N2/N3 rings was 21.77(7)°. All bond distances and interbond angles appeared as expected for the formulation given. In the crystal, C5–H5A⋅⋅⋅Cg1 interactions (Cg1 is the centroid of the triazole ring) with H5A⋅⋅⋅Cg1 = 2.80 Å and C5–H5A⋅⋅⋅Cg1 = 148° formed chains of molecules extending along the *c*-axis direction (Figure 3). Accompanying these were S1...O1^i^ (symmetry code: (i) *x*, -*y*+1/2, *z*-1/2) contacts which, at 3.1480(10) Å were 0.17 Å less than the sum of the respective van der Waals radii, suggesting an attractive interaction reinforcing the chain formation. The chains were connected by weak C13–H13⋅⋅⋅S1 hydrogen bonds (H13⋅⋅⋅S1 = 2.90 Å, C13–H13⋅⋅⋅S1 = 178°) across inversion centers (Figure 4).

### 2.3. DFT Results

**Figure 5:**
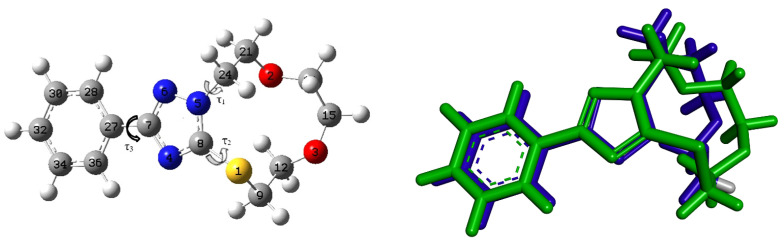
The optimized geometry of **3** (**left**) and its superposition (green) on the experimental structure (blue) (**right**).

The optimized geometry of **3** with its corresponding numbering is displayed in Figure 5. Figure 5 shows the superposition between the X-ray and the optimized geometries of **3**. Table 1 summarizes some of the experimental bond lengths, bond angles, and dihedral angles of **3**. The bond lengths are relatively well reproduced with a slight divergence between the experimental and computed values of less than 0.03 Å. Similarly, the bond angles are relatively well reproduced with a slight deviation to the experimental values of less than 3 degrees. The most important deviations were obtained for the torsion angles. In the optimized geometry of **3**, the aromatic ring was coplanar with the triazole ring with a torsion angle τ3 of one degree, while the experimental value was 21 degrees. The 11-membered ring was out of the molecular plane, with experimental torsion angles τ_1_ and τ_2_ to the triazole ring of 65 and 124 degrees (Figure 5). The corresponding computed torsion angles of τ1 and τ2 are 63 and 107 degrees, respectively. As mentioned in the methodology section, DFT calculations were at the B3LYP/ B3LYP/6-311++G(d,p) level of theory. The reliability of B3LYP to reproduce geometrical parameters accorded well with previous studies of Kumar et al., which reported that the geometrical parameters calculated using B3LYP method were in good agreement with the parameters obtained from X-ray crystallographic data, and that the B3LYP may provide the results much faster with accuracy in comparison to the other methods [36,37,38].

### 2.4. NMR Analysis

**Figure 6:**
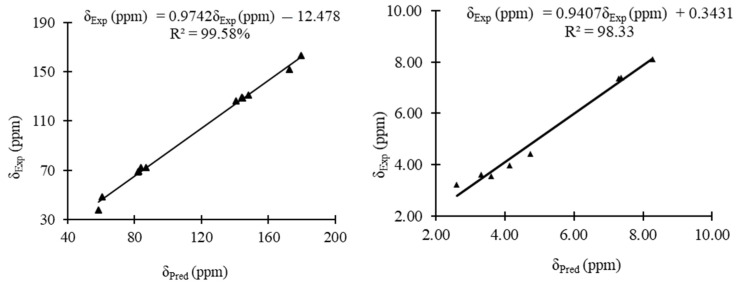
Linear correlation curves between the experimental ^13^C and ^1^H NMR chemical shifts of **3 **and their corresponding predicted ones.

The experimental and predicted ^13^C and ^1^H NMR shifts of **3** are shown in Table 2. The correlation curves between the experimental and predicted ^13^C and ^1^H NMR shifts for the condensed triazole **3** are displayed in Figure 6. The experimental chemical shifts for **3** are relatively well reproduced with correlation coefficients of 99.58 and 98.33% for ^13^C and ^1^H NMR chemical shifts, respectively. For the ^13^C NMR, a maximum deviation of 20 ppm was obtained for C9, while for ^1^H NMR a maximum deviation of 0.42 ppm was obtained for H9 to the experimental values (Table 2).

### 2.5. UV/Vis Spectrum

**Figure 7:**
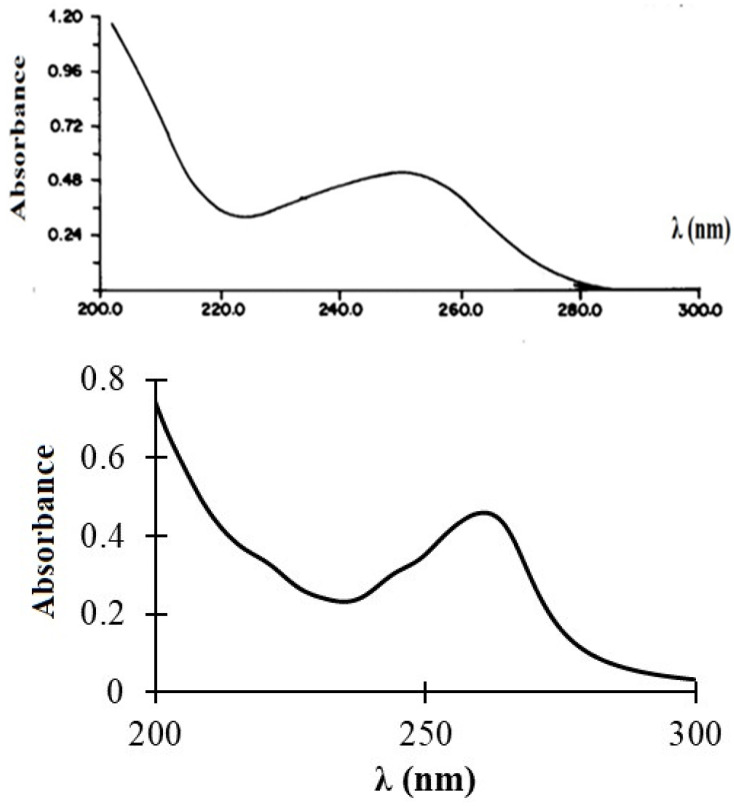
Experimental (**top**) and predicted (**bottom**) UV/Vis spectra of **3**.

The experimental and predicted UV/Vis spectra of compound **3** are shown in Figure 7. The experimental spectrum showed a broad absorption band at 250 nm of intensity 0.55. This band was relatively well reproduced within the TDDFT method, i.e., B3LYP/6-311++G(d,p), which is attributed to an electronic transition between the HOMO and LUMO orbitals of **3**.

### 2.6. Hirshfeld Surface Analysis

**Figure 8:**
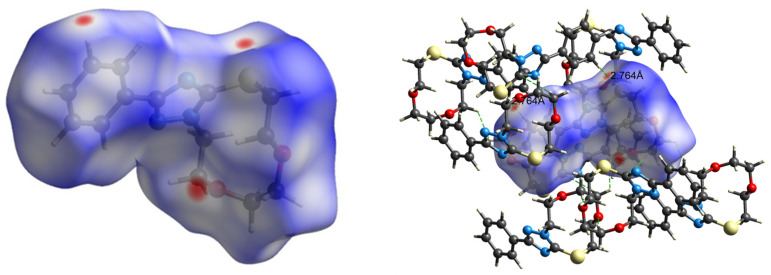
d_norm_ mapped on the Hirshfeld surface for visualizing the intermolecular hydrogen bonding interactions in **3**.

The intermolecular interactions observed in the crystals were usually analyzed via Hirshfeld surface analysis [39,40,41]. In a Hirshfeld surface, the electron density of a molecule in a crystal is divided into parts of continuous fragments, which occupy the crystal space. They can be easily generated using the Crystal Explorer software, as mentioned in methodology section [42]. The Hirshfeld surface (HS) mapped over d_norm_ for **3** is displayed in Figure 8. Internal and external (d_i_ and d_e_) contact distances from the Hirshfeld surface to the nearest atom inside and the outside enables the analysis of the intermolecular interactions through the mapping of d_norm_. The red spots on the HS indicate the intermolecular interactions (intercontacts) in the crystalline environment that involve contacts less than the sum of the van der Waals radii of the respective element (Figure 8). The blue color describes the contacts, which are longer than Van der Waal’s radii, while the white-colored area denotes contacts of the order of Van der Waal’s radii. 

**Figure 9:**
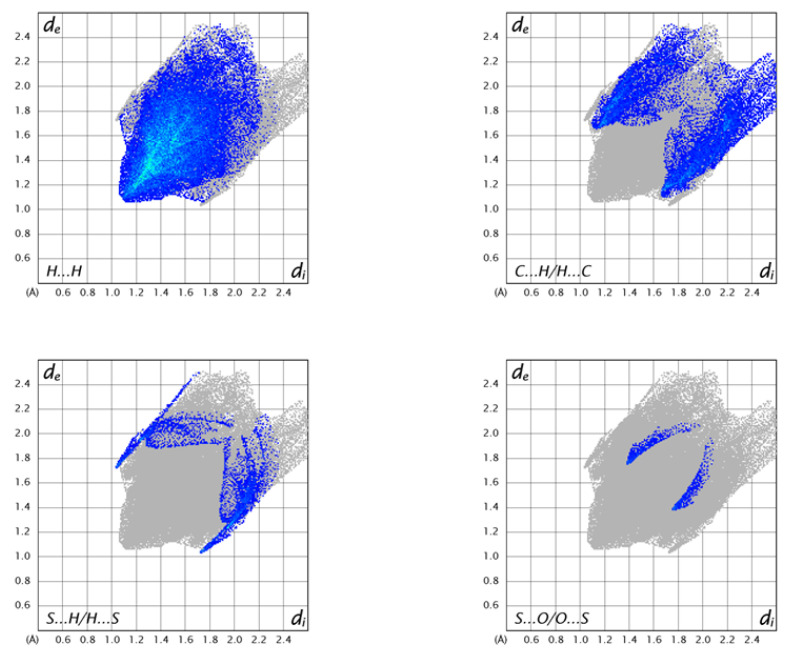
This is a figure. Schemes follow the same formatting.

The 2D fingerprint plots of **3** are shown in Figure 9. The highest number of intermolecular contacts in the crystal were H^…^H (54.4% of the total) and C⋅⋅⋅H/H⋅⋅⋅C (18.0% of the total) (Figure 9). The S⋅⋅⋅H intercontacts contributed 5.9%, and they were formed between the lone pair of the sulfur atom and an H atom of the aromatic ring (Figure 8). As the S1⋅⋅⋅H13 distance of 2.90 Å to the molecule at −*x*+2, −*y*+1, −*z*+1 was 0.10 Å less than the sum of the van der Waals of H and S, this can be considered a weak C–H⋅⋅⋅S hydrogen bond. Finally, there was the S⋅⋅⋅O contact of 3.148(1) Å, which was 0.17 Å less than the sum of the van der Waals radii and contributed 1.5% to the overall contacts.

### 2.7. ADMET and Drug-Likeness Prediction of the Synthesized Compound

**Figure 10:**
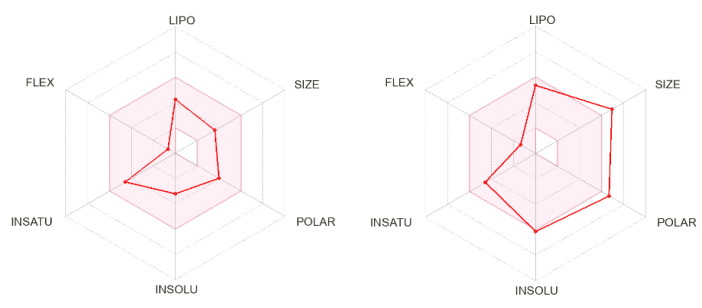
Bioavailability radars of **3** (**left**) and **4** (**right**).

**Figure 11:**
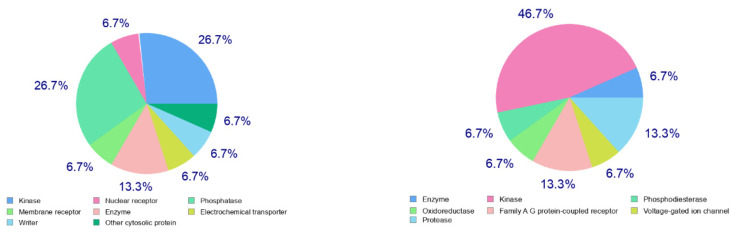
Predicted biological targets of **3** (left) and **4** (right).

**Figure 12:**
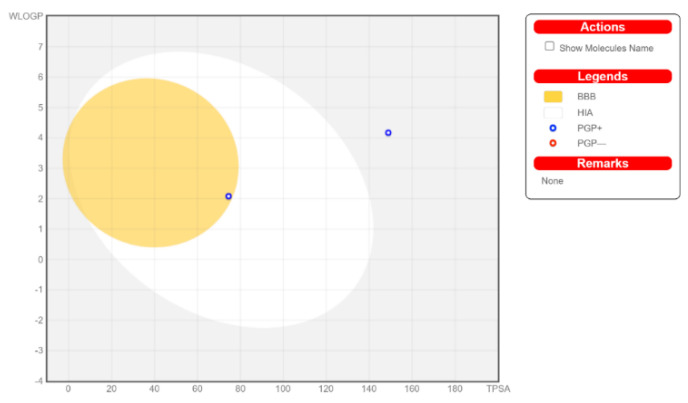
Boiled-egg model of **3** and **4**.

The calculated physicochemical properties, lipophilicity, water solubility, pharmacokinetics, drug-likeness, and medicinal chemistry properties of **3** and **4** are provided in the Supplementary Materials (Tables S1–S5). The drug-like properties reveal that **3** obeyed Lipinski’s rule of five, with molar mass less than 500 g-mol^−1^, and lipophilicity (MlogP) less than 1.55 (Tables S2 and S5), while for **4**, no drug-like property was validated, as these rules were violated (Table S5). For **3**, the calculated topological surface area (TPSA) was 74.5 Å², which was in the range of 78–125 Å² (Table S1), indicating moderate oral absorption with a good bioavailability score of 0.55 (Table S1 and Table S5). The bioavailability was visualized using the bioavailability radars (Figure 10), with all the properties of **3** inside the pink area of the polygon, which suggested that **3** may have good oral bioavailability. The pharmacokinetics properties showed that **3** had high GI absorption (Table S4) and may have penetrated the blood–brain barrier (BBB) (Figure 11), but had low skin permeability with log Kp > −2.5 (Table S4). To estimate the biological targets and the possible side effects of **3** and **4**, their corresponding pie-charts were predicted (Figure 12), which showed that **3** may act as an inhibitor of DNA-dependent protein kinase (DNA-PK).

### 2.8. Molecular Molecular Docking Study

The ADMET study showed that **3** may act as an inhibitor of the DNA-dependent protein kinase (DNA-PK). For that, the binding affinity of **3** toward DNA-PK was examined by docking **3** into the binding site of DNA-PK and comparing the results with the original docked ligand (**OLig**) in DNA-PK. The calculated binding energies of the stable **3**-DNA-PK and **OLig**-DNA-PK complexes, the number of intermolecular hydrogen bonds established between the docked molecules and the active site residues of DNA-PK, and the number of closest amino acids that interact with docked compounds are displayed in Table 3.

**Figure 13:**
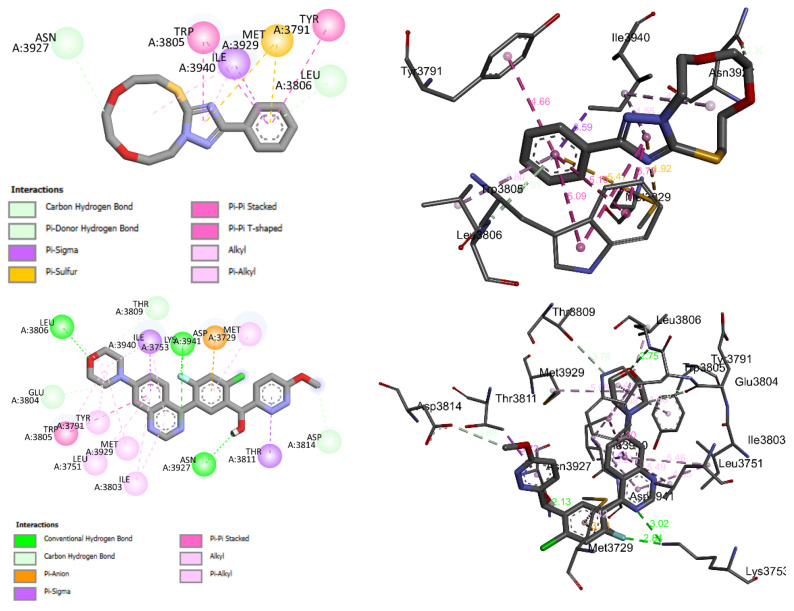
2D and 3D closest interactions between the active site residues of DNA-PK and **3** (**up**) and the original docked ligand **OLig** (**bottom**).

Compound **3** fit well into the binding site of DNA-PK and formed a stable complex **3**-DNA-PK with a binding energy of −7 kcal-mol^−1^. The negative binding energy may indicate that the inhibition of DNA-PK by **3** is thermodynamically favorable and spontaneous. Figure 13 shows the 2D and 3D binding interactions modes established in the stable complex formed between the **3** and the amino acid binding sites of DNA-PK. These interactions were of (i) carbon hydrogen bond between the C-H of the 11-membered ring of **3** and the keto group of ASN A3927 of DNA-PK, (ii) π-donor hydrogen between the π electrons of the aromatic and the lone pair of the nitrogen atom of the amino acid LEU A3806 of DNA-PK, (iii) π-σ, (iv) π-sulfur, (v) π-π stacking, (vi) π-π T-shaped, and (vii) alkyl and π-alkyl types (Figure 13). As can be seen in Table 3 and Figure 13, **3** showed moderate binding affinity compared with that of the original ligand docked into the binding site of DNA-PK.

### 2.9. Anticancer Activity against A459 Cell Line

**Figure 14:**
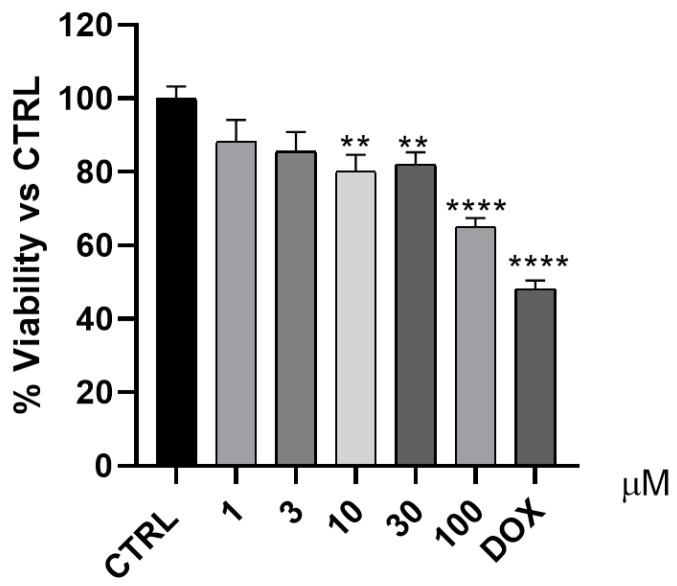
The antiproliferative effect of **3** was evaluated against the A549 cell line. ** *p* < 0.01 and **** *p* < 0.0001 indicates indicates statistically significant difference from the corre-sponding control (unteated A549 cells).

The anticancer effect of **3** was evaluated against A549 cell line by using the MTT colorimetric assay. Doxorubicin was used as a positive control, while five concentrations (1, 3, 10, 30, and 100 µM) of **3** were used to assess the cytotoxicity effect of **3** (Figure 14). As shown in Figure 14**,** compound **3** demonstrated moderate to weak anticancer activity compared to that of doxorubicin, which reduced the cell viability to approximately 50% at a single concentration of 0.5 µM.

## 3. Materials and Methods

### 3.1. General Procedure

Melting points were determined on the electrothermal apparatus and were not corrected. ^1^H and ^13^C NMR spectra were recorded on AC 250 MHz and 400 MHz Bruker instruments. The chemical shifts (δ) are provided in ppm relative to tetramethylsilane (TMS) as an internal standard. Mass spectra were recorded on Perkin Elmer Sciex API 100 and Varian MAT 311A instruments (Paul Sabatier University in Toulouse).

### 3.2. X-ray Crystallography

A colorless block-shaped crystal of **3** was mounted on a polymer loop with a drop of heavy oil and placed in a cold nitrogen stream on a Bruker D-8 Venture diffractometer. A hemisphere of intensity data was collected under the control of the *APEX4* software [43] and examination of 1057 reflections having I/σ(I) ≥ 20 and chosen from the full data set with *CELL_NOW* [44] showed that the crystal belonged to the monoclinic system and was twinned by a 180° rotation about the *b* axis. The raw data were converted to F^2^ values with *SAINT* [43] and the merging of equivalent reflections and the application of an empirical absorption correction were performed with *TWINABS* [45]. This last program also extracted a single-component reflection file from the twinned data for use in the structure solution, which was accomplished by dual space methods (*SHELXT* [46]). The model was refined by full-matrix, least-squares methods (*SHELXL* [47]) using the single-component data with hydrogen atoms included as riding contributions in idealized positions with isotropic displacement parameters tied to those of the attached atoms. At the end of this refinement, trial refinements with the single-component reflection file extracted from the twinned data with *TWINABS* and with the complete twinned data set showed the former to be superior as judged by lower values of R1, *w*R2, the su’s on the derived parameters, and residual peaks in the final difference map. Crystallographic and refinement details are presented in Table 4.

### 3.3. Hirshfeld Surface Analysis

Hirshfeld surface and fingerprint plots of **3** were obtained using the Crystal Explorer 3.0 package [48]. The d_norm_ plots were mapped with a color scale in the range of −0.1090 au (blue) to 1.1867 au (red). The red spots on the Hirshfeld surface indicated the closest interactions between the atoms that may be involved in intermolecular hydrogen bonding. The 2D fingerprint plots were displayed using an expanded scale of 0.8–2.4Å.

### 3.4. DFT Calculations

The ground state (GS) geometry of **3** was optimized using the DFT method at the B3LYP/6-311++G(d,p) level of theory, as implemented in the Gaussian 16 software [48]. The frequency calculation confirmed that the optimized geometry was a true minimum [49]. The experimental X-ray coordinates of **3** were used as input Z-matrix parameters for the optimization of the geometry. The NMR magnetic isotropic shielding tensors (σ_iso_) were calculated using the Gauge Independent Atomic Orbital (GIAO) method [50]. The isotropic chemical shifts δ_iso_(X) to the reference tetramethylsilane (Si(CH_3_)_4_) were measured, where δ_iso_(X) = σ_TMS_(X) − σ_iso_(X). The predicted chemical shifts were calculated using the equation δ_exp_ = aδ_cal_ + b, where δ_cal_ = δ_iso_. The solvent effects were taken into consideration using the polarizable continuum model (PCM) with the acetonitrile and chloroform as solvents for UV/Vis and NMR predictions, respectively [51].

### 3.5. In Silico ADMET and Drug-Likeness Properties

The absorption, distribution, metabolism, excretion, and toxicity in and through the human body (ADMET) pharmacokinetics and the physico-chemical properties of **3** were predicted using the online Swiss ADME tool (http://www.swissadme.ch/ (accessed on 1 January 2023)). Molecular target predictions were determined using the web tool (http://www.swisstargetprediction.ch/ (accessed on 1 January 2023)).

### 3.6. Molecular Docking Study

The ADMET and drug-likeness prediction results suggested that **3** may act as a DNA-dependent protein kinase (DNA-PK). To confirm this assumption, a molecular docking study was carried out on **3** and **4** into the binding site of DNA-PK using the Autodock package[52]. The target DNA-PK and its original ligand were downloaded from the RCSB website (PDB code 7Z87) [53]. The molecular docking study was validated by re-docking of the original ligand in the binding site of DNA-PK, which yielded an RMSD of 0.99Å and a binding energy of −10.92 kcal-mol^−1^. Further steps of the docking of the original ligand and the trial molecules were reported in our previous study [54].

### 3.7. Cytotoxicity Assay

The antiproliferative effect **3** was assessed by an MTT assay against A549 cells. At first, DMEM medium with 10% fetal bovine serum was employed to grow the cells. Then, antibiotics (penicillin (100 units/mL) and streptomycin (100 µg/mL)) were added to the medium and the cells were incubated in a 96-well plate at a density of 1.0 × 104 cells/well at 37 °C under 5% CO_2_ for 24 h. Then, the cells were handled with five concentrations of **3** and left for another 24 h. In the next day, 20 µL of 5 mg/mL MTT solution was added and left for 2–4 h. Finally, 100 µL dimethyl sulfoxide (DMSO) was added to each well to dissolve the purple formazan formed. The color intensity was recorded and measured at a wavelength of 570 nm, using a plate reader BioTek EXL 800 (Agilent Technologies, Inc., Santa Clara, CA, USA). The percentage of relative viable cells was calculated by applying the following equation (A570 of treated specimens/A570 of the untreated specimen × 100).

## 4. Conclusions

The main focus of this research was to synthesize and characterize new 1,2,4-triazole-5-thione derivatives. The structures of these compounds were confirmed by spectral data (IR, ^1^H NMR, ^13^C NMR, and mass spectrometry). The results observed showed that the macrocyclization of 1,2,4-triazole thione involved the tautomeric form **1b**, highlighting that the sulfanyl group as well as the N-1 triazolic atom are the most reactive sites. The ADMET, drug-likeness, and molecular docking study revealed that **3** may act as a potent inhibitor of DNA-dependent protein kinase (DNA-PK). **3** showed moderate anticancer activity, compared to that of doxorubicin.

## Figures and Tables

**Table 1:** The selected experimental and optimized structure parameters of **3** were obtained at the DFT/ B3LYP/6-311++G(d,p) level of theory.

	Bond Lengths (Å)				Bond Angles (°)		
	X-ray	Cal	ΔL(Å)		X-ray	Cal	ΔA(°)
S1-C8	1.753	1.7671	0.013	C8-S1-C9	98.17	100.25	2.08
S1-C9	1.8244	1.8489	0.024	S1-C8-C5	123.32	123.99	0.67
O2-C18	1.4288	1.4286	0.000	C8-C5-C24	129.62	130.53	0.90
O2-C21	1.4187	1.4208	0.002	S1-C9-C12	113.69	114.59	0.90
O3-O12	1.4204	1.4255	0.005	C24-C21-O2	112.50	112.47	0.04
O3-O15	1.4264	1.4249	0.001	C21-O2-C18	114.85	114.88	0.03
C4-C7	1.3653	1.3640	0.001	C12-O3-C15	113.22	115.73	2.51
C4-C8	1.3303	1.3255	0.005	C6-C7-C27	121.86	122.93	1.08
C5-C6	1.3593	1.3529	0.006	C7-C27-C28	120.29	120.81	0.52
C5-C24	1.4611	1.4607	0.000	C6-C7-C27-C28	21.08	1.20	19.88
C7-C6	1.3330	1.3311	0.002	S1-C8-C5-C24	2.46	6.40	3.94
C7-C27	1.4713	1.4710	0.000	C8-C5-C24-C21	124.68	107.04	17.64
C27-C28	1.3989	1.4025	0.004	S1-C9-C12-O3	60.54	65.69	5.15
C27-C36	1.3948	1.4012	0.006	C12-O3-C15-C18	108.85	88.46	20.39

**Table 2:** Experimental and predicted ^13^C and ^1^H NMR chemical shifts for **3**.

	^13^C-NMR			^1^H-NMR			
	Cal	Exp	Δδ		Cal	Exp	Δδ
C7	176	162.9	12.76	H9	2.80	3.22	0.42
C8	169	151.8	17.22	H12	3.73	3.54	0.19
C9	57.8	37.49	20.28	H15/H18	3.47	3.6	0.13
C12	81.4	69.12	12.28	H21	4.24	3.97	0.27
C15	81	68.76	12.25	H24	4.80	4.43	0.37
C18	85.7	72.19	13.52	H28/H36	8.12	8.1	0.02
C21	82.4	72.19	10.2	H30/H34	7.26	7.4	0.14
C24	60	48.39	11.59	H32	7.20	7.37	0.17
C27	145	130.9	14.2				
C28/C36	138	126.3	11.73				
C30/34.	141	129.4	11.9				
C32	142	128.6	13.43				

**Table 3:** Inhibition concentrations, docking binding energies, hydrogen bonds, and the number of closest residues to the docked compounds **3** and **OLig**.

Free Binding Energy (kcal/mol)		H-Bonds (HBs)	Number of Closest Residues to the Docked Ligand in the Active Site ^1^
**3**	−6.98	0	9
**OLig**	−10.92	3	17

^1^ Number of amino acids in the active site that show binding interaction with **3** and **OLig**.

**Table 4:** Crystal and refinement data for **3**.

Identification Code	3	
Chemical formula	C_14_H_17_N_3_O_2_S	
Formula weight	291.36 g/mol	
Temperature	125(2) K	
Wavelength	1.54178 Å	
Crystal size	0.164 × 0.226 × 0.258 mm	
Crystal system	monoclinic	
Space group	P 1 2_1_/c 1	
Unit cell dimensions	a = 12.0933(5) Å	α = 90°
	b = 10.8160(4) Å	β = 102.7080(10)°
	c = 10.7940(4) Å	γ = 90°
Volume	1377.28(9) Å^3^	
Z	4	
Density (calculated)	1.405 g/cm^3^	
Absorption coefficient	2.139 mm^−1^	
F(000)	616	
Diffractometer	Bruker D8 VENTURE PHOTON 3 CPAD	
Radiation source	INCOATEC IμS micro--focus source (Cu-Kα, λ = 1.54178 Å)	
Theta range for data collection	3.75 to 72.33°	
Index ranges	−14<=h<=14, −13<=k<=13, −13<=l<=13	
Reflections collected	2713	
Absorption correction	multi-scan	
Max. and min. transmission	0.7200 and 0.6080	
Structure solution technique	direct methods	
Structure solution program	*SHELXT*	
Refinement method	Full-matrix least-squares on F^2^	
Refinement program	*SHELXL*	
Function minimized	Σ w(F_o_^2^ − F_c_^2^)^2^	
Data/restraints/parameters	2713/0/181	
Goodness-of-fit on F^2^	1.078	
Δ/σ_max_	0.001	
Final R indices	2680 data; I>2σ(I)	R1 = 0.0291, wR2 = 0.0763
	all data	R1 = 0.0293, wR2 = 0.0764
Weighting scheme	w = 1/[σ^2^(F_o_^2^) +(0.0387P)^2^ + 0.5487P] where P = (F_o_^2^ + 2F_c_^2^)/3	
Largest diff. peak and hole	0.242 and -0.291 eÅ^−3^	
R.M.S. deviation from the mean	0.039 eÅ^−3^	

## Supplementary Materials

The following supporting information can be downloaded at: https://www.mdpi.com/article/10.3390/molecules28073166/s1, Figure S1: 1H NMR spectrum of 3; Figure S2: APT 13C NMR spectrum of 3; Figure S3: 13C NMR spectrum of 4; Figure S4: APT 13C NMR spectrum of 4; Table S1: Physicochemical Properties of 3 and 4; Table S2: Lipophilicity of 3 and 4; Table S3: Water Solubility of 3 and 4; Table S4: Pharma-cokinetics Properties of 3 and 4; Table S5: Druglikeness Properties of 3 and 4; Table S6: Medicinal Properties of 3 and 4; CIF file.
