# Supplementary material for: Synthesis, X-ray Crystal Structure, Anticancer, Hirshfeld Surface Analysis, DFT, TD-DFT, ADMET, and Molecular Docking of 3-Phenyl-1,2,4-triazolo[3,4-h]-13,4-thiaza-11-crown-4
Source: Molecules. 2023 Apr 2;28(7):3166. doi: 10.3390/molecules28073166 (PMC10096472; doi:10.3390/molecules28073166)
Supplement: Supplementary file 1 [file molecules-28-03166-s001.zip › molecules-2314493-supplementary.pdf]

Synthesis, X-ray crystal structure, Anticancer, Hirshfeld Surface Analysis, DFT, TD-DFT, ADMET, and Molecular docking of 3-phenyl-1,2,4-triazolo[3,4-h]-13,4-thiaza-11-crown-4

Fatima Lazrak [1,*], Sanae Lahmidi [2], El Hassane Anouar [3,*], Mohammed M. Alanazi [4], Ashwag S. Alanazi [5], El Mokhtar Essassi [2] and Joel T. Mague [6]

[1] Laboratory of Medicinal Chemistry, Drug Sciences Research Center, Faculty of Medicine and Pharmacy, Mohammed V University in Rabat, Rabat 10106, Morocco
[2] Laboratory of Heterocyclic Organic Chemistry, Department of Chemistry, Faculty of Sciences, Mohammed V University in Rabat, Rabat 10106, Morocco; lahmidi_sanae@yahoo.fr (S.L.); emessassi@yahoo.fr (E.M.E.)
[3] Department of Chemistry, College of Science and Humanities in Al-Kharj, Prince Sattam bin Abdulaziz University, Al-Kharj 11942, Saudi Arabia
[4] Department of Pharmaceutical Chemistry, College of Pharmacy, King Saud University, Riyadh 11451, Saudi Arabia; mmalanazi@ksu.edu.sa
[5] Department of Pharmaceutical Sciences, College of Pharmacy, Princess Nourah bint Abdulrahman University, Riyadh 11671, Saudi Arabia; asalanzi@pnu.edu.sa
[6] Department of Chemistry, Tulane University, New Orleans, LA 70118, USA
* Correspondence: f.lazrak@um5r.ac.ma (F.L.); anouarelhassane@yahoo.fr (E.H.A.)

Figure S1 ^{1}H NMR spectrum spectrum of **3**.

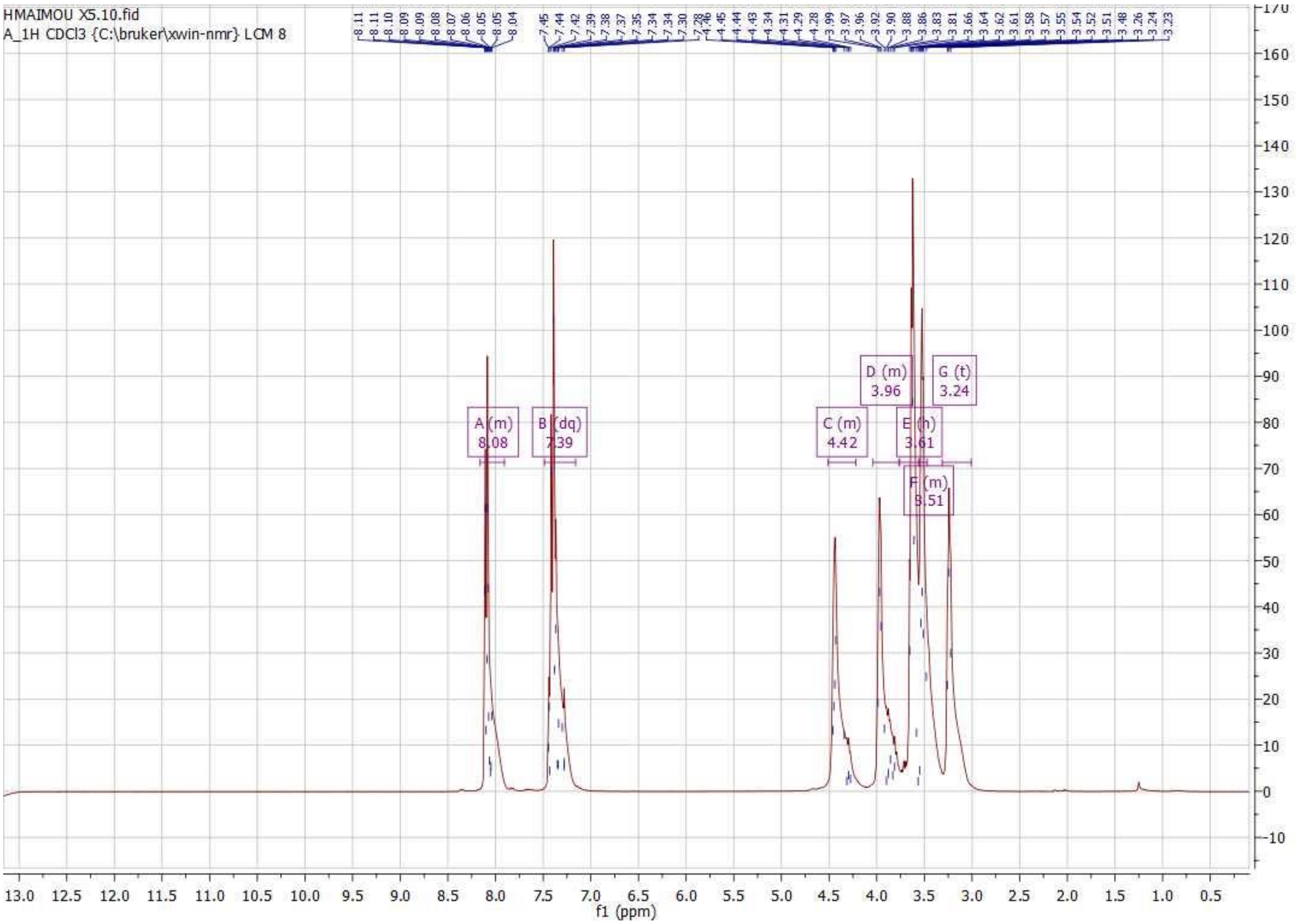

Figure S2 APT ^{13}C spectrum of **3**.

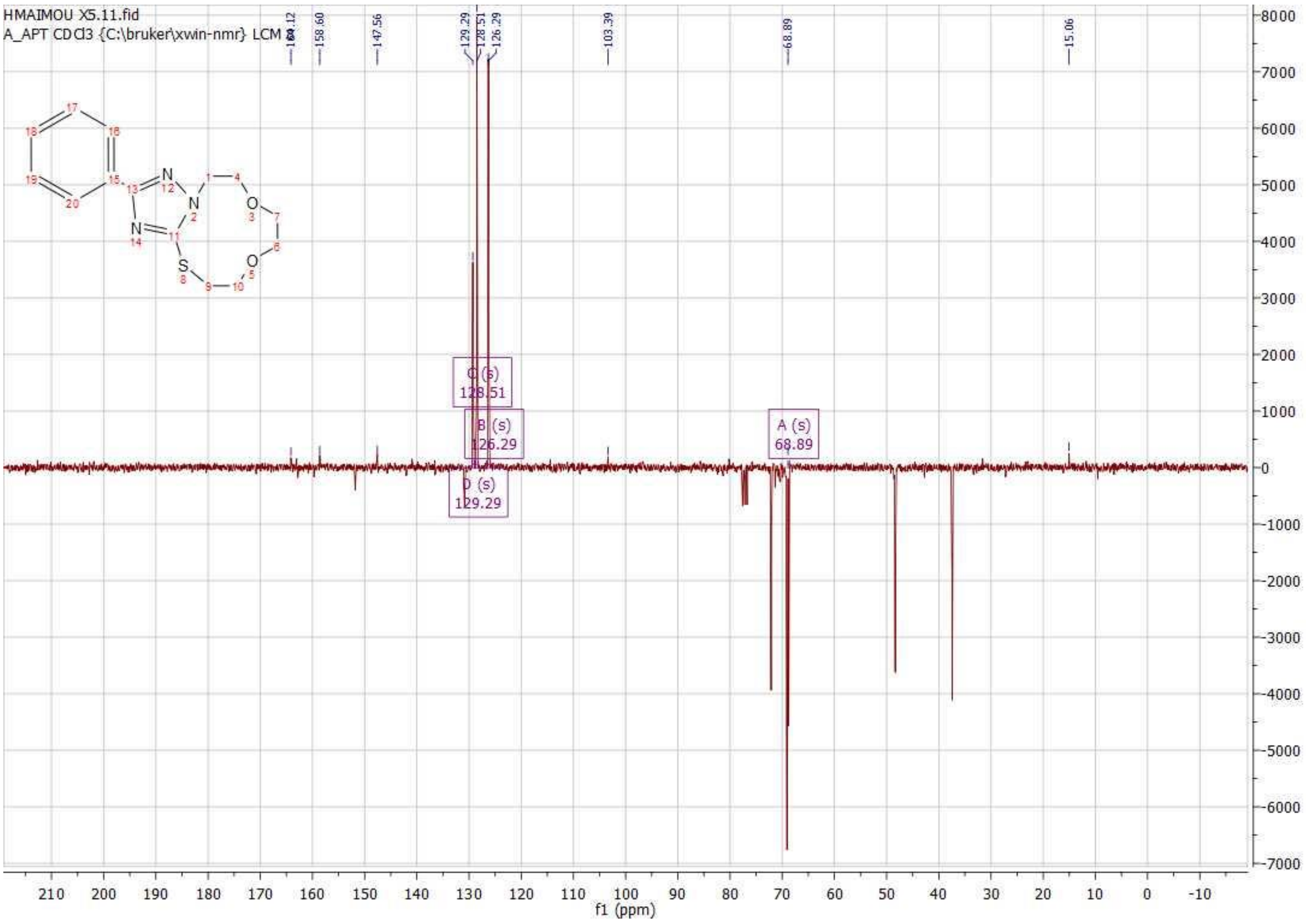

Figure S3 ^{13}C NMR spectrum of **4**.

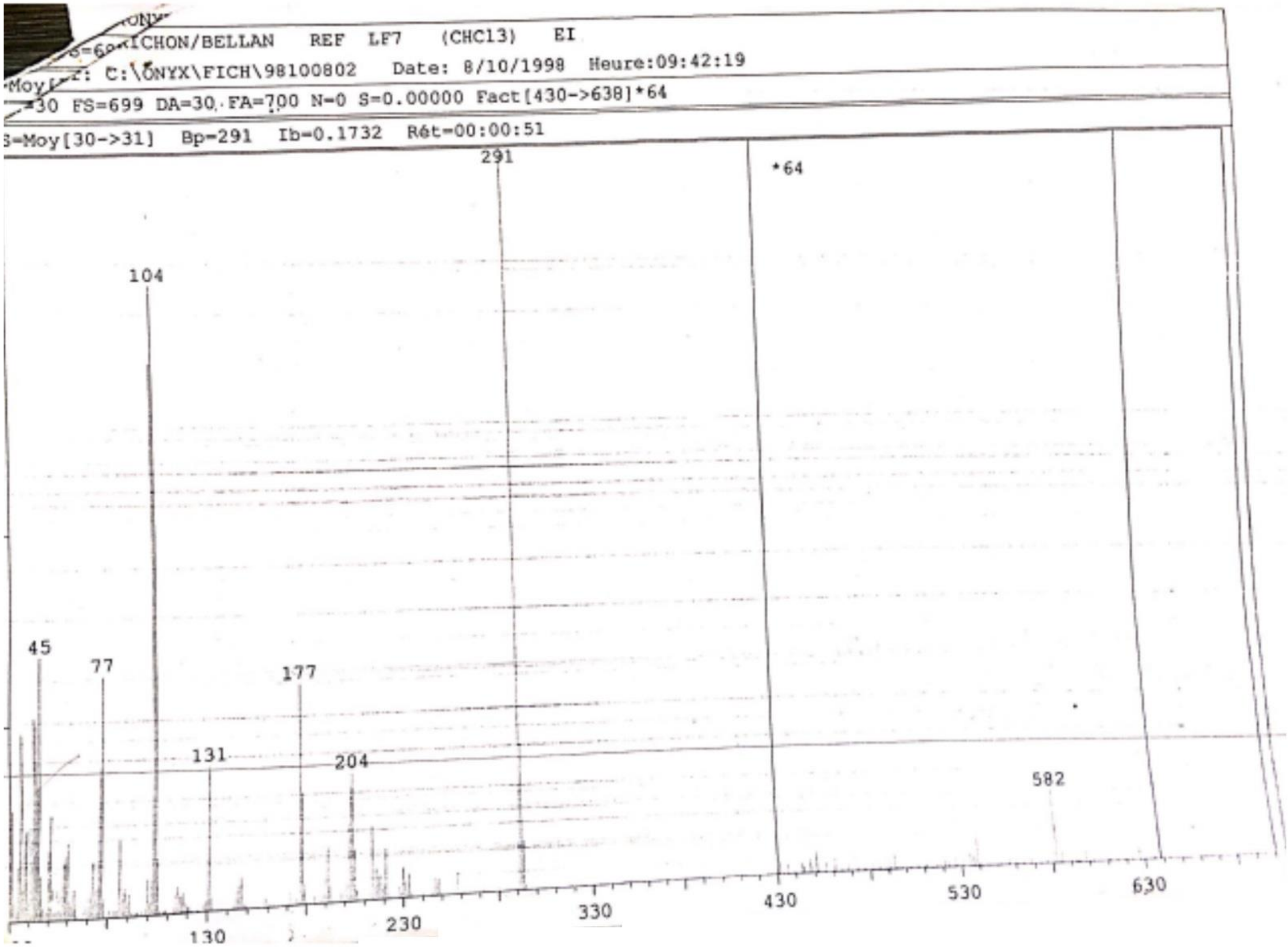

Figure S4 APT ^{13}C NMR spectrum of **4**.

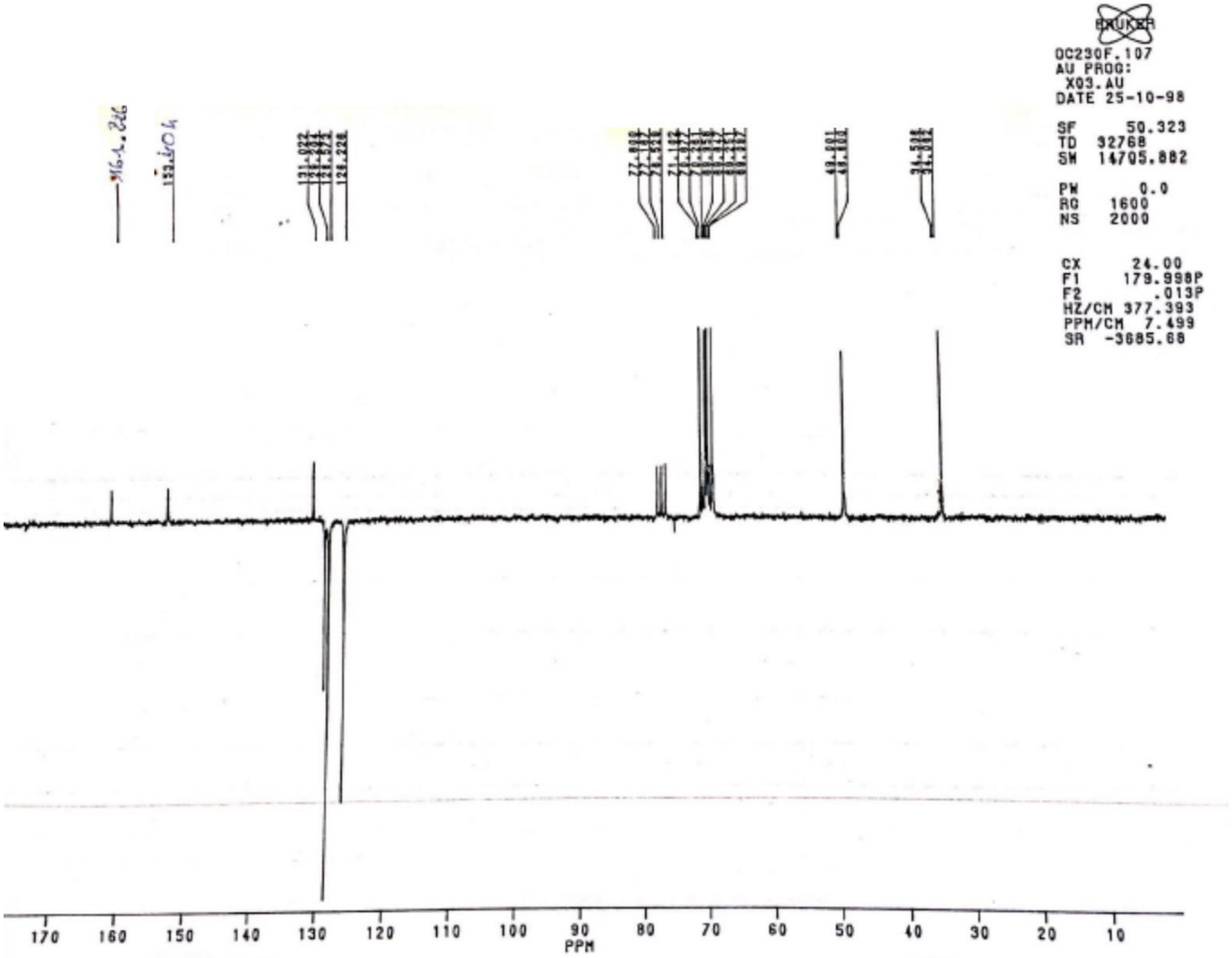

Table S1 Physicochemical Properties of the synthesized compounds.

Molecule	Formula	MW	#Heavy atoms	#Aromatic heavy atoms	Fraction Csp3	#Rotatable bonds	#H-bond acceptors	#H-bond donors	MR	TPSA
**3**	C14H17N3O2S	291.37	20	11	0.43	1	4	0	77.72	74.47
**4**	C28H34N6O4S2	582.74	40	22	0.43	2	8	0	155.45	148.94

Table S2 Lipophilicity of the synthesized compounds.

Molecule	iLOGP	XLOGP3	WLOGP	MLOGP	Silicos-IT Log P	Consensus Log P
**3**	2.8	1.92	2.08	1.66	2.14	2.12
**4**	4.5	3.85	4.17	2.58	2.78	3.58

Table S3 Water Solubility of the synthesized compounds.

Molecule	ESOL Log S	ESOL Solubility (mg/ml)	ESOL Solubility (mol/l)	ESOL Class	Ali Log S	Ali Solubility (mg/ml)	Ali Solubility (mol/l)	Ali Class	Silicos-IT LogSw	Silicos-IT Solubility (mg/ml)	Silicos-IT Solubility (mol/l)	Silicos-IT class
**3**	-3.2	1.85E-01	6.35E-04	Soluble	-3.11	2.28E-01	7.81E-04	Soluble	-3.81	4.55E-02	1.56E-04	Soluble
**4**	-6.15	4.09E-04	7.02E-07	Poorly soluble	-6.67	1.23E-04	2.12E-07	Poorly soluble	-7.47	1.96E-05	3.36E-08	Poorly soluble

Table S4 Pharmacokinetics Properties of the synthesized compounds.

Molecule	GI absorption	BBB permeant	Pgp substrate	CYP1A2 inhibitor	CYP2C19 inhibitor	CYP2C9 inhibitor	CYP2D6 inhibitor	CYP3A4 inhibitor	log Kp (cm/s)
**3**	High	Yes	Yes	Yes	No	No	No	No	-6.71
**4**	Low	No	Yes	No	No	Yes	No	No	-7.12

Table S5 Druglikeness Properties of the synthesized compounds..

Molecule	Lipinski #violations	Ghose #violations	Veber #violations	Egan #violations	Muegge #violations	Bioavailability Score
**3**	0	0	0	0	0	0.55
**4**	1	3	1	1	0	0.55

Table S6 Medicinal Properties of the synthesized compounds.

Molecule	PAINS #alerts	Brenk #alerts	Lead likeness #violations	Synthetic Accessibility
**3**	0	0	0	3.75
**4**	0	0	2	5.16

CIF File of 3

data_global

_audit_creation_method 'APEX4 v2021.10-0'

_publ_requested_journal ?

_publ_contact_author_name ?

_publ_contact_author_address

;

;

_publ_contact_author_email ?

_publ_contact_author_fax ?

_publ_contact_author_phone ?

_publ_section_title

;

Crystal structure of 2-phenyl-5,6,8,9,11,12-hexahydro-[1,2,4]triazolo[5,1-

<i>h</i>][1,4]dioxa[7]thia[9]azacycloundecine

;

_publ_section_abstract

;

The title molecule, C~14~H~17~N~3~O~2~S, adopts a zig-zag conformation and

a puckering analysis of the 11-membered ring was performed. In the crystal,

C---H...\p(ring) and weak S...O interactions form chains of molecules

extending along the <i>c</i>-axis direction which pack with normal van der

Waals contacts.

;

_publ_section_keywords

;

Crystal structure; C---H...\p(ring) interaction; S...O interaction;

triazole; dioxathiaazaundecine

;

_publ_section_references

;

Brandenburg, K. & Putz, H. (2012). <i>DIAMOND</i>,

Crystal Impact GbR, Bonn, Germany.

Bruker (2021). <i>APEX4</i>, <i>SAINT</i> & <i>SHELXTL</i>,
Bruker AXS LLC, Madison, WI.

Cremer, D. & Pople, J. A. (1975). <i>J. Am. Chem. Soc.</i> 97,
1354--1358.

Sheldrick, G, M. (2008). <i>CELL_NOW</i>, University
of G\"ottingen, G\"ottingen, Germany.

Sheldrick, G, M. (2009). <i>TWINABS</i>, University
of G\"ottingen, G\"ottingen, Germany.

Sheldrick, G. M. (2015<i>a</i>). <i>SHELXT</i>. <i>Acta Cryst.</i>
A71, 3-8.

Sheldrick, G. M. (2015<i>b</i>). <i>SHELXL-2018/1</i>.
<i>Acta Cryst.</i> C71, 3-8.
;

_publ_manuscript_text
;

The fused ring system adopts a zig-zag conformation (Fig. 1) and a puckering analysis (Cremer & Pople, 1975) of the 11-membered portion yielded the parameters Q(2), Q(3), Q(4) and Q(5) having values, respectively, of 0.1379(13), 1.2141(12), 0.9021(12) and 0.4116(13) \%A and the parameters \f(2), \f(3), \f(4) and \f(5) having values, respectively, of 275.7(5), 25.85(6), 349.44(8) and 99.44(16)\%. The total puckering amplitude is 1.5736(11) \%A. The dihedral angle between the mean planes of the C9...C14 and C1/N1/C2/N2/N3 rings is 21.77(7)\%. All bond distances and interbond angles appear as expected for the formulation given. In the crystal, C5---H5A...Cg1 interactions (Table 2) form chains of molecules extending along the <i>c</i>-axis direction (Fig. 2). Accompanying these are S1...O1^i^ (symmetry code: (i) <i>x</i>, -<i>y</i>+1/2, <i>z</i>-1/2) contacts which, at 3.1480(10) \%A are 0.17 \%A less than the sum of the respective van der Waals radii suggesting an attractive interaction reinforcing the chain formation. The chains pack with normal van der Waals contacts (Fig. 3).

;

_publ_section_exptl_refinement

;

Crystal, data collection and refinement details are presented in Table 1.

Hydrogen atoms were included as riding contributions in idealized positions

with isotropic displacement parameters tied to those of the attached atoms.

;

_publ_section_exptl_prep

;

;

_publ_section_figure_captions

;

The title molecule with labeling scheme and 50% probability ellipsoids.

A portion of one chain viewed along the <i>a</i>-axis direction with

C---H...\p(ring) interactions depicted by green dashed lines. The short

S...O contacts are depicted by orange dashed lines and non-interacting

hydrogen atoms are omitted for clarity.

Packing seen along the <i>c</i>-axis direction giving end views of several

chains. Intermolecular interactions aredepicted as in Fig. 2 and

non-interacting hydrogen atoms omitted for clarity.

;

_publ_section_acknowledgements
;
The support of NSF-MRI Grant #1228232 for the purchase of
the diffractometer and Tulane University for support of the
Tulane Crystallography Laboratory are gratefully acknowledged.
;

data_jtm1633_a

_audit_creation_method 'SHELXL-2019/1'
_shelx_SHELXL_version_number '2019/1'
_chemical_name_systematic
;
2-phenyl-5,6,8,9,11,12-hexahydro-[1,2,4]triazolo[5,1-
<i>h</i>][1,4]dioxa[7]thia[9]azacycloundecine
;
_chemical_name_common ?
_chemical_melting_point ?

_chemical_formula_moiety 'C14 H17 N3 O2 S'

_chemical_formula_sum

'C14 H17 N3 O2 S'

_chemical_formula_weight 291.36

loop_

_atom_type_symbol

_atom_type_description

_atom_type_scat_dispersion_real

_atom_type_scat_dispersion_imag

_atom_type_scat_source

'C' 'C' 0.0181 0.0091

'International Tables Vol C Tables 4.2.6.8 and 6.1.1.4'

'H' 'H' 0.0000 0.0000

'International Tables Vol C Tables 4.2.6.8 and 6.1.1.4'

'N' 'N' 0.0311 0.0180

'International Tables Vol C Tables 4.2.6.8 and 6.1.1.4'

'O' 'O' 0.0492 0.0322

'International Tables Vol C Tables 4.2.6.8 and 6.1.1.4'

'S' 'S' 0.3331 0.5567

'International Tables Vol C Tables 4.2.6.8 and 6.1.1.4'

```
_space_group_crystal_system      monoclinic
_space_group_IT_number           14
_space_group_name_H-M_alt        'P 21/c'
_space_group_name_Hall           '-P 2ybc'

_shelx_space_group_comment
;
The symmetry employed for this shelxl refinement is uniquely defined
by the following loop, which should always be used as a source of
symmetry information in preference to the above space-group names.
They are only intended as comments.
;

loop_
 _space_group_symop_operation_xyz
 'x, y, z'
 '-x, y+1/2, -z+1/2'
 '-x, -y, -z'
 'x, -y-1/2, z-1/2'
```

_cell_length_a 12.0933(5)

_cell_length_b 10.8160(4)

_cell_length_c 10.7940(4)

_cell_angle_alpha 90

_cell_angle_beta 102.708(1)

_cell_angle_gamma 90

_cell_volume 1377.28(9)

_cell_formula_units_Z 4

_cell_measurement_temperature 125(2)

_cell_measurement_reflns_used 9846

_cell_measurement_theta_min 3.747

_cell_measurement_theta_max 72.245

_exptl_crystal_description block

_exptl_crystal_colour colourless

_exptl_crystal_density_meas ?

_exptl_crystal_density_method 'not measured'

_exptl_crystal_density_diffrn 1.405

_exptl_crystal_F_000 616

_exptl_transmission_factor_min 0.785

_exptl_transmission_factor_max 0.864

_exptl_crystal_size_max 0.258

_exptl_crystal_size_mid 0.226

_exptl_crystal_size_min 0.164

_exptl_absorpt_coefficient_mu 2.139

_shelx_estimated_absorpt_T_min 0.608

_shelx_estimated_absorpt_T_max 0.720

_exptl_absorpt_correction_type multi-scan

_exptl_absorpt_process_details

'<i>TWINABS</i> (Sheldrick, 2009'

_exptl_absorpt_correction_T_min 0.61

_exptl_absorpt_correction_T_max 0.72

_exptl_special_details

;

The diffraction data were obtained from 14 sets of frames,

each of width 0.5\% in \w or \f, collected with scan parameters

determined by the "strategy" routine in <i>APEX4</i>. The scan

time was \q-dependent ranging from 5 to 10 sec/frame. Analysis of

1057 reflections having I/\s(I) > 20 and chosen from the full data set

with <i>CELL_NOW</i> (Sheldrick, 2008) showed the crystal to belong

to the monoclinic system and to be twinned by a 180\% rotation about

the <i>b</i> axis. The raw data were processed using the multi-component

version of <i>SAINT</i> under control of the two-component orientation

file generated by <i>CELL_NOW</i>.

;

_diffrn_ambient_temperature 125(2)

_diffrn_radiation_wavelength 1.54178

_diffrn_radiation_type CuK\a

_diffrn_source

'INCOATEC I\mS micro----focus source'

_diffrn_source_type ?

_diffrn_source_power 0.05

_diffrn_source_current 1.0

_diffrn_source_voltage 50

_diffrn_radiation_monochromator mirror

_diffrn_measurement_device

'Bruker D8 VENTURE PHOTON 3 CPAD'

_diffrn_measurement_device_type

'Bruker D8 VENTURE PHOTON 3 CPAD'

_diffrn_measurement_method '\f and \w scans'

_diffrn_detector_area_resol_mean 7.3910

_diffrn_reflns_number 51527

_diffrn_reflns_av_unetI/netI 0.0147

_diffrn_reflns_av_R_equivalents 0.0313

_diffrn_reflns_limit_h_min -14

_diffrn_reflns_limit_h_max 14

_diffrn_reflns_limit_k_min -13

_diffrn_reflns_limit_k_max 13

_diffrn_reflns_limit_l_min -13

_diffrn_reflns_limit_l_max 13

_diffrn_reflns_theta_min 3.747

_diffrn_reflns_theta_max 72.333

_diffrn_reflns_theta_full 72.300

_diffrn_measured_fraction_theta_max 0.998

_diffrn_measured_fraction_theta_full 0.998

_diffrn_reflns_Laue_measured_fraction_max 0.998

_diffrn_reflns_Laue_measured_fraction_full 0.998

_diffrn_reflns_point_group_measured_fraction_max 0.998

_diffrn_reflns_point_group_measured_fraction_full 0.998

_reflns_number_total 2713

_reflns_number_gt 2680

_reflns_threshold_expression 'I > 2\s(I)'

_reflns_Friedel_coverage 0.000

_reflns_Friedel_fraction_max .

_reflns_Friedel_fraction_full .

_reflns_special_details

;

Reflections were merged by SHELXL according to the crystal

class for the calculation of statistics and refinement.

_reflns_Friedel_fraction is defined as the number of unique

Friedel pairs measured divided by the number that would be

possible theoretically, ignoring centric projections and

systematic absences.

;

_computing_data_collection '<i>APEX4</i> (Bruker, 2021)'

_computing_cell_refinement '<i>SAINT</i> (Bruker, 2021)'

_computing_data_reduction '<i>SAINT</i> (Bruker, 2021)'

_computing_structure_solution

'<i>SHELXT</i> (Sheldrick, 2015<i>a</i>)'

_computing_structure_refinement

'<i>SHELXL-2018/1</i> (Sheldrick, 2015<i>b</i>)'

_computing_molecular_graphics

'<i>DIAMOND</i> (Brandenburg & Putz, 2012)'

_computing_publication_material '<i>SHELXTL</i> (Bruker, 2021)'

_refine_special_details

;

Refinement of F^2^ against ALL reflections. The weighted R-factor wR and goodness of fit S are based on F^2^, conventional R-factors R are based on F, with F set to zero for negative F^2^. The threshold expression of F^2^ > 2sigma(F^2^) is used only for calculating R-factors(gt) etc. and is not relevant to the choice of reflections for refinement. R-factors based on F^2^ are statistically about twice as large as those based on F, and R-factors based on ALL data will be even larger. H-atoms attached to carbon were placed in calculated positions (C---H = 0.95 - 0.99 \%A). All were included as riding contributions with isotropic displacement parameters 1.2 - 1.5 times those of the attached atoms. Trial refinements with the

single-component reflection file extracted from the twinned data with

<i>TWINABS</i> and the complete twinned data set showed the former to be

superior as judged by lower values of R1, wR2, the su's on the derived

parameters and residual peaks in the final difference map.

;

_refine_ls_structure_factor_coef Fsqd

_refine_ls_matrix_type   full

_refine_ls_weighting_scheme   calc

_refine_ls_weighting_details

'w=1/[\s^2^(Fo^2^)+(0.0387P)^2^+0.5487P] where P=(Fo^2^+2Fc^2^)/3'

_atom_sites_solution_primary   dual

_atom_sites_solution_secondary   difmap

_atom_sites_solution_hydrogens   geom

_refine_ls_hydrogen_treatment   constr

_refine_ls_extinction_method   none

_refine_ls_extinction_coef   .

_refine_ls_number_reflns   2713

_refine_ls_number_parameters   181

_refine_ls_number_restraints   0

_refine_ls_R_factor_all   0.0293

_refine_ls_R_factor_gt 0.0291

_refine_ls_wR_factor_ref 0.0764

_refine_ls_wR_factor_gt 0.0763

_refine_ls_goodness_of_fit_ref 1.078

_refine_ls_restrained_S_all 1.078

_refine_ls_shift/su_max 0.001

_refine_ls_shift/su_mean 0.000

loop_

_atom_site_label

_atom_site_type_symbol

_atom_site_fract_x

_atom_site_fract_y

_atom_site_fract_z

_atom_site_U_iso_or_equiv

_atom_site_adp_type

_atom_site_occupancy

_atom_site_site_symmetry_order

_atom_site_calc_flag

_atom_site_refinement_flags_posn

_atom_site_refinement_flags_adp

_atom_site_refinement_flags_occupancy

_atom_site_disorder_assembly

_atom_site_disorder_group

S1 S 0.74149(2) 0.16497(3) 0.38463(3) 0.02297(10) Uani 1 1 d . . . . .

O1 O 0.55964(9) 0.18328(8) 0.69003(9) 0.0321(2) Uani 1 1 d . . . . .

O2 O 0.69469(8) -0.00715(9) 0.59823(8) 0.0321(2) Uani 1 1 d . . . . .

N1 N 0.81258(8) 0.38241(9) 0.51096(9) 0.0213(2) Uani 1 1 d . . . . .

N2 N 0.63351(8) 0.33784(9) 0.50058(9) 0.0204(2) Uani 1 1 d . . . . .

N3 N 0.65074(8) 0.44656(9) 0.56512(9) 0.0214(2) Uani 1 1 d . . . . .

C1 C 0.76015(10) 0.46861(10) 0.57053(11) 0.0201(2) Uani 1 1 d . . . . .

C2 C 0.73018(10) 0.30130(11) 0.46889(11) 0.0204(2) Uani 1 1 d . . . . .

C3 C 0.83991(10) 0.08220(11) 0.50949(12) 0.0254(3) Uani 1 1 d . . . . .

H3A H 0.856361 0.000423 0.476490 0.031 Uiso 1 1 calc R U . . .

H3B H 0.912041 0.128817 0.531208 0.031 Uiso 1 1 calc R U . . .

C4 C 0.79610(11) 0.06342(12) 0.62884(12) 0.0255(3) Uani 1 1 d . . . . .

H4A H 0.853488 0.019433 0.693332 0.031 Uiso 1 1 calc R U . . .

H4B H 0.780868 0.144456 0.664350 0.031 Uiso 1 1 calc R U . . .

C5 C 0.64020(11) -0.02004(13) 0.70205(12) 0.0296(3) Uani 1 1 d . . . . .

H5A H 0.692358 0.007705 0.781166 0.035 Uiso 1 1 calc R U . . .

H5B H 0.622411 -0.108287 0.712300 0.035 Uiso 1 1 calc R U . . .

C6 C 0.53267(11) 0.05451(12) 0.68141(12) 0.0284(3) Uani 1 1 d . . . . .

H6A H 0.484810 0.035819 0.596635 0.034 Uiso 1 1 calc R U . . .

H6B H 0.489564 0.032364 0.746284 0.034 Uiso 1 1 calc R U . . .

C7 C 0.48501(11) 0.25949(12) 0.60241(13) 0.0282(3) Uani 1 1 d . . . . .

H7A H 0.479823 0.341494 0.641477 0.034 Uiso 1 1 calc R U . . .

H7B H 0.408372 0.222187 0.584012 0.034 Uiso 1 1 calc R U . . .

C8 C 0.52338(10) 0.27629(11) 0.47918(12) 0.0241(3) Uani 1 1 d . . . . .

H8A H 0.528680 0.194496 0.439728 0.029 Uiso 1 1 calc R U . . .

H8B H 0.466619 0.326155 0.419732 0.029 Uiso 1 1 calc R U . . .

C9 C 0.81903(10) 0.57545(10) 0.63959(11) 0.0207(2) Uani 1 1 d . . . . .

C10 C 0.77543(10) 0.63338(12) 0.73446(12) 0.0244(2) Uani 1 1 d . . . . .

H10 H 0.705959 0.605586 0.752064 0.029 Uiso 1 1 calc R U . . .

C11 C 0.83305(11) 0.73120(12) 0.80300(12) 0.0284(3) Uani 1 1 d . . . . .

H11 H 0.803193 0.769643 0.867730 0.034 Uiso 1 1 calc R U . . .

C12 C 0.93425(11) 0.77313(11) 0.77728(13) 0.0288(3) Uani 1 1 d . . . . .

H12 H 0.973681 0.840024 0.824350 0.035 Uiso 1 1 calc R U . . .

C13 C 0.97746(11) 0.71685(12) 0.68254(12) 0.0279(3) Uani 1 1 d . . . . .

H13 H 1.046190 0.746022 0.664127 0.033 Uiso 1 1 calc R U . . .

C14 C 0.92073(10) 0.61795(11) 0.61436(12) 0.0243(3) Uani 1 1 d . . . . .

H14 H 0.951351 0.579200 0.550364 0.029 Uiso 1 1 calc R U . . .

loop_

_atom_site_aniso_label

_atom_site_aniso_U_11

_atom_site_aniso_U_22

_atom_site_aniso_U_33

_atom_site_aniso_U_23

_atom_site_aniso_U_13

_atom_site_aniso_U_12

S1 0.02557(17) 0.02044(16) 0.02312(17) -0.00340(10) 0.00585(12) -0.00133(10)

O1 0.0457(6) 0.0243(5) 0.0245(5) -0.0003(4) 0.0039(4) -0.0115(4)

O2 0.0344(5) 0.0379(5) 0.0251(4) -0.0061(4) 0.0088(4) -0.0133(4)

N1 0.0230(5) 0.0192(5) 0.0218(5) 0.0006(4) 0.0049(4) -0.0001(4)

N2 0.0216(5) 0.0184(5) 0.0210(5) -0.0008(4) 0.0039(4) -0.0018(4)

N3 0.0236(5) 0.0181(5) 0.0218(5) -0.0007(4) 0.0038(4) -0.0006(4)

C1 0.0224(5) 0.0181(5) 0.0192(5) 0.0031(4) 0.0033(4) 0.0008(4)

C2 0.0230(6) 0.0192(5) 0.0191(5) 0.0021(4) 0.0049(4) 0.0005(4)

C3 0.0230(6) 0.0215(6) 0.0316(6) -0.0022(5) 0.0055(5) 0.0018(5)

C4 0.0258(6) 0.0220(6) 0.0269(6) -0.0015(5) 0.0017(5) -0.0016(5)

C5 0.0342(7) 0.0315(7) 0.0229(6) 0.0002(5) 0.0061(5) -0.0066(5)

C6 0.0357(7) 0.0235(6) 0.0258(6) -0.0001(5) 0.0068(5) -0.0086(5)

C7 0.0292(6) 0.0220(6) 0.0371(7) -0.0040(5) 0.0154(5) -0.0016(5)

C8 0.0206(6) 0.0227(6) 0.0281(6) 0.0018(5) 0.0034(5) -0.0025(4)

C9 0.0212(5) 0.0176(5) 0.0216(5) 0.0033(4) 0.0009(4) 0.0015(4)

C10 0.0216(6) 0.0240(6) 0.0266(6) -0.0008(5) 0.0035(5) -0.0001(5)

C11 0.0289(6) 0.0256(6) 0.0290(6) -0.0049(5) 0.0024(5) 0.0024(5)

C12 0.0291(6) 0.0201(6) 0.0329(7) -0.0012(5) -0.0023(5) -0.0020(5)

C13 0.0238(6) 0.0244(6) 0.0339(7) 0.0055(5) 0.0028(5) -0.0037(5)

C14 0.0252(6) 0.0218(6) 0.0257(6) 0.0029(5) 0.0052(5) 0.0006(5)

_geom_special_details

;

All esds (except the esd in the dihedral angle between two l.s. planes)

are estimated using the full covariance matrix. The cell esds are taken

into account individually in the estimation of esds in distances, angles

and torsion angles; correlations between esds in cell parameters are only

used when they are defined by crystal symmetry. An approximate (isotropic)

treatment of cell esds is used for estimating esds involving l.s. planes.

;

loop_

_geom_bond_atom_site_label_1

_geom_bond_atom_site_label_2

_geom_bond_distance

_geom_bond_site_symmetry_2

_geom_bond_publ_flag

S1 C2 1.7535(12) . ?

S1 C3 1.8244(13) . ?

O1 C7 1.4188(17) . ?

O1 C6 1.4288(15) . ?

O2 C4 1.4204(15) . ?

O2 C5 1.4263(15) . ?

N1 C2 1.3303(15) . ?

N1 C1 1.3653(15) . ?

N2 C2 1.3476(15) . ?

N2 N3 1.3593(14) . ?

N2 C8 1.4611(15) . ?

N3 C1 1.3330(15) . ?

C1 C9 1.4713(16) . ?

C3 C4 1.5106(17) . ?

C3 H3A 0.9900 . ?

C3 H3B 0.9900 . ?

C4 H4A 0.9900 . ?

C4 H4B 0.9900 . ?

C5 C6 1.5045(19) . ?

C5 H5A 0.9900 . ?

C5 H5B 0.9900 . ?

C6 H6A 0.9900 . ?

C6 H6B 0.9900 . ?

C7 C8 1.5131(17) . ?

C7 H7A 0.9900 . ?

C7 H7B 0.9900 . ?

C8 H8A 0.9900 . ?

C8 H8B 0.9900 . ?

C9 C14 1.3947(17) . ?

C9 C10 1.3988(17) . ?

C10 C11 1.3877(18) . ?

C10 H10 0.9500 . ?

C11 C12 1.3892(19) . ?

C11 H11 0.9500 . ?

C12 C13 1.3872(19) . ?

C12 H12 0.9500 . ?

C13 C14 1.3909(18) . ?

C13 H13 0.9500 . ?

C14 H14 0.9500 . ?

```
loop_
 _geom_angle_atom_site_label_1
 _geom_angle_atom_site_label_2
 _geom_angle_atom_site_label_3
 _geom_angle
 _geom_angle_site_symmetry_1
 _geom_angle_site_symmetry_3
 _geom_angle_publ_flag
C2 S1 C3 98.17(6) . . ?
C7 O1 C6 114.85(10) . . ?
C4 O2 C5 113.22(10) . . ?
C2 N1 C1 102.73(10) . . ?
C2 N2 N3 109.88(9) . . ?
C2 N2 C8 129.62(10) . . ?
N3 N2 C8 120.46(10) . . ?
C1 N3 N2 102.47(9) . . ?
N3 C1 N1 114.62(10) . . ?
N3 C1 C9 121.86(10) . . ?
N1 C1 C9 123.49(10) . . ?
N1 C2 N2 110.27(10) . . ?
N1 C2 S1 126.41(9) . . ?
```

N2 C2 S1 123.32(9) . . ?

C4 C3 S1 113.69(8) . . ?

C4 C3 H3A 108.8 . . ?

S1 C3 H3A 108.8 . . ?

C4 C3 H3B 108.8 . . ?

S1 C3 H3B 108.8 . . ?

H3A C3 H3B 107.7 . . ?

O2 C4 C3 108.71(10) . . ?

O2 C4 H4A 109.9 . . ?

C3 C4 H4A 109.9 . . ?

O2 C4 H4B 109.9 . . ?

C3 C4 H4B 109.9 . . ?

H4A C4 H4B 108.3 . . ?

O2 C5 C6 111.40(11) . . ?

O2 C5 H5A 109.3 . . ?

C6 C5 H5A 109.3 . . ?

O2 C5 H5B 109.3 . . ?

C6 C5 H5B 109.3 . . ?

H5A C5 H5B 108.0 . . ?

O1 C6 C5 109.59(11) . . ?

O1 C6 H6A 109.8 . . ?

C5 C6 H6A 109.8 . . ?

O1 C6 H6B 109.8 . . ?

C5 C6 H6B 109.8 . . ?

H6A C6 H6B 108.2 . . ?

O1 C7 C8 112.50(10) . . ?

O1 C7 H7A 109.1 . . ?

C8 C7 H7A 109.1 . . ?

O1 C7 H7B 109.1 . . ?

C8 C7 H7B 109.1 . . ?

H7A C7 H7B 107.8 . . ?

N2 C8 C7 111.16(10) . . ?

N2 C8 H8A 109.4 . . ?

C7 C8 H8A 109.4 . . ?

N2 C8 H8B 109.4 . . ?

C7 C8 H8B 109.4 . . ?

H8A C8 H8B 108.0 . . ?

C14 C9 C10 119.07(11) . . ?

C14 C9 C1 120.61(11) . . ?

C10 C9 C1 120.29(10) . . ?

C11 C10 C9 120.39(12) . . ?

C11 C10 H10 119.8 . . ?

C9 C10 H10 119.8 . . ?

C10 C11 C12 120.23(12) . . ?

C10 C11 H11 119.9 . . ?

C12 C11 H11 119.9 . . ?

C13 C12 C11 119.69(12) . . ?

C13 C12 H12 120.2 . . ?

C11 C12 H12 120.2 . . ?

C12 C13 C14 120.37(12) . . ?

C12 C13 H13 119.8 . . ?

C14 C13 H13 119.8 . . ?

C13 C14 C9 120.24(12) . . ?

C13 C14 H14 119.9 . . ?

C9 C14 H14 119.9 . . ?

loop_

_geom_torsion_atom_site_label_1

_geom_torsion_atom_site_label_2

_geom_torsion_atom_site_label_3

_geom_torsion_atom_site_label_4

_geom_torsion

_geom_torsion_site_symmetry_1

_geom_torsion_site_symmetry_2

_geom_torsion_site_symmetry_3

_geom_torsion_site_symmetry_4

_geom_torsion_publ_flag

C2 N2 N3 C1 -1.05(12) . . . . ?

C8 N2 N3 C1 176.79(10) . . . . ?

N2 N3 C1 N1 1.50(13) . . . . ?

N2 N3 C1 C9 -176.43(10) . . . . ?

C2 N1 C1 N3 -1.34(13) . . . . ?

C2 N1 C1 C9 176.55(10) . . . . ?

C1 N1 C2 N2 0.58(12) . . . . ?

C1 N1 C2 S1 -179.15(9) . . . . ?

N3 N2 C2 N1 0.30(13) . . . . ?

C8 N2 C2 N1 -177.29(11) . . . . ?

N3 N2 C2 S1 -179.96(8) . . . . ?

C8 N2 C2 S1 2.46(17) . . . . ?

C3 S1 C2 N1 65.46(11) . . . . ?

C3 S1 C2 N2 -114.25(10) . . . . ?

C2 S1 C3 C4 58.89(10) . . . . ?

C5 O2 C4 C3 -174.25(10) . . . . ?

S1 C3 C4 O2 60.54(12) . . . . ?

C4 O2 C5 C6 108.86(12) . . . . ?

C7 O1 C6 C5 141.78(11) . . . . ?

O2 C5 C6 O1 -69.67(13) . . . . ?

C6 O1 C7 C8 -90.60(13) . . . . ?

C2 N2 C8 C7 124.69(13) . . . . ?

N3 N2 C8 C7 -52.68(14) . . . . ?

O1 C7 C8 N2 -62.12(13) . . . . ?

N3 C1 C9 C14 -160.95(11) . . . . ?

N1 C1 C9 C14 21.31(17) . . . . ?

N3 C1 C9 C10 21.07(17) . . . . ?

N1 C1 C9 C10 -156.66(11) . . . . ?

C14 C9 C10 C11 -0.44(17) . . . . ?

C1 C9 C10 C11 177.57(11) . . . . ?

C9 C10 C11 C12 0.50(19) . . . . ?

C10 C11 C12 C13 0.10(19) . . . . ?

C11 C12 C13 C14 -0.75(19) . . . . ?

C12 C13 C14 C9 0.81(18) . . . . ?

C10 C9 C14 C13 -0.21(17) . . . . ?

C1 C9 C14 C13 -178.21(11) . . . . ?

loop_

_geom_hbond_atom_site_label_D

_geom_hbond_atom_site_label_H

_geom_hbond_atom_site_label_A

_geom_hbond_distance_DH

_geom_hbond_distance_HA

_geom_hbond_distance_DA

_geom_hbond_angle_DHA

_geom_hbond_site_symmetry_A

_geom_hbond_publ_flag

C5 H5A Cg1 0.99 2.80 3.6778(14) 148 4_555 y

_iucr_geom_hbonds_special_details

;

Cg1 is the centroid of the triazole ring.

;

_refine_diff_density_max   0.242

_refine_diff_density_min   -0.291

_refine_diff_density_rms   0.039

_shelx_res_file

```
;

TITL jtm1633_a.res in P2(1)/c

    jtm1633_a.res

    created by SHELXL-2019/1 at 12:07:40 on 25-Oct-2022

REM Old TITL jtm1633 in P2(1)/c

REM SHELXT solution in P2(1)/c: R1 0.093, Rweak 0.002, Alpha 0.022

REM <I/s>   1.128 for  158 systematic absences,  Orientation as input

REM Formula found by SHELXT:  C14 N4 O S

CELL  1.54178  12.0933  10.8160  10.7940   90.000  102.708   90.000

ZERR    4.000   0.0005   0.0004   0.0004    0.000    0.001    0.000

LATT  1

SYMM -X, 1/2+Y, 1/2-Z

SFAC C H N O S

UNIT 56 68 12 8 4

TEMP -148

SIZE 0.164 0.226 0.258

L.S. 5

BOND $H

LIST 4

CONF

MPLA C9 > C14
```

```
MPLA C1 N1 C2 N2 N3

ACTA 144.6

FMAP 2

PLAN 5

WGHT    0.038700    0.548700

FVAR       0.93127

S1    5    0.741494    0.164968    0.384633    11.00000    0.02557    0.02044 =

        0.02312   -0.00340    0.00585   -0.00133

O1    4    0.559640    0.183282    0.690032    11.00000    0.04568    0.02433 =

        0.02452   -0.00033    0.00386   -0.01152

O2    4    0.694687   -0.007149    0.598233    11.00000    0.03438    0.03791 =

        0.02509   -0.00608    0.00884   -0.01330

N1    3    0.812584    0.382407    0.510957    11.00000    0.02302    0.01918 =

        0.02182    0.00057    0.00490   -0.00013

N2    3    0.633508    0.337840    0.500583    11.00000    0.02155    0.01844 =

        0.02095   -0.00081    0.00388   -0.00184

N3    3    0.650742    0.446563    0.565117    11.00000    0.02357    0.01814 =

        0.02179   -0.00075    0.00379   -0.00055

C1    1    0.760151    0.468605    0.570527    11.00000    0.02242    0.01809 =

        0.01924    0.00310    0.00333    0.00080

C2    1    0.730182    0.301301    0.468885    11.00000    0.02299    0.01919 =
```

```
       0.01906    0.00207    0.00487    0.00045
C3    1    0.839909    0.082204    0.509493    11.00000    0.02305    0.02149 =
       0.03157   -0.00218    0.00555    0.00180
AFIX  23
H3A   2    0.856361    0.000423    0.476490    11.00000   -1.20000
H3B   2    0.912041    0.128817    0.531208    11.00000   -1.20000
AFIX   0
C4    1    0.796098    0.063422    0.628839    11.00000    0.02579    0.02201 =
       0.02688   -0.00147    0.00165   -0.00163
AFIX  23
H4A   2    0.853488    0.019433    0.693332    11.00000   -1.20000
H4B   2    0.780868    0.144456    0.664350    11.00000   -1.20000
AFIX   0
C5    1    0.640201   -0.020036    0.702055    11.00000    0.03421    0.03151 =
       0.02291    0.00021    0.00609   -0.00658
AFIX  23
H5A   2    0.692358    0.007705    0.781166    11.00000   -1.20000
H5B   2    0.622411   -0.108287    0.712300    11.00000   -1.20000
AFIX   0
C6    1    0.532666    0.054513    0.681406    11.00000    0.03572    0.02351 =
       0.02584   -0.00010    0.00678   -0.00864
```

```
AFIX  23
H6A   2    0.484810    0.035819    0.596635    11.00000   -1.20000
H6B   2    0.489564    0.032364    0.746284    11.00000   -1.20000
AFIX   0
C7    1    0.485006    0.259486    0.602415    11.00000    0.02920    0.02196 =
       0.03705   -0.00399    0.01541   -0.00162
AFIX  23
H7A   2    0.479823    0.341494    0.641477    11.00000   -1.20000
H7B   2    0.408372    0.222187    0.584012    11.00000   -1.20000
AFIX   0
C8    1    0.523383    0.276289    0.479181    11.00000    0.02056    0.02272 =
       0.02807    0.00182    0.00337   -0.00249
AFIX  23
H8A   2    0.528680    0.194496    0.439728    11.00000   -1.20000
H8B   2    0.466619    0.326155    0.419732    11.00000   -1.20000
AFIX   0
C9    1    0.819035    0.575447    0.639595    11.00000    0.02123    0.01765 =
       0.02156    0.00330    0.00094    0.00149
C10   1    0.775428    0.633377    0.734457    11.00000    0.02164    0.02396 =
       0.02663   -0.00084    0.00346   -0.00011
AFIX  43
```

```
H10   2    0.705959    0.605586    0.752064    11.00000   -1.20000
AFIX   0
C11   1    0.833053    0.731204    0.802995    11.00000    0.02892    0.02559 =
       0.02898   -0.00487    0.00239    0.00243
AFIX  43
H11   2    0.803193    0.769643    0.867730    11.00000   -1.20000
AFIX   0
C12   1    0.934251    0.773126    0.777277    11.00000    0.02913    0.02015 =
       0.03287   -0.00119   -0.00235   -0.00199
AFIX  43
H12   2    0.973681    0.840024    0.824350    11.00000   -1.20000
AFIX   0
C13   1    0.977464    0.716848    0.682538    11.00000    0.02379    0.02439 =
       0.03390    0.00550    0.00284   -0.00370
AFIX  43
H13   2    1.046190    0.746022    0.664127    11.00000   -1.20000
AFIX   0
C14   1    0.920726    0.617947    0.614359    11.00000    0.02516    0.02183 =
       0.02565    0.00288    0.00521    0.00065
AFIX  43
H14   2    0.951351    0.579200    0.550364    11.00000   -1.20000
```

AFIX 0

HKLF 4

REM jtm1633_a.res in P2(1)/c

REM wR2 = 0.0764, GooF = S = 1.078, Restrained GooF = 1.078 for all data

REM R1 = 0.0291 for 2680 Fo > 4sig(Fo) and 0.0293 for all 2713 data

REM 181 parameters refined using 0 restraints

END

WGHT 0.0387 0.5487

REM Highest difference peak 0.242, deepest hole -0.291, 1-sigma level 0.039

Q1 1 0.8120 0.0685 0.5661 11.00000 0.05 0.24

Q2 1 0.7954 0.1286 0.4536 11.00000 0.05 0.23

Q3 1 0.5053 0.2619 0.5363 11.00000 0.05 0.23

Q4 1 0.7880 0.5195 0.5971 11.00000 0.05 0.23

Q5 1 0.8785 0.5785 0.6422 11.00000 0.05 0.23

```
;
_shelx_res_checksum   91653

_shelx_hkl_file
;
   0   0   1    0.30    0.20
   0   0   2  957.90   19.20
   0   0   3    1.20    0.30
   0   0   4  170.48    3.70
   0   0   6  131.89    3.40
   0   0   7    1.20    0.40
   0   0   8  536.25    6.20
   0   0   9    0.30    0.20
   0   0  10 1602.74   22.20
   0   0  11    0.10    0.20
   0   0  12   93.19    1.90
   0   0  13    0.10    0.10
   0  -1   0    0.40    0.10
   0  -1   1   72.59    1.50
   0  -1   2  263.57    5.00
   0  -1   3 4521.15   77.39
```

0	-1	4	1080.79	19.40
0	-1	5	27.00	1.00
0	-1	6	1121.79	14.60
0	-1	7	0.40	0.20
0	-1	8	44.60	0.80
0	-1	9	235.38	2.40
0	-1	10	498.35	5.00
0	-1	11	115.69	1.60
0	-1	12	40.70	0.80
0	-2	0	777.62	19.70
0	-2	1	2123.79	30.00
0	-2	2	401.36	6.70
0	-2	3	1637.14	25.70
0	-2	4	545.95	10.70
0	-2	5	63.39	2.20
0	-2	6	538.65	7.10
0	-2	7	575.54	5.50
0	-2	8	123.79	1.50
0	-2	9	26.60	0.70
0	-2	10	120.69	1.70
0	-2	11	31.20	0.70

0	-2	12	62.99	1.00
0	-3	0	0.20	0.20
0	-3	1	1187.08	14.00
0	-3	2	28.10	1.40
0	-3	3	1268.97	20.20
0	-3	4	1127.79	18.40
0	-3	5	2009.90	32.30
0	-3	6	377.06	6.60
0	-3	7	114.19	1.40
0	-3	8	798.12	6.50
0	-3	9	71.49	1.10
0	-3	10	57.69	1.30
0	-3	11	38.80	0.80
0	-3	12	17.20	0.60
0	-4	0	480.25	13.00
0	-4	1	167.68	2.80
0	-4	2	639.14	8.30
0	-4	3	404.26	8.10
0	-4	4	33.70	1.10
0	-4	5	1704.93	25.20
0	-4	6	247.78	4.00

0	-4	7	1775.32	14.70
0	-4	8	1494.55	12.90
0	-4	9	418.86	4.10
0	-4	10	6.10	0.60
0	-4	11	148.79	2.30
0	-4	12	0.20	0.10
0	-5	0	0.10	0.30
0	-5	1	187.48	5.00
0	-5	2	3837.52	50.79
0	-5	3	1922.71	23.00
0	-5	4	527.65	7.40
0	-5	5	477.05	8.10
0	-5	6	298.17	3.40
0	-5	7	16.70	0.60
0	-5	8	615.24	5.70
0	-5	9	194.18	2.80
0	-5	10	88.89	1.50
0	-5	11	2.70	0.30
0	-5	12	0.20	0.10
0	-6	0	394.76	11.80
0	-6	1	15.90	0.90

0	-6	2	8.00	0.70
0	-6	3	61.29	1.60
0	-6	4	291.57	4.60
0	-6	5	361.56	3.80
0	-6	6	132.89	1.70
0	-6	7	807.72	8.30
0	-6	8	71.79	1.30
0	-6	9	44.00	1.10
0	-6	10	211.08	3.70
0	-6	11	170.98	3.50
0	-7	0	0.20	0.30
0	-7	1	439.06	6.80
0	-7	2	42.00	1.10
0	-7	3	163.78	2.30
0	-7	4	58.79	1.10
0	-7	5	539.95	5.60
0	-7	6	248.08	3.60
0	-7	7	58.09	1.20
0	-7	8	49.50	1.30
0	-7	9	3.40	0.40
0	-7	10	477.95	9.70

0	-7	11	23.10	1.20
0	-8	0	116.19	2.30
0	-8	1	723.53	7.30
0	-8	2	32.20	0.80
0	-8	3	1249.68	11.10
0	-8	4	176.38	2.30
0	-8	5	47.90	1.10
0	-8	6	231.58	2.90
0	-8	7	9.90	0.70
0	-8	8	21.50	0.90
0	-8	9	0.70	0.20
0	-8	10	69.49	2.00
0	-9	0	0.20	0.20
0	-9	1	471.95	5.10
0	-9	2	17.30	0.70
0	-9	3	527.45	5.40
0	-9	4	140.29	2.00
0	-9	5	25.50	0.90
0	-9	6	0.30	0.20
0	-9	7	169.48	2.80
0	-9	8	61.89	1.90

0	-9	9	386.86	10.50
0	-10	0	263.97	4.00
0	-10	1	1.10	0.20
0	-10	2	1.40	0.20
0	-10	3	27.70	0.80
0	-10	4	222.38	3.00
0	-10	5	26.50	0.80
0	-10	6	112.09	1.90
0	-10	7	128.29	2.30
0	-10	8	16.10	0.90
0	-11	0	0.30	0.20
0	-11	1	461.75	4.60
0	-11	2	1.70	0.20
0	-11	3	0.80	0.20
0	-11	4	34.00	0.90
0	-11	5	4.60	0.50
0	-11	6	199.48	2.90
0	-11	7	48.30	1.30
0	-12	0	97.59	2.30
0	-12	1	36.00	0.80
0	-12	2	11.40	0.50

0	-12	3	5.30	0.40
0	-12	4	351.36	4.20
0	-12	5	28.70	0.90
0	-13	0	0.00	0.10
0	-13	1	183.08	2.70
0	-13	2	140.49	2.60
0	-13	3	22.60	0.80
1	0	-13	0.00	0.10
1	0	-12	95.99	2.00
1	0	-11	0.10	0.20
1	0	-10	29.70	1.10
1	0	-9	0.10	0.30
1	0	-8	218.28	3.40
1	0	-7	0.40	0.30
1	0	-6	255.47	5.60
1	0	-4	65.79	1.80
1	0	-3	0.70	0.20
1	0	-2	5864.71	144.79
1	0	-1	0.30	0.10
1	0	0	2001.60	39.80
1	0	1	0.30	0.10

1	0	2	330.67	7.10
1	0	3	0.10	0.20
1	0	4	17.20	1.30
1	0	5	0.20	0.50
1	0	6	444.36	9.00
1	0	7	0.10	0.20
1	0	8	223.78	3.30
1	0	9	0.20	0.20
1	0	10	649.54	8.00
1	0	11	0.20	0.20
1	0	12	298.07	5.00
1	-1	-13	5.40	0.40
1	-1	-12	96.89	1.40
1	-1	-11	140.79	2.00
1	-1	-10	756.02	6.70
1	-1	-9	34.40	0.90
1	-1	-8	742.63	6.60
1	-1	-7	18.60	0.90
1	-1	-6	955.80	11.80
1	-1	-5	72.39	1.60
1	-1	-4	185.48	4.10

1	-1	-2	2634.64	40.50
1	-1	-1	3.10	0.20
1	-1	1	429.16	7.80
1	-1	3	705.93	11.50
1	-1	4	657.43	9.20
1	-1	5	327.47	5.60
1	-1	6	8.70	0.80
1	-1	7	319.57	3.10
1	-1	8	276.27	2.80
1	-1	9	100.39	1.30
1	-1	10	2.30	0.20
1	-1	11	7.50	0.40
1	-1	12	0.40	0.10
1	-2	-13	277.17	4.30
1	-2	-12	25.50	0.60
1	-2	-11	130.59	1.80
1	-2	-10	97.99	1.50
1	-2	-9	746.83	6.80
1	-2	-8	1048.10	8.60
1	-2	-7	17.70	0.70
1	-2	-6	118.29	1.90

1	-2	-5	1967.60	34.90
1	-2	-4	794.92	14.70
1	-2	-3	2707.13	42.00
1	-2	-2	2032.60	31.50
1	-2	-1	393.86	4.50
1	-2	0	73.89	1.30
1	-2	1	61.09	1.20
1	-2	3	9999.00	347.17
1	-2	4	615.84	10.40
1	-2	5	1220.38	16.70
1	-2	6	23.90	0.90
1	-2	7	643.34	6.20
1	-2	8	38.90	0.80
1	-2	9	236.58	2.50
1	-2	10	114.79	1.80
1	-2	11	117.29	1.60
1	-2	12	40.80	0.80
1	-3	-12	95.99	1.40
1	-3	-11	791.42	8.00
1	-3	-10	20.80	0.70
1	-3	-9	57.79	1.00

1	-3	-8	44.20	0.80
1	-3	-7	1142.79	10.50
1	-3	-6	8.00	0.80
1	-3	-5	659.43	10.40
1	-3	-4	42.80	1.70
1	-3	-3	2155.98	33.70
1	-3	-2	584.54	8.10
1	-3	-1	4308.87	47.30
1	-3	0	1877.11	32.80
1	-3	1	2562.44	31.50
1	-3	2	1548.95	22.50
1	-3	3	84.29	2.40
1	-3	4	199.48	5.40
1	-3	5	441.16	8.20
1	-3	6	145.89	2.60
1	-3	7	2244.88	18.30
1	-3	8	20.50	0.60
1	-3	9	1.70	0.20
1	-3	10	167.18	2.00
1	-3	11	408.46	4.40
1	-3	12	0.10	0.10

1	-4	-12	12.90	0.60
1	-4	-11	6.20	0.40
1	-4	-10	6.80	0.50
1	-4	-9	3.80	0.30
1	-4	-8	26.30	0.70
1	-4	-7	770.52	6.80
1	-4	-6	1030.10	13.60
1	-4	-5	1952.40	26.50
1	-4	-4	69.39	1.60
1	-4	-3	1294.77	20.70
1	-4	-2	3093.99	34.20
1	-4	-1	88.69	1.90
1	-4	0	1186.38	24.80
1	-4	1	437.46	6.90
1	-4	2	146.19	2.10
1	-4	3	381.76	6.20
1	-4	4	1046.50	16.00
1	-4	5	462.65	9.60
1	-4	6	201.48	3.20
1	-4	7	421.06	3.90
1	-4	8	587.04	5.40

1	-4	9	95.19	1.40
1	-4	10	154.38	2.10
1	-4	11	26.90	0.70
1	-4	12	19.80	0.70
1	-5	-12	103.49	2.70
1	-5	-11	27.50	1.00
1	-5	-10	114.59	1.80
1	-5	-9	1.80	0.20
1	-5	-8	505.35	4.90
1	-5	-7	64.09	1.00
1	-5	-6	60.19	1.20
1	-5	-5	516.95	7.80
1	-5	-4	24.70	0.90
1	-5	-3	986.60	11.50
1	-5	-2	111.59	2.20
1	-5	-1	185.68	4.30
1	-5	0	1114.79	23.80
1	-5	1	656.83	12.80
1	-5	2	211.38	3.50
1	-5	3	362.76	4.40
1	-5	4	727.93	10.50

1	-5	5	0.80	0.20
1	-5	6	421.86	4.20
1	-5	7	420.26	4.00
1	-5	8	285.27	3.00
1	-5	9	45.30	0.90
1	-5	10	936.91	10.10
1	-5	11	1.00	0.20
1	-6	-11	4.10	0.50
1	-6	-10	129.49	2.80
1	-6	-9	0.00	0.20
1	-6	-8	33.80	0.90
1	-6	-7	513.05	5.60
1	-6	-6	1726.93	15.50
1	-6	-5	65.49	1.30
1	-6	-4	38.10	1.30
1	-6	-3	40.00	1.20
1	-6	-2	1241.88	20.40
1	-6	-1	680.83	11.80
1	-6	0	1396.96	25.60
1	-6	1	36.70	1.50
1	-6	2	246.78	4.90

1	-6	3	53.89	1.50
1	-6	4	481.85	6.10
1	-6	5	90.49	1.30
1	-6	6	325.37	3.60
1	-6	7	379.96	4.30
1	-6	8	122.59	1.80
1	-6	9	64.09	1.30
1	-6	10	734.43	9.40
1	-6	11	44.40	1.40
1	-7	-11	142.39	3.00
1	-7	-10	47.40	1.50
1	-7	-9	69.79	2.10
1	-7	-8	0.50	0.20
1	-7	-7	119.19	1.90
1	-7	-6	1089.29	14.10
1	-7	-5	227.18	2.90
1	-7	-4	100.89	1.60
1	-7	-3	106.59	1.70
1	-7	-2	0.50	0.20
1	-7	-1	17.10	0.90
1	-7	0	2753.22	37.60

1	-7	1	273.87	4.70
1	-7	2	1.40	0.20
1	-7	3	400.66	4.20
1	-7	4	1145.89	11.00
1	-7	5	61.19	1.10
1	-7	6	26.50	0.90
1	-7	7	70.69	1.40
1	-7	8	125.19	2.00
1	-7	9	444.96	6.00
1	-7	10	8.50	0.70
1	-8	-10	53.49	1.70
1	-8	-9	273.17	4.70
1	-8	-8	6.40	0.60
1	-8	-7	8.40	0.60
1	-8	-6	113.99	1.70
1	-8	-5	1.10	0.20
1	-8	-4	0.70	0.20
1	-8	-3	280.57	3.20
1	-8	-2	130.89	1.70
1	-8	-1	29.50	0.80
1	-8	0	336.07	3.50

1	-8	1	42.30	0.90
1	-8	2	14.90	0.50
1	-8	3	154.48	1.90
1	-8	4	228.78	3.10
1	-8	5	494.85	5.50
1	-8	6	3.70	0.40
1	-8	7	59.69	1.30
1	-8	8	190.88	2.80
1	-8	9	17.70	0.90
1	-8	10	161.38	3.80
1	-9	-9	30.80	1.60
1	-9	-8	3.50	0.40
1	-9	-7	386.46	5.10
1	-9	-6	26.80	0.90
1	-9	-5	18.80	0.80
1	-9	-4	639.94	7.10
1	-9	-3	848.72	8.40
1	-9	-2	95.49	1.50
1	-9	-1	383.06	5.60
1	-9	0	115.59	1.60
1	-9	1	41.80	1.20

1	-9	2	22.60	0.70
1	-9	3	500.75	5.20
1	-9	4	1.70	0.20
1	-9	5	3.30	0.50
1	-9	6	119.89	2.00
1	-9	7	258.77	3.60
1	-9	8	8.60	0.60
1	-9	9	37.60	1.30
1	-10	-8	113.69	2.80
1	-10	-7	40.50	1.10
1	-10	-6	226.98	3.20
1	-10	-5	1.40	0.20
1	-10	-4	0.80	0.20
1	-10	-3	117.99	1.70
1	-10	-2	2.60	0.20
1	-10	-1	319.07	3.30
1	-10	0	124.49	1.70
1	-10	1	1332.87	11.90
1	-10	2	273.17	3.30
1	-10	3	267.07	3.10
1	-10	4	30.80	0.80

1	-10	5	288.57	3.50
1	-10	6	125.29	2.10
1	-10	7	111.29	2.20
1	-10	8	65.59	1.80
1	-11	-7	25.90	0.80
1	-11	-6	107.89	1.90
1	-11	-5	81.29	1.60
1	-11	-4	397.56	4.70
1	-11	-3	0.80	0.20
1	-11	-2	22.70	0.70
1	-11	-1	94.29	1.40
1	-11	0	366.46	4.00
1	-11	1	0.30	0.10
1	-11	2	0.80	0.20
1	-11	3	5.90	0.50
1	-11	4	251.67	3.40
1	-11	5	337.27	4.20
1	-11	6	9.90	0.70
1	-11	7	56.49	1.90
1	-12	-5	28.40	1.00
1	-12	-4	64.89	1.30

1	-12	-3	0.50	0.10
1	-12	-2	253.87	3.20
1	-12	-1	134.69	2.10
1	-12	0	49.99	1.00
1	-12	1	57.19	1.00
1	-12	2	204.48	3.00
1	-12	3	36.00	0.80
1	-12	4	1.50	0.20
1	-12	5	0.10	0.20
1	-13	-3	17.60	0.60
1	-13	-2	1.30	0.20
1	-13	-1	213.08	2.80
1	-13	0	72.29	1.20
1	-13	1	13.80	0.50
1	-13	2	22.00	0.60
2	0	-13	0.10	0.10
2	0	-12	103.89	2.20
2	0	-11	0.10	0.20
2	0	-10	484.45	6.10
2	0	-9	0.30	0.20
2	0	-8	4000.10	44.80

2	0	-7	0.40	0.30
2	0	-6	4624.34	92.19
2	0	-5	0.50	0.40
2	0	-4	70.69	2.70
2	0	-3	2.60	0.90
2	0	-2	2350.16	47.10
2	0	-1	0.80	0.20
2	0	1	0.20	0.20
2	0	2	3972.70	98.69
2	0	3	0.60	0.30
2	0	4	2951.40	59.59
2	0	5	0.20	0.30
2	0	6	53.69	2.90
2	0	7	0.30	0.20
2	0	8	259.37	3.50
2	0	9	0.10	0.20
2	0	10	338.17	5.10
2	0	11	0.20	0.10
2	0	12	95.49	1.80
2	-1	-13	250.97	3.00
2	-1	-12	85.19	1.40

2	-1	-11	102.19	1.50
2	-1	-10	100.39	1.40
2	-1	-9	857.71	9.20
2	-1	-8	239.68	2.50
2	-1	-7	208.38	3.30
2	-1	-6	834.42	11.90
2	-1	-5	749.82	10.60
2	-1	-4	71.69	1.50
2	-1	-3	146.99	2.70
2	-1	-2	5714.03	99.99
2	-1	-1	1322.97	19.00
2	-1	0	5995.00	147.19
2	-1	1	2566.74	33.70
2	-1	2	372.76	5.00
2	-1	3	782.22	12.80
2	-1	4	19.90	0.90
2	-1	5	2329.67	41.80
2	-1	6	147.29	3.50
2	-1	7	142.39	1.70
2	-1	8	480.25	4.30
2	-1	9	244.78	2.60

2	-1	10	484.15	4.80
2	-1	11	79.29	1.20
2	-1	12	0.70	0.10
2	-2	-13	101.49	1.40
2	-2	-12	22.40	0.60
2	-2	-11	144.39	1.90
2	-2	-10	112.09	1.70
2	-2	-9	10.80	0.50
2	-2	-8	27.20	0.70
2	-2	-7	9.30	0.60
2	-2	-6	96.59	1.70
2	-2	-5	368.06	6.80
2	-2	-4	1663.03	26.50
2	-2	-3	3363.26	47.70
2	-2	-2	0.80	0.10
2	-2	-1	5797.12	139.09
2	-2	0	6154.88	95.29
2	-2	1	562.24	7.30
2	-2	2	298.67	4.40
2	-2	3	1014.20	13.90
2	-2	4	700.73	11.70

2	-2	5	107.89	2.10
2	-2	6	36.90	1.30
2	-2	7	210.08	2.20
2	-2	8	160.58	1.90
2	-2	9	383.86	3.50
2	-2	10	6.90	0.50
2	-2	11	417.06	4.90
2	-2	12	108.19	1.50
2	-3	-12	0.60	0.10
2	-3	-11	0.30	0.10
2	-3	-10	47.00	1.00
2	-3	-9	29.20	0.80
2	-3	-8	87.99	1.20
2	-3	-7	41.20	0.90
2	-3	-6	669.73	9.20
2	-3	-5	1360.26	18.70
2	-3	-4	24.70	1.40
2	-3	-3	1042.50	14.30
2	-3	-2	250.37	3.30
2	-3	-1	562.84	7.80
2	-3	0	897.31	13.10

2	-3	1	675.73	9.30
2	-3	2	2.90	0.30
2	-3	3	1065.49	14.50
2	-3	4	4488.95	64.89
2	-3	5	998.30	13.20
2	-3	6	169.88	2.20
2	-3	7	224.68	2.40
2	-3	8	8.40	0.50
2	-3	9	140.99	1.80
2	-3	10	307.97	3.20
2	-3	11	6.40	0.40
2	-3	12	9.10	0.40
2	-4	-12	12.90	0.50
2	-4	-11	414.16	4.50
2	-4	-10	202.28	2.60
2	-4	-9	151.18	1.90
2	-4	-8	163.98	1.90
2	-4	-7	115.59	1.60
2	-4	-6	175.38	3.70
2	-4	-5	724.53	11.60
2	-4	-4	96.89	2.00

2	-4	-3	1862.81	22.10
2	-4	-2	1089.99	13.90
2	-4	-1	12.20	0.70
2	-4	0	2213.88	38.90
2	-4	1	2019.20	32.00
2	-4	2	1738.63	23.40
2	-4	3	171.48	2.80
2	-4	4	255.67	3.70
2	-4	5	1914.31	24.40
2	-4	6	7.10	0.40
2	-4	7	546.45	4.70
2	-4	8	928.31	7.50
2	-4	9	47.10	1.10
2	-4	10	8.00	0.50
2	-4	11	146.49	1.90
2	-5	-12	2.90	0.40
2	-5	-11	1.50	0.20
2	-5	-10	0.40	0.10
2	-5	-9	768.82	7.60
2	-5	-8	432.36	4.20
2	-5	-7	38.10	0.80

2	-5	-6	150.58	2.20
2	-5	-5	98.19	2.00
2	-5	-4	85.49	1.50
2	-5	-3	205.38	2.70
2	-5	-2	663.43	11.30
2	-5	-1	25.00	1.20
2	-5	0	20.60	1.00
2	-5	1	926.71	17.00
2	-5	2	1423.86	19.60
2	-5	3	320.97	4.70
2	-5	4	60.89	1.50
2	-5	5	7.60	0.50
2	-5	6	1.20	0.20
2	-5	7	253.37	3.00
2	-5	8	75.99	1.20
2	-5	9	8.10	0.50
2	-5	10	351.56	4.20
2	-5	11	36.20	0.90
2	-6	-11	221.78	4.40
2	-6	-10	116.99	2.50
2	-6	-9	142.29	2.30

2	-6	-8	176.48	2.40
2	-6	-7	85.69	1.70
2	-6	-6	311.67	3.60
2	-6	-5	32.60	1.00
2	-6	-4	594.84	9.30
2	-6	-3	4.30	0.50
2	-6	-2	33.10	1.20
2	-6	-1	1276.87	23.70
2	-6	0	1947.51	31.70
2	-6	1	14.20	0.90
2	-6	2	680.53	10.80
2	-6	3	518.95	8.40
2	-6	4	523.65	5.80
2	-6	5	0.90	0.20
2	-6	6	617.94	6.10
2	-6	7	82.09	1.30
2	-6	8	306.37	3.80
2	-6	9	143.19	2.10
2	-6	10	256.17	3.80
2	-6	11	30.90	1.40
2	-7	-11	0.10	0.20

2	-7	-10	134.29	3.50
2	-7	-9	5.00	0.70
2	-7	-8	0.70	0.20
2	-7	-7	133.19	2.10
2	-7	-6	383.76	6.40
2	-7	-5	1495.45	14.60
2	-7	-4	623.44	6.30
2	-7	-3	428.56	6.00
2	-7	-2	15.40	0.60
2	-7	-1	3.00	0.30
2	-7	0	210.18	3.80
2	-7	1	253.97	3.10
2	-7	2	2197.18	21.10
2	-7	3	27.20	0.80
2	-7	4	2.90	0.30
2	-7	5	159.88	2.60
2	-7	6	9.70	0.60
2	-7	7	31.80	1.10
2	-7	8	12.90	0.70
2	-7	9	0.40	0.20
2	-7	10	198.78	7.00

2	-8	-10	5.00	0.60
2	-8	-9	4.90	0.70
2	-8	-8	245.98	3.50
2	-8	-7	515.95	6.10
2	-8	-6	137.29	1.90
2	-8	-5	7.80	0.60
2	-8	-4	584.94	6.00
2	-8	-3	998.70	9.40
2	-8	-2	324.67	3.20
2	-8	-1	632.24	5.60
2	-8	0	46.40	0.90
2	-8	1	511.35	4.90
2	-8	2	256.57	2.80
2	-8	3	363.96	4.00
2	-8	4	238.88	2.80
2	-8	5	77.89	1.30
2	-8	6	0.50	0.20
2	-8	7	218.18	3.00
2	-8	8	252.87	3.70
2	-8	9	4.40	0.50
2	-9	-9	182.78	5.40

2	-9	-8	246.88	5.60
2	-9	-7	151.58	2.40
2	-9	-6	43.40	1.10
2	-9	-5	334.97	4.00
2	-9	-4	55.09	1.00
2	-9	-3	346.87	4.00
2	-9	-2	31.20	0.90
2	-9	-1	134.79	2.10
2	-9	0	15.80	0.60
2	-9	1	44.20	0.90
2	-9	2	23.10	0.80
2	-9	3	237.48	2.60
2	-9	4	25.50	0.70
2	-9	5	571.04	7.00
2	-9	6	88.09	1.70
2	-9	7	307.47	4.50
2	-9	8	18.00	0.80
2	-9	9	172.38	4.40
2	-10	-8	0.50	0.20
2	-10	-7	0.80	0.20
2	-10	-6	170.38	2.80

2	-10	-5	0.50	0.20
2	-10	-4	235.98	3.20
2	-10	-3	89.59	1.40
2	-10	-2	42.20	0.90
2	-10	-1	330.77	3.80
2	-10	0	63.59	1.20
2	-10	1	11.00	0.60
2	-10	2	23.00	0.70
2	-10	3	383.66	4.50
2	-10	4	61.09	1.20
2	-10	5	41.00	0.90
2	-10	6	0.10	0.10
2	-10	7	649.14	8.30
2	-10	8	46.40	1.60
2	-11	-7	0.90	0.20
2	-11	-6	367.96	5.50
2	-11	-5	199.68	2.90
2	-11	-4	7.90	0.50
2	-11	-3	1.80	0.20
2	-11	-2	351.46	3.90
2	-11	-1	2.50	0.20

2	-11	0	131.89	2.10
2	-11	1	35.60	1.00
2	-11	2	583.24	5.80
2	-11	3	18.40	0.60
2	-11	4	71.69	1.20
2	-11	5	94.89	1.90
2	-11	6	73.49	1.90
2	-12	-5	8.70	0.50
2	-12	-4	350.96	4.40
2	-12	-3	17.20	0.60
2	-12	-2	2.70	0.40
2	-12	-1	11.80	0.50
2	-12	0	217.98	3.00
2	-12	1	252.07	3.20
2	-12	2	12.40	0.50
2	-12	3	9.90	0.40
2	-12	4	364.56	7.40
2	-12	5	0.30	0.20
2	-13	-2	187.28	2.80
2	-13	-1	504.25	6.50
2	-13	0	31.50	0.80

2	-13	1	376.36	5.70
2	-13	2	268.87	3.80
3	0	-13	0.10	0.10
3	0	-12	656.13	10.40
3	0	-11	0.10	0.20
3	0	-10	63.89	1.50
3	0	-9	0.60	0.20
3	0	-8	288.57	4.00
3	0	-7	0.20	0.30
3	0	-6	441.16	10.30
3	0	-5	0.90	0.40
3	0	-4	140.09	4.70
3	0	-3	1.80	0.70
3	0	-2	2694.43	53.99
3	0	-1	1.20	0.30
3	0	0	2396.16	41.90
3	0	1	0.40	0.20
3	0	2	1589.34	32.50
3	0	3	0.50	0.30
3	0	4	463.85	13.30
3	0	5	0.20	0.40

3	0	6	1452.15	20.80
3	0	7	0.20	0.20
3	0	8	153.98	2.70
3	0	9	0.20	0.20
3	0	10	50.69	1.30
3	0	11	0.20	0.20
3	0	12	440.26	7.80
3	-1	-13	0.10	0.10
3	-1	-12	0.50	0.10
3	-1	-11	30.70	0.80
3	-1	-10	22.20	0.60
3	-1	-9	322.07	3.80
3	-1	-8	295.87	3.30
3	-1	-7	16.60	0.80
3	-1	-6	373.76	5.90
3	-1	-5	513.55	8.20
3	-1	-4	5403.76	76.09
3	-1	-3	35.90	1.00
3	-1	-2	3905.71	54.99
3	-1	-1	2253.47	29.90
3	-1	0	2617.84	32.20

3	-1	1	509.95	7.30
3	-1	2	739.23	10.30
3	-1	3	797.02	12.10
3	-1	4	287.67	5.60
3	-1	5	178.28	4.00
3	-1	6	457.15	4.50
3	-1	7	2.60	0.20
3	-1	8	503.15	4.40
3	-1	9	38.90	0.90
3	-1	10	83.29	1.30
3	-1	11	0.20	0.10
3	-1	12	722.03	7.70
3	-2	-13	48.40	1.00
3	-2	-12	19.00	0.70
3	-2	-11	51.19	1.00
3	-2	-10	201.88	2.50
3	-2	-9	435.26	4.10
3	-2	-8	206.58	2.20
3	-2	-7	111.09	1.70
3	-2	-6	2365.46	29.80
3	-2	-5	1368.46	16.50

3	-2	-4	175.08	3.20
3	-2	-3	24.70	0.90
3	-2	-2	1937.71	27.70
3	-2	-1	2610.64	37.30
3	-2	0	590.74	8.40
3	-2	1	1696.03	22.60
3	-2	2	1028.40	14.10
3	-2	3	245.68	4.00
3	-2	4	526.55	7.90
3	-2	5	324.37	5.00
3	-2	6	27.90	0.60
3	-2	7	170.48	1.80
3	-2	8	164.28	1.80
3	-2	9	331.27	3.40
3	-2	10	56.89	1.00
3	-2	11	66.49	1.00
3	-2	12	61.29	1.10
3	-3	-13	0.10	0.20
3	-3	-12	24.90	0.60
3	-3	-11	688.93	7.10
3	-3	-10	208.98	2.60

3	-3	-9	37.10	0.80
3	-3	-8	157.58	1.80
3	-3	-7	1313.97	12.00
3	-3	-6	68.79	1.40
3	-3	-5	0.50	0.20
3	-3	-4	155.78	2.50
3	-3	-3	78.49	1.50
3	-3	-2	2.20	0.30
3	-3	-1	2509.05	33.20
3	-3	0	25.30	0.90
3	-3	1	1378.86	18.60
3	-3	2	1338.47	19.90
3	-3	3	508.05	7.10
3	-3	4	490.95	7.00
3	-3	5	550.84	7.90
3	-3	6	0.30	0.10
3	-3	7	424.86	3.90
3	-3	8	14.50	0.50
3	-3	9	2.30	0.30
3	-3	10	27.00	0.70
3	-3	11	39.80	0.70

3	-4	-12	107.09	1.80
3	-4	-11	80.19	1.30
3	-4	-10	57.39	1.10
3	-4	-9	250.37	2.70
3	-4	-8	226.28	2.40
3	-4	-7	51.79	1.00
3	-4	-6	487.05	6.60
3	-4	-5	523.95	6.90
3	-4	-4	935.11	11.00
3	-4	-3	257.27	3.70
3	-4	-2	1501.45	19.20
3	-4	-1	59.49	1.90
3	-4	0	318.97	5.80
3	-4	1	1804.92	24.20
3	-4	2	1593.54	21.60
3	-4	3	625.84	8.60
3	-4	4	236.98	4.00
3	-4	5	1034.70	9.50
3	-4	6	375.06	3.50
3	-4	7	206.08	2.50
3	-4	8	188.78	2.10

3	-4	9	306.17	3.60
3	-4	10	39.00	0.80
3	-4	11	0.20	0.10
3	-5	-12	270.47	4.90
3	-5	-11	36.70	0.90
3	-5	-10	110.59	1.60
3	-5	-9	200.08	2.50
3	-5	-8	650.13	6.50
3	-5	-7	44.40	0.90
3	-5	-6	8.60	0.50
3	-5	-5	143.09	2.60
3	-5	-4	257.67	3.60
3	-5	-3	5.60	0.70
3	-5	-2	6.70	0.60
3	-5	-1	356.26	6.50
3	-5	0	43.50	1.50
3	-5	1	660.13	10.30
3	-5	2	660.03	10.40
3	-5	3	159.98	2.80
3	-5	4	2887.41	29.60
3	-5	5	1191.98	10.40

3	-5	6	1.80	0.20
3	-5	7	187.98	2.30
3	-5	8	49.90	1.00
3	-5	9	0.10	0.10
3	-5	10	10.90	0.60
3	-5	11	79.69	2.70
3	-6	-11	26.50	1.10
3	-6	-10	6.80	0.60
3	-6	-9	11.60	0.60
3	-6	-8	618.84	7.20
3	-6	-7	595.64	7.30
3	-6	-6	700.93	7.00
3	-6	-5	124.19	1.80
3	-6	-4	3.00	0.40
3	-6	-3	243.28	4.20
3	-6	-2	185.88	3.30
3	-6	-1	159.88	2.90
3	-6	0	2866.01	41.70
3	-6	1	636.14	10.20
3	-6	2	2519.95	36.90
3	-6	3	0.70	0.20

3	-6	4	139.99	2.00
3	-6	5	192.98	2.40
3	-6	6	2.20	0.20
3	-6	7	47.10	1.00
3	-6	8	150.78	2.10
3	-6	9	19.10	0.80
3	-6	10	879.31	11.10
3	-7	-11	3.10	0.70
3	-7	-10	9.20	0.80
3	-7	-9	16.60	0.80
3	-7	-8	131.79	2.70
3	-7	-7	50.19	1.20
3	-7	-6	203.78	2.80
3	-7	-5	36.80	1.00
3	-7	-4	861.31	8.40
3	-7	-3	694.43	7.00
3	-7	-2	173.68	2.50
3	-7	-1	101.79	1.50
3	-7	0	40.90	0.90
3	-7	1	751.32	7.40
3	-7	2	26.60	0.70

3	-7	3	2.90	0.30
3	-7	4	9.80	0.60
3	-7	5	49.10	1.10
3	-7	6	97.29	1.50
3	-7	7	332.17	4.10
3	-7	8	105.29	1.70
3	-7	9	32.10	1.00
3	-7	10	45.30	1.70
3	-8	-10	9.60	0.70
3	-8	-9	201.98	4.00
3	-8	-8	11.60	0.70
3	-8	-7	12.20	0.70
3	-8	-6	208.68	2.80
3	-8	-5	45.00	1.20
3	-8	-4	275.07	3.50
3	-8	-3	28.00	0.70
3	-8	-2	0.70	0.20
3	-8	-1	789.82	7.00
3	-8	0	0.20	0.10
3	-8	1	82.99	1.20
3	-8	2	923.71	8.00

3	-8	3	163.88	2.00
3	-8	4	131.99	2.00
3	-8	5	20.00	0.70
3	-8	6	22.50	0.80
3	-8	7	86.19	1.50
3	-8	8	0.90	0.20
3	-8	9	675.93	13.60
3	-9	-9	1.40	0.30
3	-9	-8	151.38	3.20
3	-9	-7	78.89	1.60
3	-9	-6	72.99	1.50
3	-9	-5	198.88	2.90
3	-9	-4	92.59	1.40
3	-9	-3	1503.95	12.80
3	-9	-2	41.40	0.90
3	-9	-1	1603.74	14.30
3	-9	0	40.30	0.90
3	-9	1	42.00	1.00
3	-9	2	115.79	1.50
3	-9	3	38.90	0.80
3	-9	4	34.70	0.90

3	-9	5	336.57	3.80
3	-9	6	14.80	0.60
3	-9	7	220.38	3.30
3	-9	8	3.90	0.40
3	-10	-8	54.29	1.80
3	-10	-7	33.40	1.00
3	-10	-6	246.78	3.80
3	-10	-5	962.00	10.50
3	-10	-4	54.09	1.20
3	-10	-3	22.70	0.70
3	-10	-2	66.19	1.30
3	-10	-1	694.53	7.10
3	-10	0	18.70	0.70
3	-10	1	310.07	3.70
3	-10	2	286.87	3.50
3	-10	3	417.56	4.60
3	-10	4	162.28	2.20
3	-10	5	139.99	2.20
3	-10	6	49.50	1.10
3	-10	7	53.39	1.20
3	-11	-7	38.20	1.10

3	-11	-6	232.88	3.70
3	-11	-5	59.59	1.40
3	-11	-4	311.37	4.30
3	-11	-3	530.75	6.70
3	-11	-2	63.19	1.20
3	-11	-1	1.00	0.20
3	-11	0	2.60	0.20
3	-11	1	1.30	0.20
3	-11	2	16.60	0.60
3	-11	3	118.89	2.10
3	-11	4	350.16	4.70
3	-11	5	325.87	4.50
3	-11	6	4.00	0.60
3	-12	-5	3.30	0.40
3	-12	-4	8.50	0.50
3	-12	-3	41.70	0.90
3	-12	-2	51.99	0.90
3	-12	-1	337.17	3.80
3	-12	0	0.20	0.10
3	-12	1	74.49	1.10
3	-12	2	157.48	2.30

3	-12	3	0.80	0.10
3	-12	4	47.90	1.10
3	-13	-2	11.20	0.50
3	-13	-1	27.20	0.70
3	-13	0	68.09	1.40
4	0	-13	0.00	0.10
4	0	-12	210.48	4.10
4	0	-11	0.10	0.20
4	0	-10	224.18	3.40
4	0	-9	0.10	0.20
4	0	-8	1053.09	12.90
4	0	-7	0.70	0.20
4	0	-6	212.98	5.80
4	0	-5	0.20	0.40
4	0	-3	1.60	0.70
4	0	-2	0.70	0.30
4	0	-1	0.50	0.20
4	0	0	5145.99	89.19
4	0	1	0.40	0.30
4	0	2	214.18	5.70
4	0	3	0.50	0.30

4	0	4	130.39	4.20
4	0	5	0.20	0.30
4	0	6	655.33	7.40
4	0	7	0.20	0.20
4	0	8	2.40	0.30
4	0	9	0.30	0.20
4	0	10	133.49	2.40
4	0	11	0.10	0.10
4	-1	-13	93.59	1.40
4	-1	-12	185.68	2.70
4	-1	-11	1.70	0.20
4	-1	-10	689.03	6.10
4	-1	-9	46.40	0.90
4	-1	-8	1.50	0.20
4	-1	-7	652.93	6.40
4	-1	-6	702.63	10.20
4	-1	-5	2089.29	30.10
4	-1	-4	939.71	17.20
4	-1	-3	187.08	3.80
4	-1	-2	793.82	13.00
4	-1	-1	320.77	5.30

4	-1	0	887.41	11.50
4	-1	1	204.88	3.40
4	-1	2	1505.35	20.50
4	-1	3	151.88	3.20
4	-1	4	80.59	2.00
4	-1	5	259.67	4.40
4	-1	6	109.79	1.30
4	-1	7	39.90	0.80
4	-1	8	9.60	0.40
4	-1	9	98.09	1.90
4	-1	10	68.19	1.10
4	-1	11	0.30	0.10
4	-2	-13	1.40	0.20
4	-2	-12	117.89	1.90
4	-2	-11	141.09	1.90
4	-2	-10	41.90	0.90
4	-2	-9	74.19	1.20
4	-2	-8	90.89	1.30
4	-2	-7	1027.20	9.30
4	-2	-6	72.29	1.70
4	-2	-5	171.78	3.10

4	-2	-4	46.90	1.50
4	-2	-3	276.27	4.40
4	-2	-2	231.38	4.10
4	-2	-1	38.90	1.10
4	-2	0	2855.91	37.80
4	-2	1	41.20	1.20
4	-2	2	51.09	1.40
4	-2	3	2854.61	41.50
4	-2	4	318.47	5.40
4	-2	5	818.72	11.20
4	-2	6	5.20	0.30
4	-2	7	417.16	3.60
4	-2	8	417.86	4.00
4	-2	9	98.79	1.50
4	-2	10	28.70	0.70
4	-2	11	222.88	2.40
4	-3	-12	23.10	0.60
4	-3	-11	159.78	2.10
4	-3	-10	189.78	2.40
4	-3	-9	163.18	1.90
4	-3	-8	0.10	0.10

4	-3	-7	0.80	0.20
4	-3	-6	0.60	0.20
4	-3	-5	1516.75	19.60
4	-3	-4	262.07	3.70
4	-3	-3	112.99	2.30
4	-3	-2	60.49	1.60
4	-3	-1	2284.37	30.50
4	-3	0	560.34	8.80
4	-3	1	7.40	0.60
4	-3	2	1331.37	18.30
4	-3	3	636.64	9.50
4	-3	4	394.66	6.50
4	-3	5	11.80	0.50
4	-3	6	314.07	3.00
4	-3	7	323.07	3.50
4	-3	8	198.68	2.20
4	-3	9	957.50	8.80
4	-3	10	151.28	2.00
4	-3	11	11.60	0.50
4	-4	-12	0.60	0.10
4	-4	-11	209.28	2.90

4	-4	-10	0.60	0.10
4	-4	-9	116.09	1.50
4	-4	-8	0.50	0.20
4	-4	-7	999.60	9.00
4	-4	-6	33.40	0.90
4	-4	-5	124.39	2.40
4	-4	-4	15.80	0.70
4	-4	-3	1312.77	16.00
4	-4	-2	102.89	2.40
4	-4	-1	11.60	0.90
4	-4	0	72.59	2.00
4	-4	1	1935.31	26.30
4	-4	2	3029.00	37.90
4	-4	3	1.50	0.30
4	-4	4	109.09	1.80
4	-4	5	531.15	5.10
4	-4	6	160.98	1.80
4	-4	7	183.18	2.10
4	-4	8	8.40	0.50
4	-4	9	0.50	0.10
4	-4	10	1.60	0.20

4	-4	11	4.60	0.40
4	-5	-12	51.99	1.80
4	-5	-11	16.30	0.70
4	-5	-10	120.39	1.60
4	-5	-9	337.47	3.80
4	-5	-8	592.44	5.80
4	-5	-7	570.64	5.40
4	-5	-6	490.55	4.90
4	-5	-5	551.34	6.10
4	-5	-4	29.50	1.00
4	-5	-3	121.69	2.50
4	-5	-2	1424.86	19.60
4	-5	-1	222.78	4.20
4	-5	0	49.80	1.40
4	-5	1	12.10	0.80
4	-5	2	634.24	10.20
4	-5	3	213.18	3.00
4	-5	4	69.79	1.20
4	-5	5	3.50	0.30
4	-5	6	515.15	5.30
4	-5	7	6.40	0.40

4	-5	8	30.60	0.80
4	-5	9	53.09	1.00
4	-5	10	121.79	2.40
4	-6	-11	25.40	0.90
4	-6	-10	22.70	0.80
4	-6	-9	0.50	0.20
4	-6	-8	80.79	1.60
4	-6	-7	18.30	0.70
4	-6	-6	45.50	1.10
4	-6	-5	9.60	0.60
4	-6	-4	939.71	11.30
4	-6	-3	70.69	1.40
4	-6	-2	0.80	0.30
4	-6	-1	284.27	4.80
4	-6	0	408.66	6.50
4	-6	1	1283.37	17.00
4	-6	2	6.80	0.40
4	-6	3	26.50	0.70
4	-6	4	415.56	4.60
4	-6	5	53.19	1.20
4	-6	6	552.94	5.70

4	-6	7	466.05	5.50
4	-6	8	158.48	2.40
4	-6	9	7.00	0.50
4	-6	10	24.80	0.80
4	-7	-11	51.99	1.80
4	-7	-10	185.88	3.10
4	-7	-9	345.87	4.70
4	-7	-8	492.05	6.10
4	-7	-7	164.18	2.50
4	-7	-6	570.24	6.90
4	-7	-5	82.79	1.50
4	-7	-4	35.20	0.90
4	-7	-3	0.60	0.20
4	-7	-2	40.30	0.90
4	-7	-1	297.17	3.70
4	-7	0	4.00	0.30
4	-7	1	828.52	7.80
4	-7	2	1874.81	15.50
4	-7	3	285.07	3.00
4	-7	4	505.75	5.30
4	-7	5	15.50	0.70

4	-7	6	590.14	6.70
4	-7	7	268.07	3.40
4	-7	8	98.49	1.70
4	-7	9	63.79	1.30
4	-8	-10	47.20	1.90
4	-8	-9	42.60	1.30
4	-8	-8	1.40	0.20
4	-8	-7	365.76	4.70
4	-8	-6	4.40	0.40
4	-8	-5	108.09	1.90
4	-8	-4	343.27	3.90
4	-8	-3	248.98	2.90
4	-8	-2	523.05	5.00
4	-8	-1	273.97	2.90
4	-8	0	73.29	1.30
4	-8	1	44.30	0.90
4	-8	2	1122.79	9.90
4	-8	3	668.33	6.30
4	-8	4	10.40	0.60
4	-8	5	371.96	5.20
4	-8	6	257.57	3.30

4	-8	7	0.70	0.20
4	-8	8	5.00	0.50
4	-8	9	4.70	0.60
4	-9	-9	77.09	2.10
4	-9	-8	0.00	0.10
4	-9	-7	58.69	1.30
4	-9	-6	38.10	1.00
4	-9	-5	2.80	0.30
4	-9	-4	168.38	2.20
4	-9	-3	82.89	1.30
4	-9	-2	0.80	0.20
4	-9	-1	2817.12	24.40
4	-9	0	1.20	0.20
4	-9	1	217.88	2.50
4	-9	2	107.59	1.50
4	-9	3	112.09	1.70
4	-9	4	0.80	0.20
4	-9	5	29.10	0.80
4	-9	6	23.20	0.70
4	-9	7	30.00	0.80
4	-9	8	29.60	1.10

4	-10	-8	15.50	1.20
4	-10	-7	2.20	0.30
4	-10	-6	339.77	4.00
4	-10	-5	113.69	1.80
4	-10	-4	415.16	5.50
4	-10	-3	16.60	0.60
4	-10	-2	0.90	0.20
4	-10	-1	971.60	9.50
4	-10	0	94.19	1.50
4	-10	1	46.20	1.00
4	-10	2	175.68	2.10
4	-10	3	2.80	0.30
4	-10	4	186.28	2.50
4	-10	5	376.36	4.80
4	-10	6	0.80	0.10
4	-10	7	92.59	2.00
4	-11	-7	4.40	0.50
4	-11	-6	50.29	1.10
4	-11	-5	0.40	0.10
4	-11	-4	123.99	2.10
4	-11	-3	416.66	5.10

4	-11	-2	92.89	1.40
4	-11	-1	112.19	1.70
4	-11	0	263.97	3.10
4	-11	1	5.70	0.40
4	-11	2	231.58	2.90
4	-11	3	56.89	1.30
4	-11	4	0.20	0.10
4	-11	5	36.20	0.80
4	-12	-5	176.08	3.40
4	-12	-4	741.23	12.80
4	-12	-3	0.30	0.10
4	-12	-2	17.50	0.50
4	-12	-1	0.70	0.10
4	-12	0	1.70	0.20
4	-12	1	26.60	0.60
4	-12	2	55.69	1.00
4	-12	3	24.10	0.80
5	0	-13	0.10	0.10
5	0	-12	151.48	3.00
5	0	-11	0.20	0.20
5	0	-10	660.43	9.20

5	0	-9	0.20	0.20
5	0	-8	31.00	1.00
5	0	-7	0.20	0.20
5	0	-6	248.08	6.70
5	0	-5	0.20	0.30
5	0	-4	5517.35	133.99
5	0	-3	0.80	0.50
5	0	-2	210.38	5.50
5	0	-1	0.30	0.30
5	0	0	18.30	1.20
5	0	1	0.40	0.30
5	0	2	1896.81	39.40
5	0	3	0.20	0.30
5	0	4	210.58	5.20
5	0	5	0.30	0.20
5	0	6	1954.60	21.10
5	0	7	0.20	0.20
5	0	8	0.70	0.20
5	0	9	0.10	0.20
5	0	10	1018.20	15.00
5	0	11	0.00	0.10

5	-1	-13	26.90	0.60
5	-1	-12	73.29	1.30
5	-1	-11	12.20	0.50
5	-1	-10	274.37	3.10
5	-1	-9	38.50	0.80
5	-1	-8	34.80	0.80
5	-1	-7	688.93	7.00
5	-1	-6	31.70	1.10
5	-1	-5	66.79	1.80
5	-1	-4	296.67	6.50
5	-1	-3	15.00	0.90
5	-1	-2	11.50	0.80
5	-1	-1	115.99	2.20
5	-1	0	530.05	7.30
5	-1	1	1204.08	16.70
5	-1	2	292.67	5.40
5	-1	3	122.29	3.00
5	-1	4	1819.12	25.40
5	-1	5	2.10	0.20
5	-1	6	17.40	0.60
5	-1	7	348.67	3.30

5	-1	8	67.79	1.10
5	-1	9	2.80	0.20
5	-1	10	10.50	0.50
5	-1	11	182.28	2.30
5	-2	-13	301.27	3.90
5	-2	-12	122.79	1.70
5	-2	-11	8.90	0.50
5	-2	-10	447.66	4.60
5	-2	-9	474.15	4.20
5	-2	-8	148.49	1.80
5	-2	-7	243.58	2.80
5	-2	-6	373.16	5.70
5	-2	-5	323.97	5.70
5	-2	-4	826.32	11.80
5	-2	-3	1708.13	25.00
5	-2	-2	238.28	4.80
5	-2	-1	4416.96	63.29
5	-2	0	46.80	1.40
5	-2	1	490.45	7.50
5	-2	2	560.04	8.10
5	-2	3	829.42	12.20

5	-2	4	656.63	10.10
5	-2	5	60.59	0.90
5	-2	6	189.48	2.00
5	-2	7	59.09	1.00
5	-2	8	144.99	1.80
5	-2	9	251.57	2.90
5	-2	10	42.70	0.80
5	-2	11	36.60	0.80
5	-3	-12	13.10	0.50
5	-3	-11	71.69	1.10
5	-3	-10	0.20	0.20
5	-3	-9	30.30	0.80
5	-3	-8	174.58	2.10
5	-3	-7	2211.28	19.10
5	-3	-6	395.26	4.50
5	-3	-5	1219.78	16.00
5	-3	-4	260.37	4.50
5	-3	-3	423.56	6.20
5	-3	-2	513.95	7.80
5	-3	-1	123.99	3.10
5	-3	0	432.86	7.20

5	-3	1	10.80	0.70
5	-3	2	1506.55	19.60
5	-3	3	928.81	12.70
5	-3	4	642.14	5.70
5	-3	5	6.60	0.40
5	-3	6	24.70	0.60
5	-3	7	378.76	3.30
5	-3	8	5.30	0.40
5	-3	9	199.38	2.20
5	-3	10	17.30	0.50
5	-4	-12	173.58	2.50
5	-4	-11	26.50	0.70
5	-4	-10	6.70	0.50
5	-4	-9	66.39	1.20
5	-4	-8	277.07	3.10
5	-4	-7	145.49	1.80
5	-4	-6	165.58	2.10
5	-4	-5	9.80	0.60
5	-4	-4	425.86	6.00
5	-4	-3	1624.84	23.90
5	-4	-2	647.54	9.60

5	-4	-1	1723.63	23.60
5	-4	0	1097.69	14.50
5	-4	1	193.48	3.50
5	-4	2	98.79	2.00
5	-4	3	112.19	1.80
5	-4	4	171.48	2.00
5	-4	5	34.80	0.80
5	-4	6	114.69	1.50
5	-4	7	87.79	1.20
5	-4	8	65.69	1.10
5	-4	9	1070.29	10.00
5	-4	10	41.10	1.00
5	-5	-12	4.40	0.60
5	-5	-11	57.89	1.20
5	-5	-10	14.10	0.50
5	-5	-9	51.09	1.30
5	-5	-8	29.30	0.80
5	-5	-7	139.59	2.20
5	-5	-6	662.03	6.50
5	-5	-5	201.18	2.70
5	-5	-4	1111.09	11.90

5	-5	-3	877.31	11.80
5	-5	-2	339.27	5.20
5	-5	-1	0.90	0.30
5	-5	0	79.39	1.90
5	-5	1	1.40	0.30
5	-5	2	88.69	1.40
5	-5	3	8.30	0.50
5	-5	4	348.37	3.70
5	-5	5	1159.18	13.10
5	-5	6	126.19	1.60
5	-5	7	154.88	2.00
5	-5	8	78.39	1.40
5	-5	9	0.80	0.20
5	-5	10	11.80	0.50
5	-6	-11	115.79	2.00
5	-6	-10	129.49	2.40
5	-6	-9	117.89	2.00
5	-6	-8	344.57	4.20
5	-6	-7	395.86	4.70
5	-6	-6	352.76	6.00
5	-6	-5	0.90	0.20

5	-6	-4	287.57	3.40
5	-6	-3	299.07	4.70
5	-6	-2	155.78	2.20
5	-6	-1	3.80	0.30
5	-6	0	154.68	2.00
5	-6	1	338.87	3.80
5	-6	2	400.86	4.00
5	-6	3	956.20	9.00
5	-6	4	348.87	4.30
5	-6	5	430.86	4.50
5	-6	6	381.96	4.10
5	-6	7	39.80	0.90
5	-6	8	0.40	0.10
5	-6	9	30.40	0.80
5	-7	-11	66.29	2.20
5	-7	-10	179.48	2.80
5	-7	-9	261.67	3.70
5	-7	-8	1.50	0.20
5	-7	-7	101.69	1.80
5	-7	-6	111.79	1.80
5	-7	-5	19.90	0.70

5	-7	-4	192.38	2.50
5	-7	-3	8.10	0.50
5	-7	-2	14.50	0.60
5	-7	-1	119.79	1.60
5	-7	0	1553.44	13.00
5	-7	1	2776.42	23.20
5	-7	2	296.07	3.10
5	-7	3	555.74	5.60
5	-7	4	282.47	3.30
5	-7	5	0.30	0.10
5	-7	6	0.30	0.10
5	-7	7	39.10	0.80
5	-7	8	113.89	1.60
5	-7	9	154.08	2.60
5	-8	-10	168.38	3.40
5	-8	-9	594.34	7.90
5	-8	-8	43.80	1.10
5	-8	-7	164.28	2.70
5	-8	-6	260.97	3.40
5	-8	-5	0.80	0.20
5	-8	-4	32.40	0.90

5	-8	-3	103.39	1.60
5	-8	-2	91.19	1.50
5	-8	-1	456.45	6.50
5	-8	0	76.29	1.50
5	-8	1	755.92	6.90
5	-8	2	47.50	0.90
5	-8	3	22.50	0.70
5	-8	4	1097.49	11.00
5	-8	5	30.70	0.80
5	-8	6	7.60	0.50
5	-8	7	375.46	4.40
5	-8	8	17.90	0.80
5	-9	-9	27.50	0.90
5	-9	-8	10.80	0.60
5	-9	-7	246.48	3.40
5	-9	-6	34.80	0.90
5	-9	-5	0.60	0.20
5	-9	-4	1.10	0.20
5	-9	-3	161.18	2.10
5	-9	-2	37.30	0.80
5	-9	-1	0.50	0.10

5	-9	0	13.80	0.60
5	-9	1	69.89	1.20
5	-9	2	4.60	0.40
5	-9	3	75.09	1.30
5	-9	4	1.10	0.20
5	-9	5	4.90	0.40
5	-9	6	7.10	0.50
5	-9	7	152.78	3.20
5	-10	-8	19.80	1.00
5	-10	-7	11.40	0.60
5	-10	-6	235.08	3.60
5	-10	-5	514.45	5.90
5	-10	-4	52.99	1.20
5	-10	-3	209.68	2.50
5	-10	-2	207.38	2.50
5	-10	-1	140.89	1.80
5	-10	0	8.10	0.50
5	-10	1	62.39	1.10
5	-10	2	50.49	1.00
5	-10	3	114.99	1.80
5	-10	4	11.90	0.60

5	-10	5	105.89	1.70
5	-10	6	60.09	1.20
5	-11	-6	21.70	1.00
5	-11	-5	101.29	1.70
5	-11	-4	814.62	13.00
5	-11	-3	89.09	1.60
5	-11	-2	161.68	2.10
5	-11	-1	0.30	0.10
5	-11	0	34.60	0.70
5	-11	1	124.09	1.60
5	-11	2	37.50	1.00
5	-11	3	20.40	0.60
5	-11	4	179.28	2.80
5	-12	-4	13.60	0.70
5	-12	-3	1.90	0.20
5	-12	-2	402.46	6.00
5	-12	-1	241.28	3.30
5	-12	0	5.90	0.40
5	-12	1	3.60	0.30
5	-12	2	288.37	4.00
6	0	-13	0.20	0.10

6	0	-12	321.27	5.00
6	0	-11	0.20	0.20
6	0	-10	269.57	4.20
6	0	-9	0.30	0.20
6	0	-8	438.96	6.30
6	0	-7	0.30	0.20
6	0	-6	860.11	13.10
6	0	-5	0.70	0.30
6	0	-4	88.19	2.60
6	0	-3	0.20	0.30
6	0	-2	92.59	2.90
6	0	-1	0.30	0.40
6	0	0	234.18	5.50
6	0	1	0.30	0.30
6	0	2	1650.13	30.50
6	0	3	0.20	0.30
6	0	4	1592.04	17.30
6	0	5	0.30	0.20
6	0	6	593.94	9.40
6	0	7	0.10	0.20
6	0	8	29.10	1.00

6	0	9	0.10	0.20
6	0	10	275.77	4.60
6	-1	-13	152.58	2.30
6	-1	-12	489.35	5.30
6	-1	-11	0.40	0.10
6	-1	-10	386.16	4.00
6	-1	-9	32.50	0.70
6	-1	-8	3.70	0.30
6	-1	-7	32.10	0.80
6	-1	-6	683.43	7.20
6	-1	-5	331.47	5.60
6	-1	-4	346.67	5.60
6	-1	-3	362.66	6.30
6	-1	-2	4151.48	64.69
6	-1	-1	184.68	3.50
6	-1	0	984.30	13.10
6	-1	1	292.97	4.40
6	-1	2	39.40	1.20
6	-1	3	48.00	1.40
6	-1	4	349.67	3.40
6	-1	5	17.60	0.60

6	-1	6	300.17	3.30
6	-1	7	267.17	2.60
6	-1	8	173.68	2.00
6	-1	9	6.70	0.40
6	-1	10	163.68	2.10
6	-2	-12	44.10	0.80
6	-2	-11	52.79	1.00
6	-2	-10	8.70	0.60
6	-2	-9	2.90	0.30
6	-2	-8	214.78	2.50
6	-2	-7	379.16	3.80
6	-2	-6	156.98	2.00
6	-2	-5	1124.39	15.90
6	-2	-4	55.79	1.50
6	-2	-3	1.90	0.30
6	-2	-2	5.90	0.60
6	-2	-1	131.29	2.40
6	-2	0	916.21	11.60
6	-2	1	817.02	11.20
6	-2	2	30.90	1.10
6	-2	3	596.84	8.70

6	-2	4	277.37	2.70
6	-2	5	33.40	0.70
6	-2	6	0.90	0.10
6	-2	7	98.29	1.30
6	-2	8	47.10	0.90
6	-2	9	42.70	0.80
6	-2	10	0.20	0.10
6	-3	-12	0.80	0.10
6	-3	-11	54.19	1.00
6	-3	-10	9.80	0.50
6	-3	-9	134.09	1.70
6	-3	-8	7.60	0.50
6	-3	-7	163.88	2.10
6	-3	-6	163.98	2.20
6	-3	-5	380.86	4.40
6	-3	-4	190.98	3.10
6	-3	-3	69.79	1.40
6	-3	-2	433.96	6.90
6	-3	-1	1286.57	16.90
6	-3	0	492.85	7.10
6	-3	1	1558.74	20.20

6	-3	2	174.08	3.00
6	-3	3	1318.37	10.60
6	-3	4	13.00	0.50
6	-3	5	62.39	0.90
6	-3	6	20.30	0.60
6	-3	7	477.55	4.50
6	-3	8	0.80	0.10
6	-3	9	290.47	3.50
6	-3	10	7.50	0.40
6	-4	-12	126.69	1.90
6	-4	-11	145.29	2.10
6	-4	-10	413.56	4.30
6	-4	-9	292.07	3.40
6	-4	-8	1.60	0.20
6	-4	-7	1024.40	8.70
6	-4	-6	672.03	6.40
6	-4	-5	194.48	2.40
6	-4	-4	846.82	10.00
6	-4	-3	449.06	6.60
6	-4	-2	46.20	1.30
6	-4	-1	12.50	0.70

6	-4	0	1167.98	15.50
6	-4	1	180.68	3.20
6	-4	2	404.66	4.10
6	-4	3	0.70	0.20
6	-4	4	575.34	5.20
6	-4	5	315.87	3.10
6	-4	6	2.30	0.20
6	-4	7	4.20	0.40
6	-4	8	92.69	1.50
6	-4	9	67.79	1.10
6	-4	10	17.80	0.50
6	-5	-12	74.89	1.90
6	-5	-11	5.00	0.40
6	-5	-10	587.34	6.60
6	-5	-9	218.98	2.80
6	-5	-8	92.99	1.50
6	-5	-7	22.70	0.70
6	-5	-6	50.89	1.00
6	-5	-5	33.40	0.80
6	-5	-4	867.41	8.40
6	-5	-3	338.67	3.80

6	-5	-2	625.84	7.60
6	-5	-1	2.40	0.30
6	-5	0	1.10	0.20
6	-5	1	77.09	1.40
6	-5	2	205.28	2.30
6	-5	3	0.30	0.10
6	-5	4	328.47	4.40
6	-5	5	36.60	0.90
6	-5	6	1400.06	13.20
6	-5	7	185.78	2.60
6	-5	8	10.30	0.50
6	-5	9	11.80	0.50
6	-6	-11	0.10	0.20
6	-6	-10	159.48	2.40
6	-6	-9	121.39	2.00
6	-6	-8	145.19	2.30
6	-6	-7	243.28	3.20
6	-6	-6	39.90	1.00
6	-6	-5	0.40	0.20
6	-6	-4	639.54	6.80
6	-6	-3	90.89	1.30

6	-6	-2	0.90	0.20
6	-6	-1	644.24	5.90
6	-6	0	2139.09	17.10
6	-6	1	26.70	0.70
6	-6	2	225.88	2.70
6	-6	3	257.17	3.60
6	-6	4	189.18	2.30
6	-6	5	11.90	0.60
6	-6	6	0.50	0.10
6	-6	7	231.78	2.70
6	-6	8	146.59	2.10
6	-6	9	18.70	0.60
6	-7	-10	9.80	0.60
6	-7	-9	63.89	1.40
6	-7	-8	46.90	1.10
6	-7	-7	608.54	7.90
6	-7	-6	443.96	5.20
6	-7	-5	19.90	0.80
6	-7	-4	144.99	2.00
6	-7	-3	62.09	1.10
6	-7	-2	165.58	2.00

6	-7	-1	71.69	1.10
6	-7	0	30.90	0.70
6	-7	1	0.40	0.20
6	-7	2	7.40	0.50
6	-7	3	0.60	0.20
6	-7	4	0.80	0.20
6	-7	5	70.19	1.20
6	-7	6	43.70	0.90
6	-7	7	84.49	1.50
6	-7	8	1.30	0.20
6	-8	-10	206.78	4.30
6	-8	-9	0.20	0.10
6	-8	-8	8.60	0.50
6	-8	-7	576.64	7.00
6	-8	-6	124.69	2.00
6	-8	-5	195.38	2.80
6	-8	-4	150.38	2.10
6	-8	-3	890.11	8.80
6	-8	-2	88.29	1.30
6	-8	-1	336.47	3.50
6	-8	0	49.00	0.90

6	-8	1	247.58	2.90
6	-8	2	10.60	0.50
6	-8	3	500.65	5.60
6	-8	4	213.38	2.80
6	-8	5	21.90	0.70
6	-8	6	178.38	2.50
6	-8	7	263.67	4.40
6	-9	-9	464.15	7.70
6	-9	-8	34.90	0.80
6	-9	-7	162.58	2.70
6	-9	-6	16.60	0.70
6	-9	-5	428.06	5.10
6	-9	-4	102.29	1.70
6	-9	-3	32.00	1.00
6	-9	-2	349.37	3.70
6	-9	-1	447.46	4.50
6	-9	0	101.69	1.50
6	-9	1	184.48	2.70
6	-9	2	13.60	0.60
6	-9	3	950.60	10.40
6	-9	4	25.60	0.70

6	-9	5	154.78	2.40
6	-9	6	142.29	2.00
6	-10	-8	0.20	0.10
6	-10	-7	289.77	4.40
6	-10	-6	96.19	1.60
6	-10	-5	305.57	4.00
6	-10	-4	1.30	0.20
6	-10	-3	311.67	3.60
6	-10	-2	51.99	1.00
6	-10	-1	0.40	0.10
6	-10	0	15.20	0.50
6	-10	1	162.08	2.00
6	-10	2	58.39	1.00
6	-10	3	181.68	2.70
6	-10	4	105.99	1.50
6	-10	5	23.50	0.70
6	-11	-6	3.70	0.80
6	-11	-5	4.90	0.40
6	-11	-4	91.89	1.50
6	-11	-3	238.78	3.40
6	-11	-2	249.58	3.50

6	-11	-1	1.20	0.10
6	-11	0	20.70	0.60
6	-11	1	20.30	0.60
6	-11	2	208.88	2.80
6	-11	3	287.17	4.40
6	-11	4	11.20	0.50
6	-12	-3	98.39	1.70
6	-12	-2	0.30	0.10
6	-12	-1	138.79	2.10
6	-12	0	4.10	0.50
6	-12	1	57.99	1.40
7	0	-12	332.37	5.00
7	0	-11	0.20	0.20
7	0	-10	53.29	1.40
7	0	-9	0.10	0.20
7	0	-8	193.28	3.40
7	0	-7	0.10	0.20
7	0	-6	1180.98	14.80
7	0	-5	0.70	0.20
7	0	-4	174.68	4.50
7	0	-3	1.70	0.90

7	0	-2	2671.63	48.30
7	0	-1	0.50	0.30
7	0	0	979.10	18.80
7	0	1	0.90	0.80
7	0	2	1191.38	22.80
7	0	3	0.20	0.20
7	0	4	125.09	2.00
7	0	5	0.30	0.20
7	0	6	122.49	2.20
7	0	7	0.40	0.20
7	0	8	11.00	0.70
7	0	9	0.10	0.20
7	0	10	142.09	2.40
7	-1	-12	109.49	1.50
7	-1	-11	0.30	0.10
7	-1	-10	181.28	2.20
7	-1	-9	327.37	3.20
7	-1	-8	63.39	1.10
7	-1	-7	235.38	2.60
7	-1	-6	432.76	4.60
7	-1	-5	17.00	0.60

7	-1	-4	290.07	4.60
7	-1	-3	21.30	1.00
7	-1	-2	30.30	1.10
7	-1	-1	177.38	3.20
7	-1	0	713.03	8.90
7	-1	1	2627.04	36.00
7	-1	2	341.77	5.10
7	-1	3	628.14	5.80
7	-1	4	1385.96	10.00
7	-1	5	73.19	1.30
7	-1	6	1.10	0.20
7	-1	7	199.08	2.50
7	-1	8	47.80	0.90
7	-1	9	0.10	0.10
7	-1	10	0.20	0.10
7	-2	-12	58.09	0.90
7	-2	-11	2.40	0.20
7	-2	-10	11.50	0.50
7	-2	-9	988.50	9.40
7	-2	-8	293.87	3.20
7	-2	-7	537.85	5.00

7	-2	-6	33.60	0.80
7	-2	-5	1208.48	11.10
7	-2	-4	107.79	2.20
7	-2	-3	116.49	2.40
7	-2	-2	18.70	0.90
7	-2	-1	155.48	3.10
7	-2	0	170.68	3.20
7	-2	1	1.10	0.30
7	-2	2	149.29	2.30
7	-2	3	35.70	0.80
7	-2	4	38.50	0.80
7	-2	5	41.10	0.90
7	-2	6	0.30	0.10
7	-2	7	523.15	5.10
7	-2	8	218.58	2.50
7	-2	9	59.19	0.90
7	-3	-12	32.80	0.70
7	-3	-11	142.19	1.90
7	-3	-10	401.76	4.70
7	-3	-9	175.68	2.30
7	-3	-8	71.59	1.20

7	-3	-7	885.31	7.80
7	-3	-6	55.29	1.00
7	-3	-5	983.50	9.00
7	-3	-4	1.80	0.20
7	-3	-3	450.35	6.70
7	-3	-2	23.40	1.00
7	-3	-1	9.30	0.70
7	-3	0	526.75	7.60
7	-3	1	484.85	5.80
7	-3	2	10.10	0.60
7	-3	3	45.60	0.90
7	-3	4	227.28	2.50
7	-3	5	1.80	0.20
7	-3	6	20.40	0.60
7	-3	7	140.59	1.90
7	-3	8	8.30	0.50
7	-3	9	520.55	5.90
7	-4	-12	109.89	2.40
7	-4	-11	57.89	1.10
7	-4	-10	96.29	1.40
7	-4	-9	180.08	2.40

7	-4	-8	42.70	0.90
7	-4	-7	25.50	0.70
7	-4	-6	6.70	0.50
7	-4	-5	60.59	1.20
7	-4	-4	103.99	1.70
7	-4	-3	60.89	1.30
7	-4	-2	337.27	4.20
7	-4	-1	78.29	1.40
7	-4	0	20.60	0.60
7	-4	1	700.73	6.30
7	-4	2	88.99	1.30
7	-4	3	585.74	5.40
7	-4	4	10.80	0.50
7	-4	5	520.05	5.20
7	-4	6	27.40	0.80
7	-4	7	205.38	2.50
7	-4	8	23.60	0.70
7	-4	9	98.99	1.60
7	-5	-11	3.90	0.40
7	-5	-10	122.69	2.10
7	-5	-9	0.20	0.10

7	-5	-8	452.15	5.30
7	-5	-7	96.99	1.50
7	-5	-6	1.50	0.20
7	-5	-5	27.40	0.80
7	-5	-4	274.57	3.00
7	-5	-3	876.61	8.20
7	-5	-2	8.50	0.50
7	-5	-1	54.79	1.00
7	-5	0	133.89	1.70
7	-5	1	163.88	2.00
7	-5	2	542.05	5.20
7	-5	3	60.59	1.20
7	-5	4	62.39	1.10
7	-5	5	90.09	1.50
7	-5	6	21.90	0.70
7	-5	7	153.28	2.10
7	-5	8	28.80	0.80
7	-5	9	16.90	0.50
7	-6	-11	14.00	0.70
7	-6	-10	99.29	1.60
7	-6	-9	46.70	1.00

7	-6	-8	341.67	4.00
7	-6	-7	12.30	0.60
7	-6	-6	7.30	0.50
7	-6	-5	217.48	3.00
7	-6	-4	1109.59	13.10
7	-6	-3	84.59	1.30
7	-6	-2	523.65	5.00
7	-6	-1	1566.14	12.50
7	-6	0	369.36	3.60
7	-6	1	20.10	0.60
7	-6	2	260.37	2.90
7	-6	3	16.80	0.70
7	-6	4	207.98	2.80
7	-6	5	38.30	0.90
7	-6	6	478.25	4.80
7	-6	7	9.00	0.60
7	-6	8	94.49	1.90
7	-7	-10	109.59	3.80
7	-7	-9	60.39	1.30
7	-7	-8	465.55	5.80
7	-7	-7	536.95	6.30

7	-7	-6	159.08	2.60
7	-7	-5	7.90	0.60
7	-7	-4	147.49	2.20
7	-7	-3	297.27	3.60
7	-7	-2	1043.30	9.30
7	-7	-1	75.49	1.20
7	-7	0	439.56	4.50
7	-7	1	473.55	4.60
7	-7	2	83.09	1.40
7	-7	3	59.19	1.20
7	-7	4	354.66	4.00
7	-7	5	162.68	2.20
7	-7	6	19.50	0.60
7	-7	7	5.90	0.40
7	-8	-9	235.38	3.70
7	-8	-8	30.60	1.00
7	-8	-7	1.50	0.20
7	-8	-6	24.90	0.90
7	-8	-5	324.27	4.40
7	-8	-4	166.48	2.20
7	-8	-3	247.18	2.80

7	-8	-2	793.82	7.40
7	-8	-1	310.97	3.40
7	-8	0	198.28	2.40
7	-8	1	0.30	0.10
7	-8	2	250.07	4.10
7	-8	3	226.18	2.90
7	-8	4	0.80	0.20
7	-8	5	179.58	2.60
7	-8	6	0.10	0.10
7	-8	7	108.59	1.80
7	-9	-8	40.30	1.10
7	-9	-7	0.50	0.10
7	-9	-6	93.59	1.80
7	-9	-5	221.58	3.30
7	-9	-4	14.80	0.70
7	-9	-3	2.40	0.30
7	-9	-2	15.90	0.60
7	-9	-1	137.09	1.80
7	-9	0	24.80	0.70
7	-9	1	328.27	3.90
7	-9	2	0.60	0.20

7	-9	3	50.99	1.00
7	-9	4	40.20	1.00
7	-9	5	63.69	1.20
7	-9	6	0.60	0.10
7	-10	-7	101.89	1.90
7	-10	-6	86.99	1.50
7	-10	-5	602.34	7.10
7	-10	-4	11.20	0.60
7	-10	-3	77.79	1.40
7	-10	-2	119.89	2.00
7	-10	-1	17.00	0.50
7	-10	0	249.08	2.80
7	-10	1	84.99	1.30
7	-10	2	6.30	0.40
7	-10	3	81.29	1.40
7	-10	4	22.00	0.60
7	-11	-5	14.80	0.70
7	-11	-4	118.19	2.10
7	-11	-3	23.20	0.60
7	-11	-2	147.09	1.90
7	-11	-1	11.30	0.40

7	-11	0	182.18	2.30
7	-11	1	0.30	0.20
7	-11	2	59.89	1.30
8	0	-12	51.59	1.50
8	0	-11	0.10	0.10
8	0	-10	49.30	1.40
8	0	-9	0.10	0.20
8	0	-8	3.80	0.40
8	0	-7	0.20	0.20
8	0	-6	43.40	1.20
8	0	-5	0.30	0.20
8	0	-4	54.29	1.50
8	0	-3	0.20	0.30
8	0	-2	2254.67	47.40
8	0	-1	0.10	0.40
8	0	0	30.50	1.40
8	0	1	0.30	0.20
8	0	2	63.49	1.50
8	0	3	0.10	0.20
8	0	4	25.80	1.10
8	0	5	0.20	0.20

8	0	6	126.29	2.60
8	0	7	0.10	0.20
8	0	8	130.79	2.40
8	0	9	0.00	0.10
8	-1	-12	284.77	3.50
8	-1	-11	0.30	0.10
8	-1	-10	180.98	2.50
8	-1	-9	116.29	1.70
8	-1	-8	530.75	5.00
8	-1	-7	279.27	3.40
8	-1	-6	608.94	5.50
8	-1	-5	427.56	3.90
8	-1	-4	1.30	0.20
8	-1	-3	412.86	5.30
8	-1	-2	425.06	6.60
8	-1	-1	2.70	0.40
8	-1	0	0.90	0.20
8	-1	1	63.49	1.20
8	-1	2	621.54	5.40
8	-1	3	0.60	0.10
8	-1	4	261.87	2.90

8	-1	5	286.77	2.90
8	-1	6	67.69	1.30
8	-1	7	45.60	0.90
8	-1	8	81.39	1.20
8	-1	9	63.19	1.00
8	-2	-12	0.10	0.10
8	-2	-11	36.10	0.80
8	-2	-10	217.38	2.70
8	-2	-9	22.90	0.90
8	-2	-8	707.93	6.60
8	-2	-7	608.44	5.90
8	-2	-6	66.59	1.10
8	-2	-5	388.66	4.00
8	-2	-4	550.04	5.30
8	-2	-3	131.29	2.00
8	-2	-2	222.98	3.10
8	-2	-1	278.27	4.00
8	-2	0	306.07	3.80
8	-2	1	1240.48	11.00
8	-2	2	801.32	6.80
8	-2	3	959.50	8.00

8	-2	4	339.37	3.40
8	-2	5	320.17	3.10
8	-2	6	60.59	1.30
8	-2	7	89.99	1.30
8	-2	8	392.36	3.80
8	-2	9	0.20	0.10
8	-3	-12	12.30	0.50
8	-3	-11	3.80	0.40
8	-3	-10	49.00	1.00
8	-3	-9	116.39	1.70
8	-3	-8	35.30	0.90
8	-3	-7	10.30	0.60
8	-3	-6	315.27	3.30
8	-3	-5	2467.65	21.60
8	-3	-4	178.28	2.20
8	-3	-3	61.99	1.40
8	-3	-2	24.20	0.90
8	-3	-1	492.35	5.10
8	-3	0	0.90	0.20
8	-3	1	174.58	2.10
8	-3	2	242.78	2.50

8	-3	3	523.35	5.10
8	-3	4	349.27	3.30
8	-3	5	1020.70	8.90
8	-3	6	9.80	0.50
8	-3	7	33.40	0.80
8	-3	8	16.80	0.50
8	-3	9	239.88	3.80
8	-4	-11	69.49	1.20
8	-4	-10	173.08	2.40
8	-4	-9	173.48	2.30
8	-4	-8	1.80	0.20
8	-4	-7	634.14	6.50
8	-4	-6	492.25	5.20
8	-4	-5	32.60	0.90
8	-4	-4	19.50	0.70
8	-4	-3	421.16	5.00
8	-4	-2	1.00	0.30
8	-4	-1	61.79	1.00
8	-4	0	6.80	0.50
8	-4	1	1.10	0.20
8	-4	2	363.56	3.60

8	-4	3	108.59	1.50
8	-4	4	99.39	1.50
8	-4	5	198.58	2.40
8	-4	6	0.80	0.10
8	-4	7	0.70	0.10
8	-4	8	96.29	1.60
8	-5	-11	10.60	0.50
8	-5	-10	53.79	1.10
8	-5	-9	0.30	0.10
8	-5	-8	16.90	0.70
8	-5	-7	8.80	0.50
8	-5	-6	86.69	1.40
8	-5	-5	103.99	1.70
8	-5	-4	652.53	7.40
8	-5	-3	117.49	1.60
8	-5	-2	794.02	7.10
8	-5	-1	41.90	0.90
8	-5	0	938.21	8.60
8	-5	1	897.61	9.40
8	-5	2	272.57	3.20
8	-5	3	1.40	0.20

8	-5	4	0.60	0.20
8	-5	5	25.50	0.80
8	-5	6	337.47	3.90
8	-5	7	6.60	0.50
8	-5	8	28.70	0.70
8	-6	-10	216.58	3.30
8	-6	-9	17.20	0.60
8	-6	-8	474.85	5.70
8	-6	-7	108.79	1.80
8	-6	-6	3.60	0.40
8	-6	-5	1.00	0.20
8	-6	-4	8.00	0.60
8	-6	-3	0.20	0.10
8	-6	-2	171.88	2.20
8	-6	-1	1.40	0.20
8	-6	0	837.02	7.60
8	-6	1	75.29	1.20
8	-6	2	3.60	0.30
8	-6	3	20.30	0.70
8	-6	4	126.79	1.90
8	-6	5	0.20	0.10

8	-6	6	22.90	0.70
8	-6	7	0.30	0.10
8	-7	-10	69.09	1.70
8	-7	-9	43.00	1.00
8	-7	-8	50.39	1.10
8	-7	-7	6.90	0.50
8	-7	-6	485.45	6.10
8	-7	-5	28.10	0.90
8	-7	-4	44.60	1.10
8	-7	-3	21.30	0.70
8	-7	-2	577.84	5.80
8	-7	-1	27.50	0.80
8	-7	0	26.00	0.70
8	-7	1	547.15	5.60
8	-7	2	164.58	2.30
8	-7	3	106.29	1.60
8	-7	4	18.40	0.60
8	-7	5	74.69	1.40
8	-7	6	97.59	1.40
8	-7	7	7.90	0.40
8	-8	-9	15.90	0.80

8	-8	-8	109.79	1.90
8	-8	-7	8.10	0.50
8	-8	-6	19.70	0.70
8	-8	-5	139.99	2.20
8	-8	-4	79.89	1.50
8	-8	-3	241.58	3.10
8	-8	-2	504.35	6.30
8	-8	-1	71.59	1.50
8	-8	0	76.09	1.20
8	-8	1	372.26	4.10
8	-8	2	2.10	0.20
8	-8	3	13.70	0.50
8	-8	4	32.70	0.90
8	-8	5	27.90	0.80
8	-8	6	1.10	0.10
8	-9	-8	0.60	0.20
8	-9	-7	138.39	2.30
8	-9	-6	9.90	0.50
8	-9	-5	1124.79	13.60
8	-9	-4	0.20	0.10
8	-9	-3	59.09	1.10

8	-9	-2	90.19	1.40
8	-9	-1	41.70	0.80
8	-9	0	0.30	0.10
8	-9	1	0.70	0.10
8	-9	2	265.17	3.30
8	-9	3	240.88	3.20
8	-9	4	141.79	2.30
8	-9	5	81.89	1.30
8	-10	-6	31.60	0.80
8	-10	-5	0.00	0.10
8	-10	-4	100.99	1.60
8	-10	-3	46.30	0.90
8	-10	-2	249.78	2.90
8	-10	-1	42.20	0.90
8	-10	0	273.67	3.50
8	-10	1	404.56	4.60
8	-10	2	0.20	0.10
8	-10	3	4.90	0.40
8	-11	-4	116.49	2.30
8	-11	-3	0.90	0.20
8	-11	-2	65.79	1.40

8	-11	-1	5.70	0.50
8	-11	0	5.50	0.50
9	0	-12	633.04	10.70
9	0	-11	0.10	0.10
9	0	-10	391.66	6.20
9	0	-9	0.20	0.20
9	0	-8	690.33	9.10
9	0	-7	0.20	0.20
9	0	-6	2686.43	32.50
9	0	-5	0.10	0.20
9	0	-4	531.95	7.30
9	0	-3	0.50	0.20
9	0	-2	186.98	3.70
9	0	-1	0.10	0.30
9	0	0	481.75	8.20
9	0	1	0.60	0.30
9	0	2	1776.52	19.30
9	0	3	0.20	0.20
9	0	4	296.37	3.90
9	0	5	0.10	0.20
9	0	6	622.14	8.30

9	0	7	0.10	0.20
9	0	8	242.48	3.40
9	-1	-12	11.60	0.40
9	-1	-11	20.80	0.60
9	-1	-10	57.09	1.00
9	-1	-9	52.09	1.00
9	-1	-8	722.03	7.00
9	-1	-7	109.79	1.60
9	-1	-6	152.88	2.00
9	-1	-5	124.79	1.50
9	-1	-4	1.90	0.20
9	-1	-3	362.46	4.40
9	-1	-2	705.73	7.50
9	-1	-1	1.50	0.20
9	-1	0	760.42	7.40
9	-1	1	95.19	1.40
9	-1	2	12.40	0.60
9	-1	3	680.43	6.10
9	-1	4	201.88	2.10
9	-1	5	0.20	0.10
9	-1	6	34.70	0.70

9	-1	7	413.16	4.30
9	-1	8	54.59	0.90
9	-2	-11	113.49	1.50
9	-2	-10	0.40	0.10
9	-2	-9	57.29	1.10
9	-2	-8	0.30	0.10
9	-2	-7	52.39	1.00
9	-2	-6	372.46	3.90
9	-2	-5	144.59	1.90
9	-2	-4	163.78	2.10
9	-2	-3	212.68	2.50
9	-2	-2	21.40	0.70
9	-2	-1	221.78	2.60
9	-2	0	462.05	4.70
9	-2	1	138.39	1.80
9	-2	2	160.58	2.10
9	-2	3	0.70	0.10
9	-2	4	41.40	0.80
9	-2	5	0.50	0.10
9	-2	6	0.30	0.10
9	-2	7	122.29	1.80

9	-2	8	0.10	0.10
9	-3	-11	217.78	3.20
9	-3	-10	7.10	0.40
9	-3	-9	100.59	1.50
9	-3	-8	291.97	4.20
9	-3	-7	76.79	1.30
9	-3	-6	175.38	2.30
9	-3	-5	22.20	0.80
9	-3	-4	436.86	4.80
9	-3	-3	17.10	0.70
9	-3	-2	147.19	1.90
9	-3	-1	377.26	3.90
9	-3	0	76.39	1.20
9	-3	1	41.40	1.00
9	-3	2	318.67	3.50
9	-3	3	365.36	3.80
9	-3	4	136.19	1.90
9	-3	5	561.34	5.60
9	-3	6	0.30	0.10
9	-3	7	4.20	0.40
9	-3	8	0.60	0.10

9	-4	-11	74.39	1.40
9	-4	-10	3.90	0.50
9	-4	-9	0.50	0.10
9	-4	-8	54.49	1.20
9	-4	-7	110.79	1.80
9	-4	-6	46.90	1.10
9	-4	-5	1143.59	10.90
9	-4	-4	1.40	0.20
9	-4	-3	364.16	4.20
9	-4	-2	0.90	0.30
9	-4	-1	819.42	7.70
9	-4	0	5.00	0.50
9	-4	1	7.10	0.50
9	-4	2	848.32	7.90
9	-4	3	687.93	6.90
9	-4	4	224.88	3.20
9	-4	5	564.04	5.80
9	-4	6	10.30	0.50
9	-4	7	6.30	0.40
9	-5	-10	341.47	4.50
9	-5	-9	1.10	0.20

9	-5	-8	219.68	3.00
9	-5	-7	248.68	3.50
9	-5	-6	66.39	1.40
9	-5	-5	44.30	1.00
9	-5	-4	744.83	7.50
9	-5	-3	532.25	6.00
9	-5	-2	20.20	0.80
9	-5	-1	299.27	3.40
9	-5	0	53.69	1.10
9	-5	1	168.08	2.20
9	-5	2	40.70	0.90
9	-5	3	13.40	0.70
9	-5	4	99.39	1.70
9	-5	5	328.97	4.00
9	-5	6	0.20	0.10
9	-5	7	23.50	0.60
9	-6	-10	25.80	0.80
9	-6	-9	49.80	1.20
9	-6	-8	209.28	2.90
9	-6	-7	8.10	0.60
9	-6	-6	19.70	0.70

9	-6	-5	283.77	3.80
9	-6	-4	191.98	2.60
9	-6	-3	316.57	3.50
9	-6	-2	48.10	1.00
9	-6	-1	164.88	2.10
9	-6	0	81.69	1.30
9	-6	1	91.79	1.50
9	-6	2	399.06	5.40
9	-6	3	76.89	1.20
9	-6	4	46.10	0.90
9	-6	5	50.09	1.00
9	-6	6	111.99	1.60
9	-7	-9	3.60	0.40
9	-7	-8	5.40	0.40
9	-7	-7	49.90	1.10
9	-7	-6	0.50	0.10
9	-7	-5	24.00	0.80
9	-7	-4	31.90	0.80
9	-7	-3	475.35	6.10
9	-7	-2	1141.69	12.60
9	-7	-1	0.30	0.20

9	-7	0	456.55	5.00
9	-7	1	56.69	1.10
9	-7	2	16.90	0.60
9	-7	3	14.30	0.60
9	-7	4	49.50	1.10
9	-7	5	45.10	0.90
9	-7	6	48.00	0.90
9	-8	-8	63.29	1.50
9	-8	-7	37.20	0.90
9	-8	-6	22.20	0.80
9	-8	-5	496.65	6.40
9	-8	-4	118.19	1.80
9	-8	-3	21.00	0.70
9	-8	-2	26.00	0.70
9	-8	-1	93.59	1.40
9	-8	0	6.20	0.50
9	-8	1	8.20	0.50
9	-8	2	9.70	0.50
9	-8	3	175.08	2.30
9	-8	4	23.50	0.70
9	-8	5	87.19	1.40

9	-9	-7	109.69	1.80
9	-9	-6	76.79	1.50
9	-9	-5	114.79	2.00
9	-9	-4	0.40	0.10
9	-9	-3	233.58	3.00
9	-9	-2	14.00	0.70
9	-9	-1	0.30	0.10
9	-9	0	0.20	0.20
9	-9	1	248.18	3.10
9	-9	2	64.19	1.20
9	-9	3	530.95	6.50
9	-10	-5	194.58	2.80
9	-10	-4	8.40	0.40
9	-10	-3	1.00	0.20
9	-10	-2	72.69	1.50
9	-10	-1	34.40	1.30
9	-10	0	4.90	0.50
9	-10	1	8.30	0.50
10	0	-11	0.10	0.10
10	0	-10	197.28	3.50
10	0	-9	0.20	0.20

10	0	-8	344.27	5.70
10	0	-7	0.20	0.20
10	0	-6	59.19	1.50
10	0	-5	0.50	0.20
10	0	-4	61.59	1.90
10	0	-3	0.30	0.20
10	0	-2	398.76	7.10
10	0	-1	0.40	0.30
10	0	0	759.32	12.30
10	0	1	0.30	0.30
10	0	2	290.87	4.60
10	0	3	0.20	0.20
10	0	4	0.60	0.20
10	0	5	0.00	0.20
10	0	6	101.69	2.20
10	0	7	0.00	0.20
10	-1	-11	81.59	1.40
10	-1	-10	142.89	2.10
10	-1	-9	291.07	3.30
10	-1	-8	225.58	2.70
10	-1	-7	0.80	0.20

10	-1	-6	1137.99	9.60
10	-1	-5	3.10	0.30
10	-1	-4	10.70	0.60
10	-1	-3	33.60	0.90
10	-1	-2	47.10	1.00
10	-1	-1	0.40	0.20
10	-1	0	243.68	3.50
10	-1	1	2.70	0.20
10	-1	2	779.62	8.60
10	-1	3	314.97	3.60
10	-1	4	429.36	4.50
10	-1	5	451.05	4.60
10	-1	6	46.10	1.20
10	-1	7	282.17	3.90
10	-2	-11	60.79	1.10
10	-2	-10	237.78	2.70
10	-2	-9	0.30	0.10
10	-2	-8	304.97	3.70
10	-2	-7	508.05	5.60
10	-2	-6	119.59	1.60
10	-2	-5	392.46	4.00

10	-2	-4	593.44	6.00
10	-2	-3	1136.99	11.60
10	-2	-2	225.88	3.10
10	-2	-1	143.09	2.10
10	-2	0	100.09	1.70
10	-2	1	209.28	2.40
10	-2	2	589.34	5.60
10	-2	3	8.60	0.60
10	-2	4	6.30	0.60
10	-2	5	681.53	7.50
10	-2	6	65.59	1.20
10	-2	7	149.88	2.00
10	-3	-11	84.09	1.40
10	-3	-10	8.60	0.50
10	-3	-9	15.00	0.60
10	-3	-8	0.30	0.10
10	-3	-7	25.50	0.90
10	-3	-6	7.70	0.60
10	-3	-5	1381.56	12.40
10	-3	-4	588.54	6.20
10	-3	-3	8.60	0.60

10	-3	-2	4.00	0.40
10	-3	-1	830.12	8.30
10	-3	0	0.30	0.20
10	-3	1	253.47	2.80
10	-3	2	121.19	1.70
10	-3	3	401.66	4.50
10	-3	4	0.10	0.10
10	-3	5	129.19	1.80
10	-3	6	0.50	0.10
10	-3	7	13.00	0.50
10	-4	-10	29.70	0.70
10	-4	-9	6.90	0.50
10	-4	-8	103.29	1.80
10	-4	-7	69.69	1.30
10	-4	-6	130.89	2.30
10	-4	-5	267.87	3.40
10	-4	-4	492.55	5.50
10	-4	-3	57.89	1.20
10	-4	-2	25.60	0.80
10	-4	-1	129.79	1.80
10	-4	0	11.10	0.60

10	-4	1	175.58	2.30
10	-4	2	277.77	3.60
10	-4	3	4.90	0.40
10	-4	4	65.89	1.10
10	-4	5	529.65	6.50
10	-4	6	0.10	0.10
10	-5	-10	4.90	0.40
10	-5	-9	57.49	1.20
10	-5	-8	339.17	4.60
10	-5	-7	120.29	2.00
10	-5	-6	54.09	1.30
10	-5	-5	22.00	1.00
10	-5	-4	197.18	2.90
10	-5	-3	458.15	5.60
10	-5	-2	272.17	3.30
10	-5	-1	58.39	1.20
10	-5	0	382.96	4.30
10	-5	1	0.60	0.20
10	-5	2	250.27	3.00
10	-5	3	93.39	1.40
10	-5	4	37.00	0.80

10	-5	5	0.20	0.10
10	-5	6	74.19	1.40
10	-6	-9	0.20	0.20
10	-6	-8	217.38	3.30
10	-6	-7	14.00	0.60
10	-6	-6	118.59	2.10
10	-6	-5	49.70	1.20
10	-6	-4	197.78	2.50
10	-6	-3	6.70	0.70
10	-6	-2	147.69	2.30
10	-6	-1	28.70	0.80
10	-6	0	334.27	3.80
10	-6	1	0.60	0.20
10	-6	2	115.19	1.70
10	-6	3	26.50	0.70
10	-6	4	302.77	3.50
10	-6	5	29.00	0.70
10	-7	-8	124.89	2.80
10	-7	-7	12.50	0.70
10	-7	-6	89.99	1.90
10	-7	-5	37.70	1.00

10	-7	-4	65.59	1.20
10	-7	-3	25.90	0.80
10	-7	-2	22.30	0.80
10	-7	-1	73.59	1.30
10	-7	0	87.29	1.50
10	-7	1	0.90	0.20
10	-7	2	15.60	0.60
10	-7	3	145.29	2.00
10	-7	4	198.28	2.50
10	-8	-7	29.70	0.80
10	-8	-6	20.00	0.80
10	-8	-5	16.70	0.70
10	-8	-4	8.70	0.50
10	-8	-3	234.88	3.10
10	-8	-2	118.09	2.50
10	-8	-1	9.00	0.60
10	-8	0	267.77	3.10
10	-8	1	184.08	2.30
10	-8	2	75.29	1.30
10	-8	3	116.99	1.90
10	-9	-6	0.10	0.10

10	-9	-5	129.29	2.20
10	-9	-4	38.40	1.00
10	-9	-3	93.99	1.60
10	-9	-2	39.60	1.00
10	-9	-1	78.89	1.60
10	-9	0	22.70	1.00
10	-9	1	72.39	1.50
10	-9	2	23.20	0.70
11	0	-10	0.60	0.20
11	0	-9	0.10	0.20
11	0	-8	278.77	4.60
11	0	-7	0.20	0.20
11	0	-6	1294.67	17.00
11	0	-5	0.20	0.20
11	0	-4	171.28	3.30
11	0	-3	0.00	0.20
11	0	-2	15.50	1.10
11	0	-1	0.20	0.20
11	0	0	957.10	15.10
11	0	1	0.20	0.30
11	0	2	612.84	11.00

11	0	3	0.10	0.20
11	0	4	224.38	4.00
11	0	5	0.10	0.20
11	0	6	27.00	1.30
11	-1	-10	217.38	3.10
11	-1	-9	4.60	0.50
11	-1	-8	4.30	0.40
11	-1	-7	1.20	0.20
11	-1	-6	476.45	5.30
11	-1	-5	49.10	1.00
11	-1	-4	79.79	1.40
11	-1	-3	1207.88	13.20
11	-1	-2	182.78	2.50
11	-1	-1	425.66	5.30
11	-1	0	135.09	2.10
11	-1	1	27.40	0.90
11	-1	2	95.89	1.80
11	-1	3	101.89	1.50
11	-1	4	536.25	6.00
11	-1	5	4.30	0.50
11	-1	6	32.50	0.90

11	-2	-10	20.40	0.60
11	-2	-9	502.45	8.50
11	-2	-8	55.09	1.20
11	-2	-7	3.30	0.40
11	-2	-6	78.89	1.50
11	-2	-5	0.90	0.20
11	-2	-4	9.30	0.60
11	-2	-3	0.50	0.20
11	-2	-2	13.30	0.70
11	-2	-1	262.87	3.40
11	-2	0	46.20	1.10
11	-2	1	94.59	1.60
11	-2	2	194.68	2.40
11	-2	3	0.90	0.20
11	-2	4	5.90	0.50
11	-2	5	62.49	1.00
11	-2	6	20.70	0.60
11	-3	-10	13.80	0.50
11	-3	-9	17.30	0.60
11	-3	-8	17.40	0.70
11	-3	-7	99.29	1.60

11	-3	-6	119.99	1.90
11	-3	-5	75.89	1.50
11	-3	-4	87.99	1.60
11	-3	-3	645.44	7.00
11	-3	-2	105.49	2.00
11	-3	-1	5.20	0.60
11	-3	0	11.60	0.60
11	-3	1	8.30	0.60
11	-3	2	11.10	0.50
11	-3	3	41.30	0.90
11	-3	4	375.76	4.10
11	-3	5	446.36	5.00
11	-3	6	6.50	0.40
11	-4	-10	18.30	0.70
11	-4	-9	81.39	1.40
11	-4	-8	154.98	2.50
11	-4	-7	220.08	2.90
11	-4	-6	32.40	0.80
11	-4	-5	504.45	5.80
11	-4	-4	499.65	6.50
11	-4	-3	14.80	0.80

11	-4	-2	181.08	2.80
11	-4	-1	374.76	5.60
11	-4	0	174.58	2.50
11	-4	1	67.99	1.20
11	-4	2	314.67	3.70
11	-4	3	248.28	2.80
11	-4	4	4.50	0.40
11	-4	5	0.20	0.10
11	-5	-9	0.30	0.10
11	-5	-8	53.29	1.10
11	-5	-7	74.19	1.50
11	-5	-6	8.50	0.50
11	-5	-5	24.30	0.70
11	-5	-4	30.30	0.90
11	-5	-3	527.45	6.80
11	-5	-2	53.49	1.20
11	-5	-1	68.49	1.50
11	-5	0	132.39	2.10
11	-5	1	10.70	0.50
11	-5	2	272.27	3.20
11	-5	3	0.20	0.10

11	-5	4	60.49	1.00
11	-5	5	1.20	0.10
11	-6	-8	132.89	2.20
11	-6	-7	8.10	0.40
11	-6	-6	328.47	4.50
11	-6	-5	0.60	0.20
11	-6	-4	331.87	4.40
11	-6	-3	83.39	1.60
11	-6	-2	72.89	1.40
11	-6	-1	316.17	3.90
11	-6	0	32.90	1.10
11	-6	1	11.40	0.60
11	-6	2	167.78	2.30
11	-6	3	18.80	0.60
11	-6	4	207.78	2.70
11	-7	-7	37.50	0.80
11	-7	-6	23.40	0.80
11	-7	-5	6.80	0.50
11	-7	-4	30.80	0.90
11	-7	-3	55.49	1.20
11	-7	-2	61.39	1.50

11	-7	-1	8.00	0.50
11	-7	0	385.26	5.40
11	-7	1	30.60	1.00
11	-7	2	3.50	0.50
11	-7	3	7.00	0.40
11	-8	-6	78.49	1.70
11	-8	-5	193.88	3.10
11	-8	-4	32.20	0.90
11	-8	-3	130.79	2.10
11	-8	-2	68.79	1.30
11	-8	-1	153.88	2.50
11	-8	0	212.18	3.80
11	-8	1	68.59	1.30
11	-9	-3	81.99	2.50
11	-9	-2	3.80	0.50
12	0	-10	82.19	2.10
12	0	-9	0.10	0.20
12	0	-8	4.10	0.80
12	0	-7	0.30	0.20
12	0	-6	572.44	10.10
12	0	-5	0.00	0.20

12	0	-4	27.90	1.50
12	0	-3	0.50	0.50
12	0	-2	38.80	2.30
12	0	-1	0.20	0.20
12	0	0	249.88	5.20
12	0	1	0.20	0.20
12	0	2	20.90	1.10
12	0	3	0.10	0.20
12	0	4	462.95	7.10
12	0	5	0.10	0.20
12	-1	-10	0.20	0.10
12	-1	-9	108.29	1.90
12	-1	-8	193.38	2.90
12	-1	-7	20.50	0.70
12	-1	-6	60.39	1.20
12	-1	-5	0.40	0.20
12	-1	-4	26.80	0.90
12	-1	-3	514.05	7.80
12	-1	-2	65.09	1.70
12	-1	-1	2.60	0.30
12	-1	0	420.06	5.30

12	-1	1	25.30	0.90
12	-1	2	141.59	2.30
12	-1	3	10.10	0.50
12	-1	4	8.80	0.50
12	-1	5	66.99	1.50
12	-2	-9	1.40	0.20
12	-2	-8	67.19	1.20
12	-2	-7	114.39	1.70
12	-2	-6	60.09	1.10
12	-2	-5	153.78	2.10
12	-2	-4	89.09	1.60
12	-2	-3	589.04	7.40
12	-2	-2	133.49	2.10
12	-2	-1	151.68	2.20
12	-2	0	78.19	1.60
12	-2	1	25.50	0.90
12	-2	2	38.60	0.90
12	-2	3	81.59	1.60
12	-2	4	141.99	2.20
12	-2	5	198.38	3.70
12	-3	-9	139.89	2.40

12	-3	-8	5.60	0.50
12	-3	-7	3.70	0.40
12	-3	-6	68.09	1.30
12	-3	-5	167.38	2.30
12	-3	-4	196.08	2.90
12	-3	-3	1.10	0.20
12	-3	-2	11.50	0.70
12	-3	-1	435.06	5.30
12	-3	0	0.00	0.20
12	-3	1	185.58	2.80
12	-3	2	213.68	3.00
12	-3	3	89.99	1.60
12	-3	4	2.00	0.40
12	-4	-9	18.10	0.60
12	-4	-8	0.30	0.10
12	-4	-7	68.49	1.10
12	-4	-6	0.30	0.10
12	-4	-5	34.80	0.90
12	-4	-4	332.57	4.40
12	-4	-3	192.28	3.10
12	-4	-2	95.79	1.90

12	-4	-1	1.90	0.20
12	-4	0	31.20	0.90
12	-4	1	95.59	1.90
12	-4	2	21.00	0.80
12	-4	3	1.00	0.20
12	-4	4	15.10	1.10
12	-5	-8	291.97	4.30
12	-5	-7	53.59	1.10
12	-5	-6	234.88	3.00
12	-5	-5	93.79	1.80
12	-5	-4	43.60	1.20
12	-5	-3	41.00	1.10
12	-5	-2	194.28	3.10
12	-5	-1	67.19	1.60
12	-5	0	392.26	5.90
12	-5	1	0.40	0.10
12	-5	2	168.08	2.80
12	-5	3	55.69	1.40
12	-6	-7	57.49	1.40
12	-6	-6	309.27	4.60
12	-6	-5	25.60	0.80

12	-6	-4	21.80	0.80
12	-6	-3	29.00	1.10
12	-6	-2	225.38	3.40
12	-6	-1	0.90	0.20
12	-6	0	0.60	0.10
12	-6	1	57.69	1.20
12	-6	2	319.87	4.70
12	-7	-6	54.59	1.50
12	-7	-5	74.79	2.10
12	-7	-4	0.00	0.20
12	-7	-3	5.60	0.50
12	-7	-2	4.40	0.40
12	-7	-1	17.30	0.80
12	-7	0	22.70	0.80
12	-7	1	199.98	3.40
13	0	-8	221.08	5.00
13	0	-7	0.10	0.20
13	0	-6	137.69	3.50
13	0	-5	0.00	0.20
13	0	-4	165.18	4.20
13	0	-3	0.00	0.20

13	0	-2	58.29	2.10
13	0	-1	0.00	0.20
13	0	0	27.90	1.20
13	0	1	0.00	0.30
13	0	2	402.66	7.30
13	0	3	0.20	0.20
13	-1	-8	134.79	2.50
13	-1	-7	8.90	0.60
13	-1	-6	80.89	1.60
13	-1	-5	0.30	0.10
13	-1	-4	5.20	0.60
13	-1	-3	292.67	4.60
13	-1	-2	131.19	2.30
13	-1	-1	266.47	3.90
13	-1	0	2.10	0.30
13	-1	1	29.20	0.90
13	-1	2	6.40	0.40
13	-1	3	0.10	0.20
13	-2	-8	8.10	0.50
13	-2	-7	0.10	0.20
13	-2	-6	5.50	0.60

13	-2	-5	47.90	1.20
13	-2	-4	181.68	3.10
13	-2	-3	6.60	0.60
13	-2	-2	5.60	0.60
13	-2	-1	416.06	5.70
13	-2	0	55.69	1.30
13	-2	1	1.10	0.20
13	-2	2	175.98	2.60
13	-2	3	58.39	1.50
13	-3	-8	22.40	1.20
13	-3	-7	20.10	0.70
13	-3	-6	9.60	0.70
13	-3	-5	26.00	1.10
13	-3	-4	0.80	0.20
13	-3	-3	298.97	4.30
13	-3	-2	45.90	1.10
13	-3	-1	1.00	0.20
13	-3	0	20.30	0.80
13	-3	1	190.58	2.80
13	-3	2	0.20	0.10
13	-3	3	26.70	0.80

13	-4	-7	76.19	1.70
13	-4	-6	23.10	0.90
13	-4	-5	521.95	8.10
13	-4	-4	8.40	0.60
13	-4	-3	29.50	1.00
13	-4	-2	6.40	0.50
13	-4	-1	79.29	1.70
13	-4	0	30.50	0.80
13	-4	1	5.20	0.40
13	-4	2	30.80	0.80
13	-5	-6	349.47	6.00
13	-5	-5	26.80	1.00
13	-5	-4	65.09	1.60
13	-5	-3	42.60	1.10
13	-5	-2	0.90	0.20
13	-5	-1	51.99	1.10
13	-5	0	45.10	1.20
13	-5	1	164.58	2.80
13	-6	-5	7.70	0.50
13	-6	-4	38.10	1.20
13	-6	-3	33.70	0.90

13	-6	-2	103.49	2.00
13	-6	-1	39.70	0.90
13	-6	0	20.90	0.70
14	0	-7	0.00	0.20
14	0	-6	3.90	0.70
14	0	-5	0.10	0.20
14	0	-4	24.00	1.10
14	0	-3	0.20	0.20
14	0	-2	542.65	11.00
14	0	-1	0.00	0.20
14	0	0	25.20	0.90
14	0	1	0.10	0.40
14	-1	-7	19.50	0.80
14	-1	-6	71.09	2.40
14	-1	-5	48.50	1.30
14	-1	-4	23.90	0.80
14	-1	-3	23.70	0.80
14	-1	-2	89.39	1.70
14	-1	-1	9.50	0.60
14	-1	0	214.88	3.30
14	-1	1	3.10	0.30

14	-2	-6	0.90	0.20
14	-2	-5	114.89	2.50
14	-2	-4	10.20	0.60
14	-2	-3	255.97	4.30
14	-2	-2	93.69	1.90
14	-2	-1	31.80	1.00
14	-2	0	8.30	0.60
14	-2	1	18.30	0.60
14	-3	-6	22.00	1.10
14	-3	-5	219.78	4.70
14	-3	-4	112.39	2.90
14	-3	-3	0.80	0.20
14	-3	-2	18.90	1.10
14	-3	-1	73.69	1.80
14	-3	0	0.10	0.20
14	-4	-5	0.10	0.10
14	-4	-4	66.19	1.90
14	-4	-3	21.40	0.80
14	-4	-2	2.80	0.40
14	-4	-1	24.00	0.80
0	0	0	0.00	0.00

_computing_structure_solution 'SHELXT 2018/2 (Sheldrick, 2018)'

;

_shelx_hkl_checksum 617
